# Morphological character evolution and ancestral state reconstruction in phylactolaemate bryozoans

**DOI:** 10.1038/s41598-026-40223-0

**Published:** 2026-03-23

**Authors:** Julian Bibermair, Ahmed J. Saadi, Thomas Schwaha

**Affiliations:** Department of Evolutionary Biology, University of Vienna, Vienna, Austria

**Keywords:** Ground pattern reconstruction, Character tracing, Light microscopy, Confocal microscopy, Evolution, Zoology

## Abstract

Phylactolaemates represent a small clade within bryozoans that exclusively occur in limnic habitats. About 100 species are described in seven to eight families. Since they are sister taxon to the extensive clade of myolaemate bryozoans, understanding their evolution is crucial for all bryozoans. In recent years, advances in imaging and phylogenetics enabled detailed analysis of the morphology of phylactolaemate families and their internal relationship. Also, historical analyses provide profound data of phylactolaemate lifestyle and development and represent the base of modern phylactolaemate research. Previous analysis primarily focused on sole families and rarely included a broader range of phylactolaemates. This study provides the first comprehensive and comparative analysis of the morphology of this group. It combines historical data as well as recent ones and complements new data wherever possible, to fill in gaps in our knowledge. The combined data were used to establish a morphological character matrix which is mapped onto a recent molecular phylogeny. The ancestral state reconstruction allowed to trace the evolution of numerous traits. The results reveal colonies with large interzooidal distances and non-encrusting cystids ancestral. Moreover, it shows that the myoanatomy is largely conserved but several lineages show unique adaptations in detail, especially in the complex lophophoral base. The neuroanatomy is remarkedly conserved across all taxa. Statoblast types and traits concerning the ectocyst, capability of locomotion and some myoanatomical adaptations are likely to have independent evolutionary origins. Overall, the morphological evolution of phylactolaemates reflects both, deep homology and frequent lineage-specific adaptations. Consequently, it is emphasized to integrate ecological and functional perspectives in future analysis.

**Supplementary Information:**

The online version contains supplementary material available at 10.1038/s41598-026-40223-0.

## Introduction

Bryozoans are colonial, aquatic and benthic lophotrochozoans. Together with phoronids and brachiopods they form the clade of Lophophorata within the latter^1,2^. Morphologically, this hypothesis has been established based on several characters such as the general coelomate body organization, a ciliated tentacle crown or lophophore, and the epistome, which is a flap-like protrusion above the mouth. The latter is present in phoronids, brachiopods and phylactolaemate bryozoans. Recent molecular studies find support for the concept with phoronids being the sister taxon of bryozoans^2^. Bryozoans comprise two major clades: Phylactolaemata and Myolaemata. The latter includes Stenolaemata (Cyclostomata) and Gymnolaemata^1,3–6^. Phylactolaemata represents the sister taxon to Myolaemata^1,3,6,7^. With about 100 species, phylactolaemates constitute a rather small group of bryozoans that occur mostly in freshwater habitats^5,8,9^. As in all bryozoans, colonies are formed by numerous asexually formed zooids, each consisting of a protective body wall (cystid) and a retractable polypide. In phylactolaemates, the ectocyst, secreted from the epidermis, is either gelatinous or encrusted, as it agglutinates particles from the environment^10,11^. Contrary to myolaemates, which often show calcification of their body walls^1,3^, phylactolaemates always retain unmineralized cystid walls.

The polypide, in general, includes the lophophore with approximately 15–80 ciliated tentacles - depending on the family. The number of tentacles in phylactolaemates is not of systematic relevance but rather correlates with the size of the lophophore, which varies among families. Even within a species, the tentacle number is not consistent and can vary approx. between 10 and 25%^12–21^. At the lophophoral base there is a mouth opening, which continues into a U-shaped digestive tract. Phylactolaemates are the only bryozoans to possess an epistome, which is located at the lophophoral base. Outside, its epidermis is heavily ciliated and the phylactolaemate epistome always contains a coelomic cavity, lined by a peritoneal layer. The organ is missing or reduced in some phoronids and brachiopods^3,5,8,22–27^. The epistome probably has a sensory function and is involved in feeding, but direct evidence of its function remains elusive (e.g. Wood^8^.

Two structures connect the polypide with the cystid via the coelomic cavity. Prominent retractor muscles serve as antagonist to the orthogonal grid of body wall musculature. Contraction of the latter leads to an increase of the coelomic pressure and consequently polypide protrusion. The funiculus, a thin peritoneal strand, connects the proximal tip of the digestive tract to the basal body wall and includes longitudinal muscle in all phylactolaemates^28,29^.

In protruded condition, the lophophore proximally continues into the tentacle sheath, which itself continues into the vestibular wall. The vestibular wall usually does not protrude and remains slightly invaginated in respect to the distally adjoining cystid wall^14,28^. Upon retraction of the polypide, the tentacle sheath introverts and thus extends proximally of the vestibular wall to enclose the retracted lophophore. At the transition of the tentacle sheath and vestibular wall, lies a sphincter muscle. Antagonistic to the latter are a series of apertural muscles traversing the coelomic cavity from the vestibular wall and tentacle sheath to the lateral body wall^11,14,15,18,28,30,31^.

Seven to eight recent families are recognized among phylactolaemates^7,32–34^. At least four of them (Stephanellidae, Cristatellidae, Pectinatellidae, Hirosellidae) are monotypic with only one genus and species. Sister-taxon to all other phylactolaemates is Stephanellidae with *Stephanella hina*. It has serially arranged zooids with lateral buds and a unique ectocyst not attached to the epidermis^35–38^. The lophopodids comprise three genera with gelatinous colonies and clustered zooids^39–44^. Lophopodids are the sister taxon to all other non-stephanellid phylactolaemates. The remaining phylactolaemates are subdivided into two larger clades. One of them is Plumatellidae^7^. With more than 60 species, they are also the most speciose group of phylactolaemates and include several genera^11,12,45^, which form colonies of serially arranged zooids^46–48^. In general, plumatellids look rather similar but myoanatomical details such as the epistome musculature, tentacle muscle bases and size of the lophophore may differ. Recent molecular data provide strong support for monophyletic plumatellids, although internal systematics will require additional studies.

The sister clade of plumatellids comprises four families: Fredericellidae and a clade formerly known as PCP-clade (now HCP-clade) which includes Hirosellidae (originally as a species of *Plumatella* ‘P’, now ‘H’), Cristatellidae (‘C’) and Pectinatellidae (‘P’). These are monotypic and share several morphological features such as colony structure or details and size of individual zooids, whereas *Hirosella fruticosa* shares a mosaic of fredericellid and plumatellid characters^13,15^, but also morphological characters with Cristatellidae and Pectinatellidae^49^. This unique set of characters and its phylogenetic position of *H. fruticosa* confirmed its non-plumatellid affinity and called for a new family, Hirosellidae, which probably will also house other plumatellids, e.g. *P. osbourni*^49,50^, once sequence data will be available. The eighth family of phylactolaemates, referred to as Tapajosellidae, is only known from statoblasts with no available sequence data^34^.

Statoblasts are apomorphic for Phylactolaemata and are internal, asexual buds produced within protective coverings that serve overwintering and dispersal. They are adaptations to freshwater habitats, which often show seasonality and unfavourable conditions in winter^1,11^. Statoblasts are formed at the funiculus and always have a central capsule filled with yolk and germinative tissue. The capsule is surrounded by a periblast of various form, often as a gas-filled float or annulus. As internal buds, statoblasts are essential for creating new colonies by asexual budding after e.g. a phase of draught or low temperatures^8,11,51^. Statoblast morphology has been used for systematic purposes and remains a crucial tool for species identification^12,34,45,51–54^.

The last two decades propelled phylactolaemate research by applying state-of-the-art techniques such as fluorescence staining and confocal laser scanning microscopy, and also 3D reconstructions. Previous phylactolaemate research has shown that many morphological features such as colony morphology or anatomical details of e.g. the intertentacular membrane, the nervous system or the musculature of the apertural region show variability and a high potential for systematic or phylogenetic comparisons^3,28,47,55^. However, since a solid phylogeny of phylactolaemates has only recently been established^7^, the evolution of morphological characters over the entire range of families and establishment of their plesio- or apomorphic state has not been analysed or addressed. Consequently, this study aims to analyse all available morphological data of phylactolaemate and assess character evolution within Phylactolaemata.

## Materials and methods

Specimen depicted in this study were sampled from various locations over several years listed in Table 1. Generated data and data from literature complement each to obtain a comprehensive character matrix for an ancestral state reconstruction (Table 2).

**Table 1:** Sampling information of all specimen depicted in this study. Key publications concerning colony and zooid morphology are listed for each species.

Family	Genus	Species	Sample location	Sampling date	Key publications
Plumatellidae	*Plumatella*	sp.	Klosterneuburg/Katzelsdorf, Austria (*P. repens*) Bangkok, Thailand (*P. bombayensis*)	2020–2023	Schwaha & Wanninger, 2012 Gruhl, 2007 Braem, 1897
	*Hyalinella*	*punctata*	Klosterneuburg, Austria	2016, 2022	Ambros et al., 2018 Schwaha, 2019a, b
Fredericellidae	*Fredericella*	*sultana*	Klosterneuburg, Austria	July 2022	Schwaha & Wanninger 2012, Gruhl 2007, Braem 1907
	*Internectella*	*bulgarica*	Thailand	2009	Gruncharova, 1971 Wood, 2006 Bibermair & Schwaha, 2021
Hirosellidae	*Hirosella*	*fruticosa*	Hirtzmann Staudamm, Styria, Austria	August 2020	Bibermair & Schwaha, 2023 Toriumi, 1954 Allman, 1844, 1856
Pectinatellidae	*Pectinatella*	*magnifica*	Strandbad Klosterneuburg, Austria	2021	Gawin et al., 2017 Mukai & Oda, 1980
Cristatellidae	*Cristatella*	*mucedo*	Laxenburg Pond, Laxenburg, Austria Lower Austria, Austria	2010, 2020	Gawin et al., 2017 Schwaha, 2019a, b
Lophopodidae	*Lophopus*	*crystallinus*	Petarch, Bulgaria	May-August 2023	Marcus, 1934, Schwaha, 2020
	*Lophopodella*	*carteri*	Ohio, USA	2022	Bibermair et al., 2022 Rogick, 1935, 1937
	*Asajirella*	*gelatinosa*	Sakhon Nakon, Thailand	February 2020	Bibermair et al., 2022 Mukai, 1989
Stephanellidae	*Stephanella*	*hina*	Ware, Massachusetts, U.S.A.	October 2019	Schwaha et al., 2016, 2021 Oka, 1908

**Table 2:** Character matrix with binary and categorical characters. In total 54 characters were analysed. **Legend: 1** character present, **0** character absent, **?** unknown, **/** not applicable, **A** pronounced, **B** present, **C** undifferentiated, **D** basal, **E** tentacle sheath, **F** sphincter/stacked, **G** flap, **H** dome, **I** delicate, **J** absent, **K** 3, **L** 2, **M** 4, **N** crescent, **O** cystigenic, **P** deuteroplasmic, **Q** 1.

			1	2	3	4	5	6	7	8	9	10
			colony morphology									
**Clade/Family**	***Genus***	***species***	**large interzooidal distances** **/serial zooids**	**principial** ** growth** **adherent**	**principial growth** **erect**	**principial ** **growth** **creeping**	**ectocyst** **encrusted**	**ectocyst** **gelatinous**	**ectocyst** **membranous**	**ectocyst** **tubular**	**loose ectocyst**	**ectocyst reduced** **to basal side**
**Plumatellidae**	*Plumatella*	sp.	**1**	**1**	**0**	**0**	**1**	**1**	**0**	**0**	**0**	**0**
	*Hyalinella*	*punctata*	**1**	**1**	**0**	**0**	**1**	**0**	**0**	**0**	**0**	**0**
**Fredericellidae**	*Fredericella*	*sultana*	**1**	**0**	**1**	**0**	**1**	**0**	**0**	**0**	**0**	**0**
**Hirosellidae**	*Hirosella*	*fruticosa*	**1**	**0**	**1**	**0**	**1**	**0**	**0**	**0**	**0**	**0**
**Pectinatellidae**	*Pectinatella*	*magnifica*	**0**	**1**	**0**	**1**	**0**	**1**	**0**	**0**	**?**	**1**
**Cristatellidae**	*Cristatella*	*mucedo*	**0**	**0**	**0**	**1**	**0**	**0**	**1**	**0**	**1**	**1**
**Lophopodidae**	*Lophopus*	*crystallinus*	**0**	**0**	**0**	**1**	**0**	**0**	**1**	**0**	**1**	**1**
	*Lophopodella*	*carteri*	**0**	**0**	**0**	**1**	**0**	**1**	**1**	**0**	**?**	**1**
	*Asajirella*	*gelatinosa*	**0**	**1**	**0**	**0**	**0**	**1**	**0**	**0**	**?**	**1**
**Stephanellidae**	*Stephanella*	*hina*	**1**	**1**	**0**	**0**	**0**	**0**	**0**	**1**	**1**	**0**
			11	12	13	14	15	16	17	18	19	20
			**colony morphology**		**body wall musculature** **(epidermal) (peritoneal)**			**vestibular musculature**				
**Clade/Family**	***Genus***	***species***	**oral budding**	**lateral budding**	**circular**	**longitudinal**	**diagonal**	**circular**	**pronounced** **circular**	**longitudinal**	**pronounced** **longitudinal**	**diaphragmatic** ** sphincter**
**Plumatellidae**	*Plumatella*	sp.	**1**	**0**	**1**	**1**	**0**	**1**	**1**	**1**	**0**	**1**
	*Hyalinella*	*punctata*	**1**	**0**	**1**	**1**	**0**	**1**	**1**	**1**	**0**	**1**
**Fredericellidae**	*Fredericella*	*sultana*	**1**	**0**	**1**	**1**	**0**	**1**	**0**	**1**	**0**	**1**
**Hirosellidae**	*Hirosella*	*fruticosa*	**1**	**0**	**1**	**1**	**0**	**0**	**0**	**1**	**0**	**1**
**Pectinatellidae**	*Pectinatella*	*magnifica*	**1**	**0**	**1**	**1**	**1**	**1**	**0**	**1**	**1**	**1**
**Cristatellidae**	*Cristatella*	*mucedo*	**1**	**0**	**1**	**1**	**0**	**1**	**0**	**1**	**1**	**1**
**Lophopodidae**	*Lophopus*	*crystallinus*	**1**	**0**	**1**	**1**	**1**	**1**	**0**	**1**	**0**	**1**
	*Lophopodella*	*carteri*	**1**	**0**	**1**	**1**	**0**	**1**	**?**	**1**	**?**	**?**
	*Asajirella*	*gelatinosa*	**1**	**0**	**1**	**1**	**0**	**1**	**1**	**1**	**0**	**1**
**Stephanellidae**	*Stephanella*	*hina*	**1**	**1**	**1**	**1**	**0**	**1**	**0**	**1**	**0**	**0**
			21	22	23	24	25	26	27	28	29	30
			**tentacle sheath musculatur (TSM)**				**apertural musculature**			**epistome**		
**Clade/Family**	***Genus***	***species***	**circular**	**abberation** **circular TSM**	**longitudinal**	**abberation** **longitudinal TSM**	**vestibular** **dilatators**	**duplicature** **bands**	**duplicature band attachment**	**Shape** **(1 flap/0 dome)**	**musculature** **basket**	**musculature** **transversal**
**Plumatellidae**	*Plumatella*	sp.	**1**	**D**	**1**	**0**	**1**	**1**	**E**	**1**	**1**	**0**
	*Hyalinella*	*punctata*	**1**	**D**	**1**	**0**	**1**	**1**	**E**	**1**	**1**	**1**
**Fredericellidae**	*Fredericella*	*sultana*	**1**	**A**	**1**	**0**	**1**	**1**	**E**	**1**	**1**	**0**
**Hirosellidae**	*Hirosella*	*fruticosa*	**1**	**0**	**1**	**0**	**1**	**1**	**F**	**1**	**1**	**1**
**Pectinatellidae**	*Pectinatella*	*magnifica*	**1**	**0**	**1**	**0**	**1**	**1**	**E**	**1**	**0**	**1**
**Cristatellidae**	*Cristatella*	*mucedo*	**1**	**0**	**1**	**1**	**1**	**1**	**E**	**1**	**1**	**0**
**Lophopodidae**	*Lophopus*	*crystallinus*	**0**	**0**	**1**	**0**	**1**	**1**	**E**	**0**	**1**	**1**
	*Lophopodella*	*carteri*	**0**	**0**	**1**	**0**	**1**	**1**	**E**	**0**	**1**	**1**
	*Asajirella*	*gelatinosa*	**0**	**0**	**1**	**0**	**1**	**1**	**E**	**0**	**1**	**1**
**Stephanellidae**	*Stephanella*	*hina*	**1**	**0**	**1**	**1**	**1**	**1**	**E**	**1**	**1**	**1**
			31	32	33	34	35	36	37	38	39	40
			**lophophore muscles**			**abfrontal tentacle muscles**				**frontal tentacle muscles**		
**Clade/Family**	***Genus***	***species***	**lophophoral** ** arms** **muscles**	**circum-pharyngeal** **muscles**	**ring canal ** **muscles**	**median** ** bands**	**stacked**	**gap base/** **tent. muscle**	**proximal** ** process**	**median** ** bands**	**lateral** ** connections**	**rootlets**
**Plumatellidae**	*Plumatella*	sp.	**B**	**1**	**1**	**1**	**0**	**0**	**0**	**0**	**1**	**K**
	*Hyalinella*	*punctata*	**I**	**1**	**1**	**1**	**0**	**0**	**0**	**0**	**1**	**L**
**Fredericellidae**	*Fredericella*	*sultana*	**J**	**1**	**0**	**1**	**0**	**1**	**0**	**0**	**1**	**K**
**Hirosellidae**	*Hirosella*	*fruticosa*	**I**	**1**	**0**	**1**	**0**	**1**	**0**	**0**	**1**	**K**
**Pectinatellidae**	*Pectinatella*	*magnifica*	**A**	**1**	**1**	**1**	**0**	**0**	**0**	**1**	**1**	/
**Cristatellidae**	*Cristatella*	*mucedo*	**A**	**1**	**1**	**1**	**0**	**0**	**0**	**0**	**1**	**K**
**Lophopodidae**	*Lophopus*	*crystallinus*	**B**	**1**	**?**	**1**	**0**	**0**	**1**	**0**	**1**	**L**
	*Lophopodella*	*carteri*	**A**	**0**	**?**	**1**	**0**	**0**	**1**	**0**	**0**	**K**
	*Asajirella*	*gelatinosa*	**A**	**1**	**?**	**1**	**0**	**0**	**1**	**0**	**1**	**M**
**Stephanellidae**	*Stephanella*	*hina*	**J**	**0**	**0**	**0**	**1**	**1**	**1**	**0**	**0**	**L**
			41	42	43	44	45	46	47	48	49	50
			**intertentacular** ** membrane**		**CNS**		**statoblasts**					
**Clade/Family**	***Genus***	***species***	**oral gap**	**lamella**	**ganglionic** **horns**	**enlargment lumen/** **epistomial horns**	**Sessoblast**	**Development**	**Floatoblast**	**Spinoblast**	**Leptoblast**	**Piptoblast**
**Plumatellidae**	*Plumatella*	sp.	**1**	**0**	**B**	**0**	**1**	**O**	**1**	**0**	**1**	**0**
	*Hyalinella*	*punctata*	**?**	**?**	**B**	**0**	**0**	**?**	**1**	**0**	**0**	**0**
**Fredericellidae**	*Fredericella*	*sultana*	**1**	**0**	**J**	**0**	**1**	**O**	**0**	**0**	**0**	**1**
**Hirosellidae**	*Hirosella*	*fruticosa*	**1**	**1**	**B**	**0**	**1**	**O**	**1**	**0**	**0**	**0**
**Pectinatellidae**	*Pectinatella*	*magnifica*	**0**	**1**	**B**	**0**	**0**	**?**	**0**	**1**	**0**	**0**
**Cristatellidae**	*Cristatella*	*mucedo*	**0**	**1**	**B**	**0**	**0**	**?**	**0**	**1**	**0**	**0**
**Lophopodidae**	*Lophopus*	*crystallinus*	**0**	**0**	**B**	**1**	**0**	**?**	**1**	**0**	**0**	**0**
	*Lophopodella*	*carteri*	**?**	**?**	**B**	**1**	**0**	**?**	**0**	**1**	**0**	**0**
	*Asajirella*	*gelatinosa*	**0**	**1**	**B**	**1**	**0**	**?**	**0**	**1**	**0**	**0**
**Stephanellidae**	*Stephanella*	*hina*	**0**	**1**	**N**	**0**	**1**	**P**	**1**	**0**	**0**	**0**
			51	52	53	54						
			**larvae**									
**Clade/Family**	***Genus***	***species***	**ciliated ** **mantle larvae**	**polypides**	**spot - like** **placenta**	**ring - like** **placenta**						
**Plumatellidae**	*Plumatella*	sp.	**1**	**L**	**0**	**1**						
	*Hyalinella*	*punctata*	**1**	**L**	**0**	**1**						
**Fredericellidae**	*Fredericella*	*sultana*	**1**	**Q**	**1**	**0**						
**Hirosellidae**	*Hirosella*	*fruticosa*	**1**	**Q**	**?**	**?**						
**Pectinatellidae**	*Pectinatella*	*magnifica*	**1**	**M**	**1**	**0**						
**Cristatellidae**	*Cristatella*	*mucedo*	**1**	**M**	**1**	**0**						
**Lophopodidae**	*Lophopus*	*crystallinus*	**1**	**L**	**?**	**?**						
	*Lophopodella*	*carteri*	**1**	**L**	**?**	**?**						
	*Asajirella*	*gelatinosa*	**1**	**L**	**?**	**?**						
**Stephanellidae**	*Stephanella*	*hina*	**?**	**?**	**?**	**?**						

Specimens were either fixed in 4% paraformaldehyde in 0.1 M phosphate buffer (PB) for fluorescence microscopy, or in 2% glutaraldehyde in 0.01 M PB for histological analyses (for further details see Bibermair et al., Ruthensteiner^43,56,57^. Stereomicroscopic images were taken on Nikon SMZ 25 Microscope (Nikon, Tokyo, Japan) with a Nikon Ds-Ri2 camera. Histological sections were produced using a HistoJumbo diamond knife (Diatome, Nidau, Switzerland) on a Leica Ultracut 6 microtome (Leica Microsystems, Wetzlar Germany), and photographed with a Nikon Ds-Ri2 on a Nikon NiU compound microscope. Image stack processing and 3D reconstruction was conducted with Fiji^58^and Amira (v. 2024; Thermo Fischer) software. For details see e.g. Bibermair et al.^43^.

**Fig. 1:**
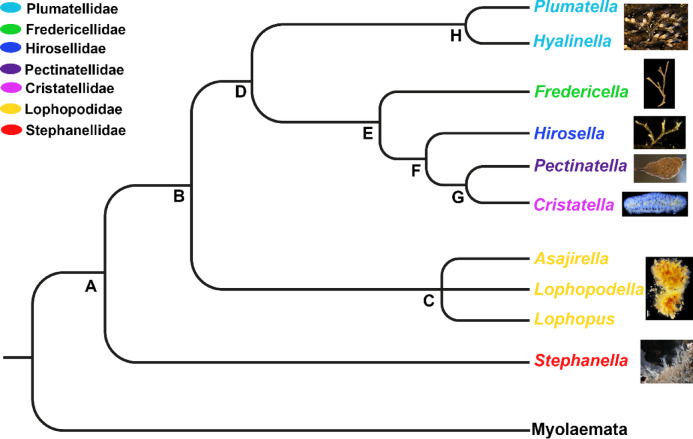
Phylogeny of Phylactolaemata. Simplified and redrawn form Saadi et al.^7^. Phylactolaemata as the sistergroup of Myolaemata. The phylogenetic tree is at genus level; most families are monotypic. Genera of the same family are indicated by colour.

For the ancestral state reconstruction, a character matrix (Table 2) was assembled, based on data of this study as well as information from early and recent literature. The character matrix includes categorical and binary characters that were transferred into a nexus file together with a phylogeny modified from most recent molecular analysis of phylactolaemate systematics^7^. Final analysis was carried out with Mesquite (v. 3.81)^59^, following the procedure of previous studies (Schwaha et al.^60^; “Trace Character History” and “Likelihood Ancestral State” option). Mesquite Mk1 equal-rates model was used for ancestral state reconstruction; this model assumes equal transition probabilities among all states, a limitation we acknowledge given the sparse character dataset. The results of the ancestral state reconstruction were exported from Mesquite for each character individually (Fig. S1) and will be discussed via the phylogeny redrawn and simplified from Saadi et al.^7^ (Fig. 1).

## Results and discussion

### Colony morphology and ectocyst

**Fig. 2:**
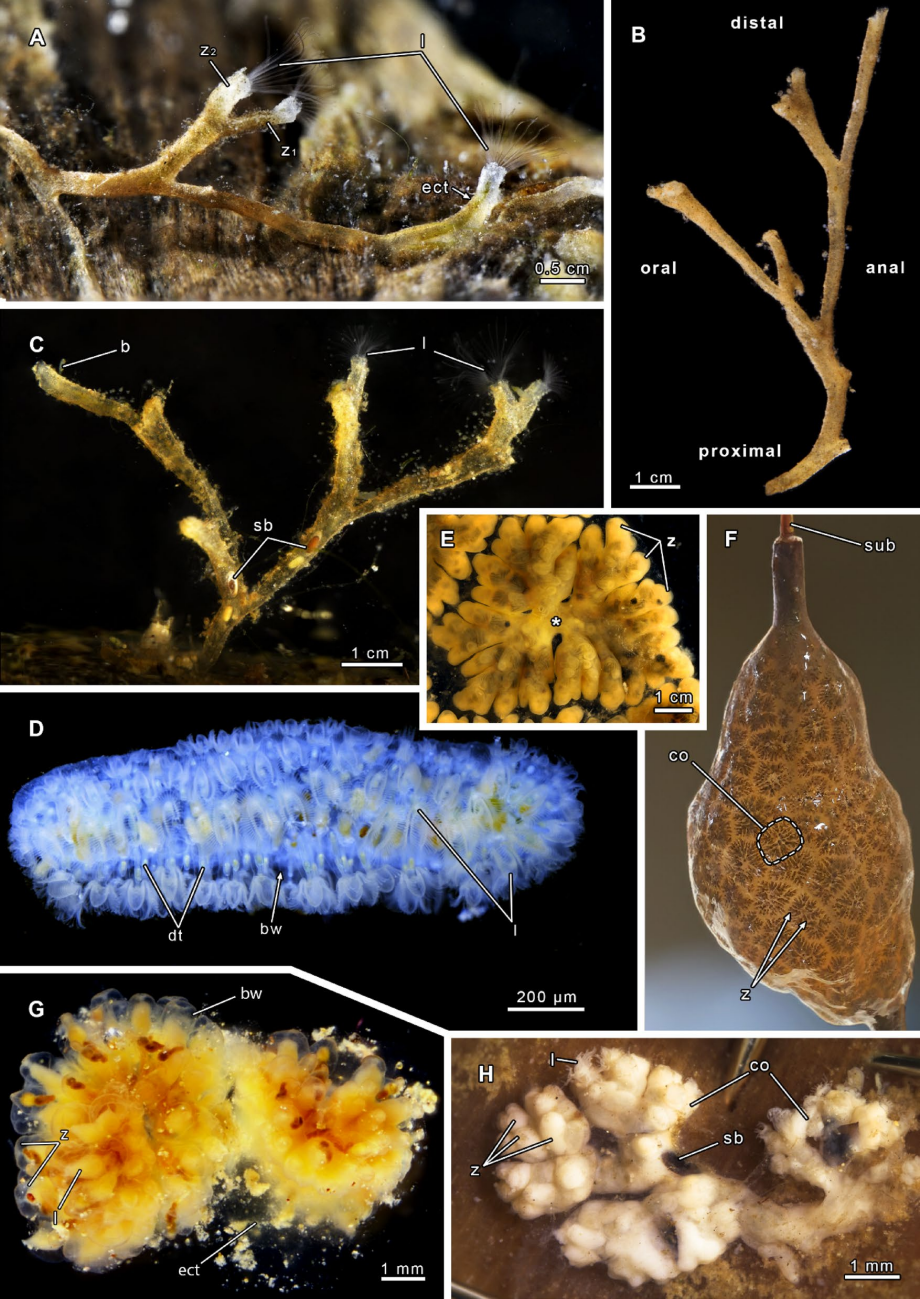
Colonies of phylactolaemate families. **(A-B)** *Fredericella sultana* (Fredericellidae). **(A)** Living specimen with circular lophophores. **(B)** Colonies are only attached with small, repent, proximal branches whereas most of the colony is erect. **(C)** *Hirosella fruticosa* (Hirosellidae). The colony is mostly erect with only proximal parts attached to the substrate. **(D)** Caterpillar-like colony of *Cristatella mucedo* (Cristatellidae). **(E)** Single, rosette-shaped colony of *Pectinatella magnifica* (Pectinatellidae), view from below. **(F)** Compound colony of *P. magnifica*. **(G)** *Asajirella gelatinosa* (Lophopodidae) with dense, circular colonies. **(H)** *Lophopus crystallinus* (Lophopodidae) with lobes of clustered zooids. Abbreviations: **b** bud, **bw** body wall, **co** colony, **dt** digestive tract, **ect** ectocyst, **l** lophophore, **sb** statoblast, **sub** substrate, **z** zooid; **z**_**1**_ maternal zooid, **z**_**2**_ daughter zooid; asterisk: colony origin.

**Fig. 3:**
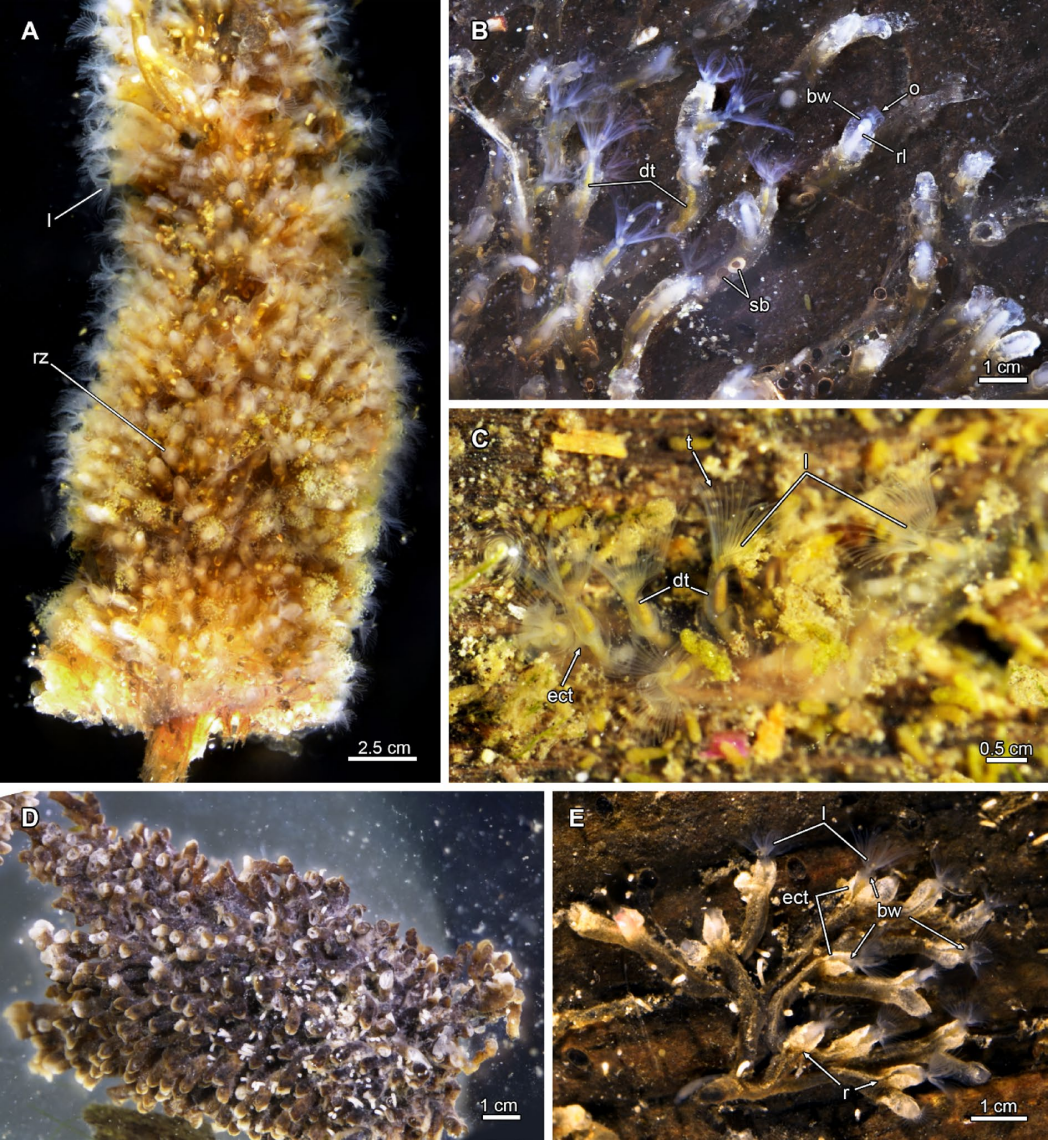
Living colonies of Plumatellidae. **(A)** Dense colony of *Plumatella repens*. **(B)** *P. repens* with less dense colony. **(C)** *Hyalinella punctata* with irregular colony growth pattern. The zooids are entirely transparent. **(D)** A dense colony of *P. bombayensis* showing dark ectocyst with bright tips. **(E)** Young colony of *P.* cf. *casmiana*. The distal ectocyst frequently shows a non-encrusted area and a raphe. Abbreviations: **bw** body wall, **dt** digestive tract, **ect** ectocyst, **l** lophophore, **o** orifice, **r** raphe, **rl** retracted lophophore, **rz** retracted zooid, **sb** statoblast, **t** tentacles.

The colonies of phylactolaemates form a variety of shapes, ranging from adherent to erect, serial forms to creeping, caterpillar-like colonies (Figs. 2 and 3^28^). Differences are found in the serial arrangement of zooids, which are either wider apart and hence have large interzooidal distances like in Stephanellidae, Hirosellidae (Fig. 2C), Fredericellidae (Fig. 2A, B) and Plumatellidae (Fig. 2), or are more clustered with short interzooidal distances as found in Lophopodidae (Fig. 2G, H), Cristatellidae (Fig. 2D) and Pectinatellidae (Fig. 2E, F). Clustered arrangements of zooids often result in a fan-shaped colony in lophopodids^11,39,61^, and *Pectinatella magnifica* (Fig. 2E^62^). The most aberrant colony shape is found in *Cristatella* with its unique caterpillar-like colonies (Fig. 2D) that show the original serial arrangement of zooids by the alternating bud production on the colony margin^12^. *Cristatella* and most lophopodids are capable of locomotion by creeping over their entire life, whereas it is restricted to early germinating zooids in *Pectinatella*. Ancestral state analyses favour a serial arrangement of zooids as the ancestral condition (Figs. 1A; S1). Hence, the short interzooidal distances of zooids, as well as the creeping locomotion, evolved presumably twice, once in lophopodids and in the *Pectinatella*/*Cristatella* clade (Figs. 1C, G; S1).

Phylactolaemate colonies can be addressed as adherent, erect or creeping; and referring to their ectocyst as tubular, encrusting/chitinous, gelatinous and membranous^11^. In *Stephanella* the colony has erect zooids that are interconnected via a proximal thin part, which is adherent to the substrate^35,37,38^. The basal, adherent part of plumatellids is much larger and houses most of the polypide, whereas the erect portion bending frontally is short. The serial branching colonies of plumatellids have distant and regular branching (Fig. 3B, D, E), to an irregular branching pattern, often also leading to a dense aggregation of zooids with short interzooidal distances (Fig. 3A, D^11,12^). Some plumatellids also form erect zooids when space on the substrate is limited and colonies are dense. Fredericellidae and Hirosellidae form erect colonies in which all branches are suspended in the water and only the proximal area is attached to the substrate (Fig. 2A, B, C^12–14,49,63^). Erect colonies evolved presumably at the base of the *Fredericella*/HCP clade (Fig. 1E), and got lost within *Cristatella* and *Pectinatella*, which parallels with the independent evolution of reduced interzooidal distances (Figs. 1G; S1).

Lophophodidae, Cristatellidae and Pectinatellidae are generally regarded as gelatinous representatives, while Plumatellidae, Fredericellidae and Hirosellidae are addressed as encrusted/chitinous phylactolaemates. The terms gelatinous and encrusted/chitinous indicate an outdated phylogenetic classification used for early systematic considerations^14,15,17,30,53^. Families with gelatinous representatives secrete a thick and transparent ectocyst that is usually restricted to the basal side of zooids, and in *Pectinatella* (Pectinatellidae) and *Asajirella* (Lophopodidae) gradually forms the substrate to grow on^28,41,64^. In contrast to *Pectinatella* and *Asajirella*, the ectocyst in *Cristatella* is a thin layer between the creeping sole and substrate. This condition, referred to as membranous, is also found in the lophopodid genera *Lophopus* and *Lophopodella*. Consequently, Pectinatellidae, Cristatellidae, Lophopodidae are largely referred to as gelatinous, although this is not the case for the lophopodid *Lophopus* and Cristatellidae. In addition, the ectocyst in Pectinatellidae, Cristatellidae and Lophopodidae is restricted to the basal side of the colony and in Cristatellidae and the lophopodids *Lophopus* and *Lophopodella* is membranous, contrasting the thick ectocysts of the lophopodid *Asajirella* and Pectinatellidae. Families with large interzooidal distances secrete a sticky ectocyst which agglutinates particles. Because of a distinct brown coloration of such cystids they are usually referred to as chitinous, although the exact composition is unknown. Detritus particles are also frequently encrusted into the cystid of such species, which gives them a ‘sandy’ appearance of the cystid (e.g. Figures 2A and B and 3D and E). This is also particularly evident in fredericellids, which naturally are covered in a high density of particles, whereas stay transparent and clear under laboratory conditions (when cultured upside down). *Hirosella* shows a high cystid similarity to fredericellids^49^and most likely would act similarly under laboratory conditions. The plumatellid genera *Hyalinella*,* Gelatinella* and *Rumarcanella* show a transparent, and the former two also gelatinous, ectocyst (Fig. 3B, C^20,65,66^), which, however, differs from gelatinous representatives by covering the entire zooid and not just the basal side. The latter condition is presumably derived and evolved independently in *Cristatella*/*Pectinatella* and lophopodids (Fig. S1). Stephanellidae have a similar transparent, but more tube-like ectocyst secretion^35,38^. Contrary to all other phylactolaemates this type of ectocyst is largely unattached to the epidermis and hence not a true cuticle/ectocyst. Resulting from ancestral state analysis, this condition has an increased probability to be ancestral, although the condition of some lophopodids and *Pectinatella* remains unknown. Further the ancestral state reconstruction recognises the encrusting ectocyst as derived character, which has evolved once (Figs. 1D; S1) and is lost in *Pectinatella*/*Cristatella* (Figs. 1G; S1). The origin of gelatinous, membranous ectocysts can’t be answered with current data (Fig. S1).

## Body wall musculature

**Fig. 4:**
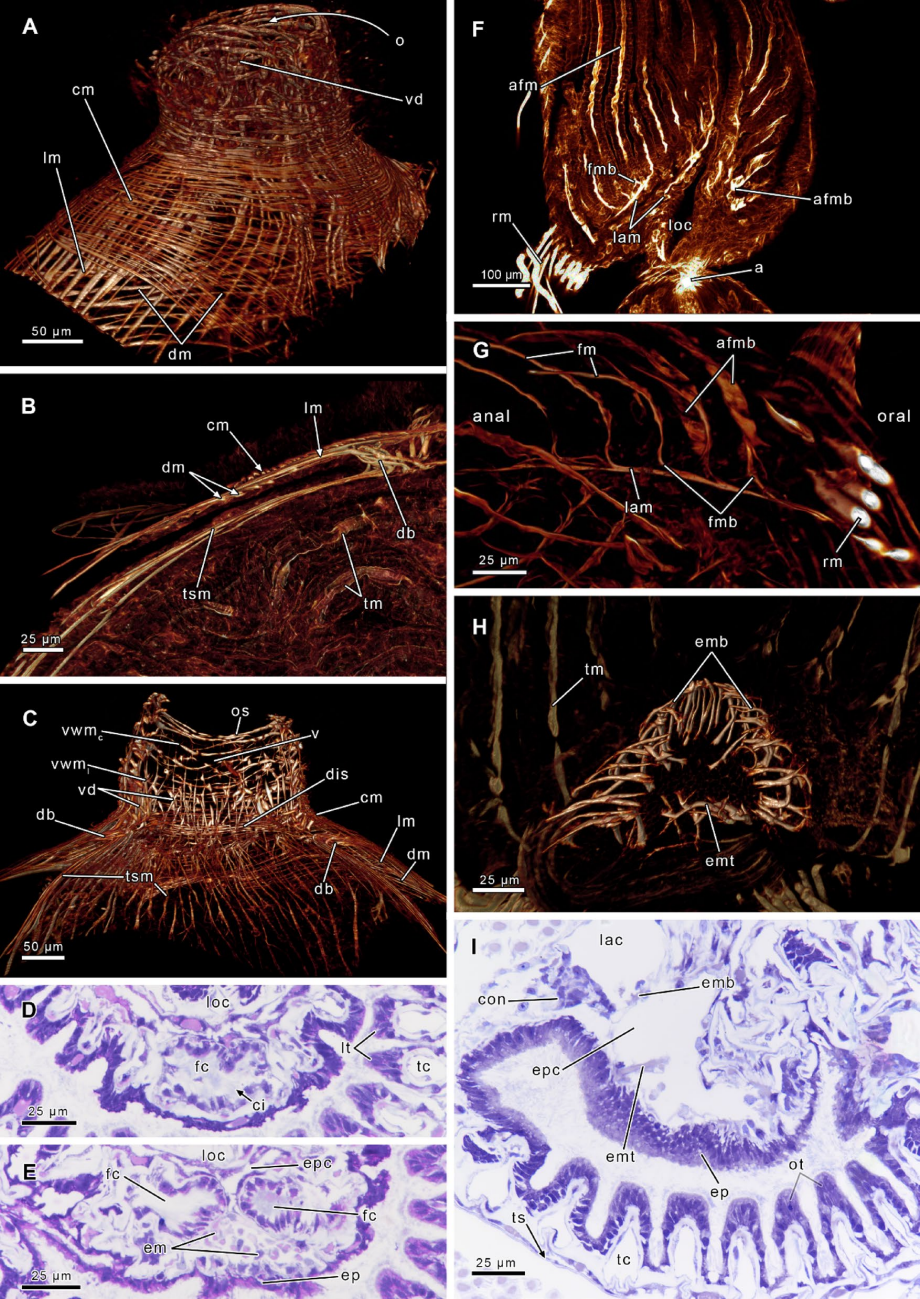
Morphological details of *Lophopus crystallinus* based on phalloidin staining, confocal microscopy and histological sections. **(A)** Distal region of the body wall musculature shows three layers of body wall muscles in the area proximal of the vestibulum. **(B)** Body wall and tentacle sheath: circular (outer), longitudinal (middle), and diagonal (inner) body wall muscles. **(C)** Apertural region and tentacle sheath: longitudinal and circular vestibular wall muscles are separated from the tentacle sheath via a diaphragmatic sphincter. **(D)** Histological section through merging shanks of the forked canal. **(E)** Section proximal of (D). The canal is paired and passes over the epistome coelom. **(F)** Lophophoral arm musculature with only few muscle strands. **(G)** Frontal tentacle muscles connected to lophophoral arm muscles via 1–2 rootlets. **(H)** Epistome musculature consisting of a muscular basked and central transversal muscles. **(I)** Histological cross section through the epistome with a spacious coelom through which thick epistome muscle traverse. Abbreviations: **a** anus, **afmb** abfrontal tentacle muscle base, **ci** cilia, **cm** circular body wall musculature, **con** circumoral nerve ring, **db** duplicature bands, **dm** diagonal body wall musculature, **dis** diaphragmatic sphincter muscle, **em** epistome muscles, **emb** epistome muscle basket, **emt** traversing epistome musculature, **ep** epistome, **epc** epistome coelom, **fc** forked canal, **fm** frontal tentacle muscle, **fmb** frontal tentacle muscle base, **lam** lophophoral arm musculature, **lac** lophophoral arm coelom, **lm** longitudinal body wall musculature, **loc** lophophoral concavity, **lt** lateral tentacles, **o** orifice, **os** orificial sphincter, **ot** oral tentacles, **rm** retractor muscle, **tc** tentacle coelom, **tm** tentacle muscle, **ts** tentacle sheath, **tsm** tentacle sheath musculature, **v** vestibulum, **vd** vestibular dilatators, **vwm**_**c/l**_ circular/longitudinal vestibular wall musculature.

The body wall consists of an outer epidermal layer and adjacent, inner peritoneal layer^5,23^. This condition is considered ancestral for bryozoans and is only present in phylactolaemates^5,67^. Myolaemate bryozoans have strongly modified this ancestral condition with either separation or reduction of the peritoneal layer^3,5,28,68^. The body wall of phylactolaemates includes an orthogonal grid of body wall muscles (Fig. 4A, B, C). The circular body wall muscles are associated with the outer epidermis, while the longitudinal muscles with the inner peritoneal layer (Fig. 4B). Consequently, the circular muscles can be referred to as subepidermal and the longitudinal muscles as subperitoneal^5,23,39^. A third layer of diagonal muscles is present in the distal part of the body wall of *Pectinatella magnifica*^47^and *Lophopus crystallinus* (Fig. 4A, B, C^39^). The diagonal layer of body wall muscles is present as innermost layer of the body wall and is therefore of peritoneal origin (Fig. 4B). Ultrastructural investigations of this area are necessary to confirm this assumption. The third layer of body wall muscles has not been reported in other lophopodids and has likely evolved independently in *Pectinatella* and *Lophopus* (Fig. S1^43^).

## Vestibular wall musculature

At the orifice, the body wall invaginates to form the vestibular wall. In retracted condition, the vestibular wall is compressed and forms a small cavity, the vestibulum, in all bryozoans^28^. Vestibular wall muscles follow the orthogonal body wall musculature in phylactolaemates but show some differences in the extent of circular or longitudinal muscles among different representatives. *Stephanella*, *Lophopus* and *Lophopodella* show no difference in the arrangement of body wall muscles, with an equal grid of circular and longitudinal muscles (Fig. 4C^35,43^). In the lophopodid genus *Asajirella*, the circular vestibular wall muscles are more prominent than the longitudinal ones.

Fredericellidae, the sister taxon to the *Hirosella/Pectinatella*/*Cristatella* clade, resembles the condition as found in stephanellids^49^. Pectinatellidae and Cristatellidae have both vestibular muscle sets with stronger longitudinal muscles^47,69^, while the sister taxon, *Hirosella*, lacks any circular muscles^49^.

Plumatellids show a condition opposite of *Cristatella*/*Pectinatella* and show stronger circular than the longitudinal vestibular wall muscles^15,23,29^. Concluding from all data, the ancestral state of vestibular muscles reflects the arrangement of body wall muscles, with individual specializations that evolved within the clades independently (Figs. 1A, G, H; S1).

## Tentacle sheath musculature

Proximally, the vestibular wall continues into the tentacle sheath (retracted condition). At its transition, a diaphragmatic sphincter muscle separates their cavities (vestibulum, atrium) in retracted zooids of almost all bryozoans (Fig. S1). Except for *Stephanella hina*^35^, all phylactolaemates possess densely aggregated circular muscles at the diaphragm (e.g. Figure 4C). In ancestral condition, the tentacle sheath is reminiscent of the orthogonal body wall muscles and has longitudinal and circular muscles, which is observed in most phylactolaemates (Fig. S1). Similar to the vestibular wall muscles, several phylactolaemate families have either stronger longitudinal or circular tentacle sheath muscles, or absence of either. Stephanellids have both, with prominent longitudinal bundles^35^. Lophopodids lack circular muscles in the tentacle sheath but show proximally and distally bifurcating muscle bundles (Fig. 4C^39,40,43^). *Pectinatella*,* Cristatella* and *Hirosella* possess both tentacle sheath muscle layers^47,69^. Fredericellidae is the only family with stronger circular muscles in the tentacle sheath^49^. Plumatellids have mostly longitudinal fibres, and circular muscles are restricted to the proximal region of the tentacle sheath^29,47^. Noteworthy, the arrangement of the tentacle sheath muscles does not reflect that of the vestibular wall muscles. Concluding from the current phylogeny, an orthogonal mesh of circular and longitudinal musculature seems ancestral, with circular ones lost in lophopodids and restricted in plumatellids (Figs. 1C, H; S1).

### Apertural muscles

The vestibular wall is connected to the body wall by short muscle fibres (vestibular dilatators) and the tentacle sheath by duplicature bands (peritoneal strands with longitudinal muscles). Both of these muscles extend radially towards the body wall, with the vestibular dilatators being more numerous, whereas duplicature bands are present in lesser numbers^29,35,40,43,47,69^. While the vestibular dilatators are very delicate and thin muscle bundles, the duplicature bands are comparatively thick tissue strands that include several muscle fibres (Fig. 4B, C). Duplicature bands generally originate from the distal region of the tentacle sheath, with their muscle fibres being extension from the fibres of the longitudinal tentacle sheath muscles (Fig. 4B, C). In fredericellids, these muscles can be continuous with body wall muscles^49^. Duplicature bands emanate proximally of the diaphragmatic sphincter (Fig. 4B, C^28,43^), but originate from the sphincter itself in *Hirosella fruticosa* (Fig. S1^49^). With the exception of this deviation in *H. fruticosa* apertural muscles are identical among phylactolaemates and do not appear to have any phylogenetic importance in regard of character evolution.

## Lophophore and lophophoral base

The lophophoral base is the most complex region of a zooid, and includes the central nervous system, the epistome and the lophophore in general.

## Epistome

**Fig. 5:**
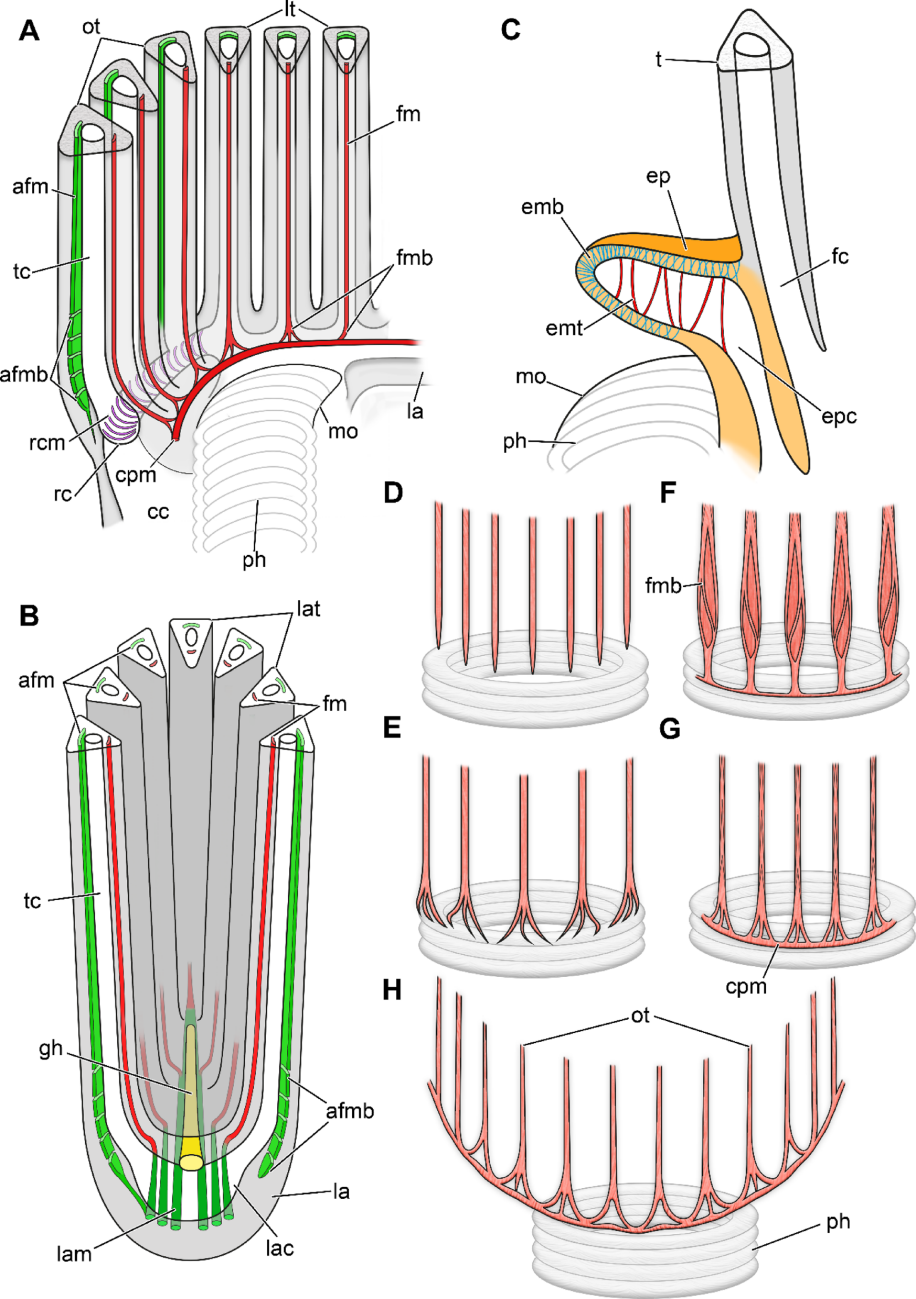
Schematic drawings of different regions of the lophophoral base and frontal tentacle muscles. **(A)** Section through the lophophoral base. Ring canal muscles traverse the proximal border of the oral ring canal (Cristatellidae, Pectinatellidae, Plumatellidae, Lophopodidae). A circum-pharyngeal muscle ring or continuous connection between frontal tentacle muscles exists in most phylactolaemates except *Stephanella* and *Lophopodella*. **(B)** Section through a lophophoral arm. Frontal tentacle muscles are connected to the lophophoral arm muscles, abfrontal tentacle muscles only in Pectinatellidae, Cristatellidae, Lophopodidae. **(C)** Section through the epistome. Two sets of muscles are present in the epistome: an epistomial muscle basket (blue lines; all phylactolaemates except Pectinatellidae) and muscles traversing the epistome coelom (red lines, Stephanellidae, Lophopodidae, Pectinatellidae, Hirosellidae and the plumatellid *Hyalinella*). **(D-H)** Frontal tentacle muscles of oral tentacles of *Stephanella* (D), *Lophopodella* (E), *Pectinatella* (F), *Cristatella* (G) and *Asajirella*, *Lophopus*, *Fredericella*, *Hirosella* and Plumatellidae (H). Abbreviations:, a**fm** frontal tentacle muscle, **afmb** abfrontal tentacle muscle (base), **cc** coelomic cavity, **cpm** circum pharyngeal muscle ring, **emb** epistomial muscle basket, **emt** transversal epistome muscles, **ep** epistome, **epc** epistome coelom, **fc** forked canal,, **fm** frontal tentacle muscle, **fmb** frontal tentacle muscle base, **gh** ganglionic horns, **la** lophophora arm, **lac** lophophoral arm coelom, **lam** lophophoral arms musculature, **lat** lophophoral arm tentacles, **lt** lateral tentacles, **mo** mouth opening, **ot** oral tentacles, **ph** pharynx, **rc** ring canal, **rcm** ring canal muscles, **tc** tentacle coelom.

**Fig. 6:**
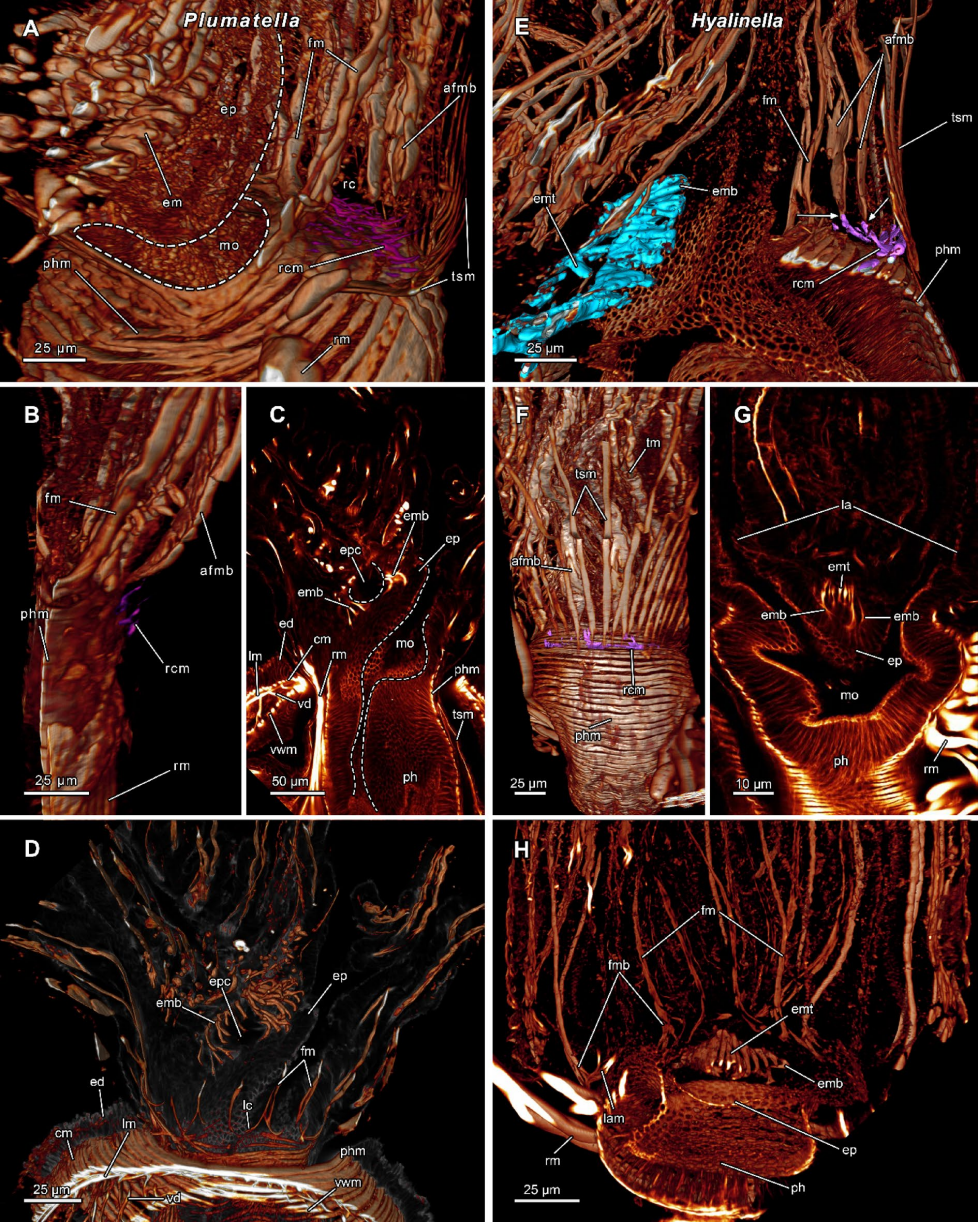
Lophophoral base musculature of the plumatellids *Plumatella* cf. *repens* (A-D) and *Hyalinella punctata* (E-H). **(A)** Lateral view of the lophophoral base showing ring canal muscles. **(B)** Ring canal muscles proximal of the tentacle muscles. **(C)** Sagittal section of the epistome coelom lined by epistome muscles forming a basket. (**D**) Protruded polypide showing oral tentacles with connected frontal tentacle muscles. **(E)** Lateral view of the lophophoral base showing the epistome with a muscle basket. The abfrontal tentacle muscle bases have proximal projections seemingly confluent with the ring canal muscles. **(F)** Frontal view with thick longitudinal muscles of the tentacle sheath. **(G-H)** Cross section of the central epistome. Beside the epistome muscle basket, muscle fibres also traverse the epistome coelom. **(H)** Frontal tentacle muscles of the lateral tentacles are rooted in the lophophoral arms musculature. Abbreviations: **afmb** abfrontal tentacle muscle base, **cm** circular body wall musculature, **ed** epidermis, **em** epistome muscles, **emb** epistome muscle basket, **emt** traversing epistome musculature, **ep** epistome, **epc** epistome coelom, **fm** frontal tentacle muscle, **fmb** frontal tentacle muscle base, **la(m)** lophophoral arm (musculature), **lc** lateral connections, **lm** longitudinal body wall musculature, **mo** mouth opening, **ph** pharynx, **phm** pharynx musculature, **rc** ring canal, **rcm** ring canal muscle, **rm** retractor muscle, **tm** tentacle muscle, **tsm** tentacle sheath musculature, **vd** vestibular dilatators, **vwm** vestibular wall musculature.

The phylactolaemate epistome is a heavily ciliated, flap- (or in lophopodids dome-) shaped organ protruding laterally in oral direction over the mouth opening (Figs. 4I, 5C and 6A, C and D; Stephanellidae^35^; Lophopodidae^43,70^; Cristatellidae^69,71,72^; Pectinatellidae^47^; Hirosellidae^49^; Fredericellidae^29^; Plumatellidae^23^). Its epidermal layer is thick and comprises high-prismatic cells (Fig. 4I^23,35^). Centrally, the epistome contains a coelomic extension from the main coelomic cavity, the epistome coelom (Figs. 4 and 5C , D^23^). The peritoneal cells of the epistome coelom can be myoepithelial. Phylactolaemates show two different sets of muscles: muscles that are restricted to the peritoneal lining of the epistome and thereby form an epistomial muscle basket (Figs. 4H, 5C and 6E, G and H). These are present in all investigated phylactolaemates except pectinatellids (see citations above). The second set features thick muscles traversing the epistome coelom, the transversal epistome musculature (Fig. 5C). These are the only muscles in pectinatellids^47^and are also present in lophopodids (Fig. 4H, I^43^), hirosellids^49^ the plumatellid genus *Hyalinella* (Figs. 6E, G, H^47^) and also stephanellids^35^. Hence the latter four include both sets of epistome muscles. In addition to the muscle basket and the transversing epistome muscles, phylactolaemates generally seem to have proximal epistome musculature, but it has probably been overlooked frequently and needs further attention. The distribution of the two different epistome muscle sets among phylactolaemates indicates that both were present in the last common ancestor of phylactolaemates, with several losses of the transversal epistome muscles and a single loss of the muscular basket (Fig. S1).

### Forked Canal

The tentacles above the epistome, in the lophophoral concavity, are supplied by a separate, distinctly ciliated coelomic canal, the so-called forked canal (Fig. 4D and E C^15,23,71,73^). The forked canal commences on both lateral sides above the cerebral ganglion, arches medially above the epistome coelom and supplies two to five tentacles of the lophophoral concavity^23,68^. With the exception of *Stephanella hina* the two lateral coelomic protrusions fuse above the epistome (Fig. 4D^35^). *Cristatella mucedo* can show a large widening of the forked canal at its median fusion, termed excretory bladder (^11,71^). This bladder is apomorphic to *Cristatella mucedo*, but its excretory function is unclear.

### Lophophoral arm musculature

**Fig. 7:**
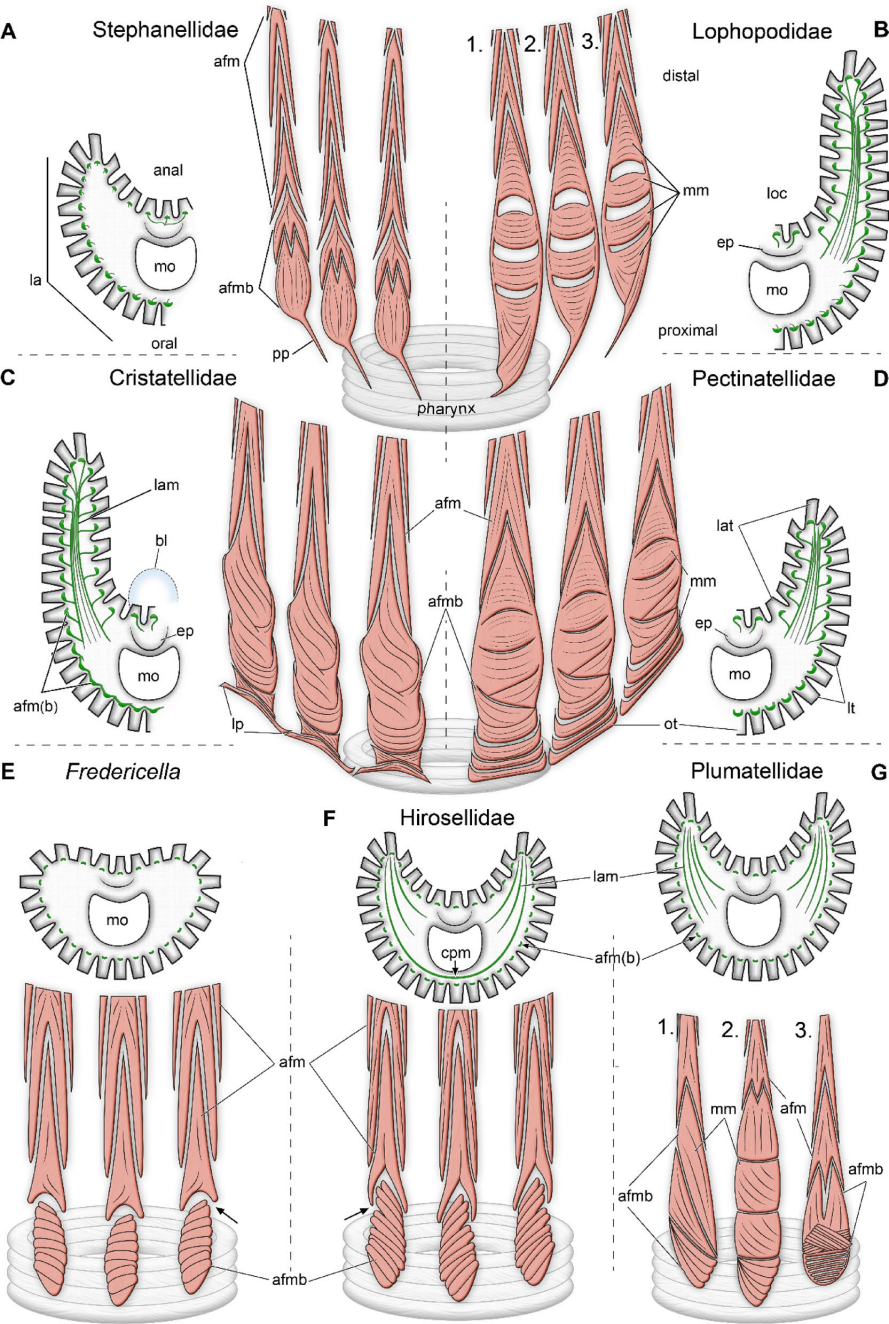
Schematic overview of abfrontal tentacle muscle bases and lophophoral arms muscles of phylactolaemates. **(A)** Stephanellidae. No distinct lophophoral arm muscles; abfrontal bases with proximal processes stacked longitudinal muscles. **(B)** Lophopodidae: *Asajirella* (1), *Lophopodella* (2) and *Lophopus* (3). Abfrontal bases with proximal processes and crossing muscle bundle that conjoin laterally. Median muscle bands traverse between the lateral lining. Lophophoral arms with prominent lophophoral arms muscles and connected abfrontal bases. **(C)** Cristatellidae. Broad abfrontal muscle bases with lateral extension in proximal region. Long lophophoral arms with muscles like lophopodids. **(D)** Pectinatellidae. Broad abfrontal muscle bases and numerous median muscle bands. Proximal bands project in anal direction; lophophoral arms muscles like *Cristatella*. **(E)** Fredericellids. No lophophoral arms musculature. Compact abfrontal bases, with transversally orientated muscle bundles and a gap between to the tentacle muscle. **(F)** Hirosellidae. Tentacle muscles similar to Fredericellidae. Lophophoral arms musculature without connection to the abfrontal bases and a continuous muscular connection around the pharynx. **(G)** Plumatellidae: *P. repens* (1), *P. casmiana* (2), *Hyalinella punctata* (3). *Plumatella* with distinct sets of traversing muscle bundles. *Hyalinella* with crossing muscle bundles and longitudinal muscle on top. Lophophoral arm musculature without connection to abfrontal tentacle muscles. Abbreviations: **afm** abfrontal tentacle muscles, **afmb** abfrontal tentacle muscle bases, **bl** bladder, **cpm** circum pharyngeal muscle ring, **ep** epistome, lam lophophoral arm muscles, **lat** lophophoral arm tentacles, **loc** lophophoral concavity, **lp**, lateral projections, **lt** lateral tentacles, **mm** median muscle bands, mm mouth opening, **ot** oral tentacles, **pp** proximal processes.

**Fig. 8:**
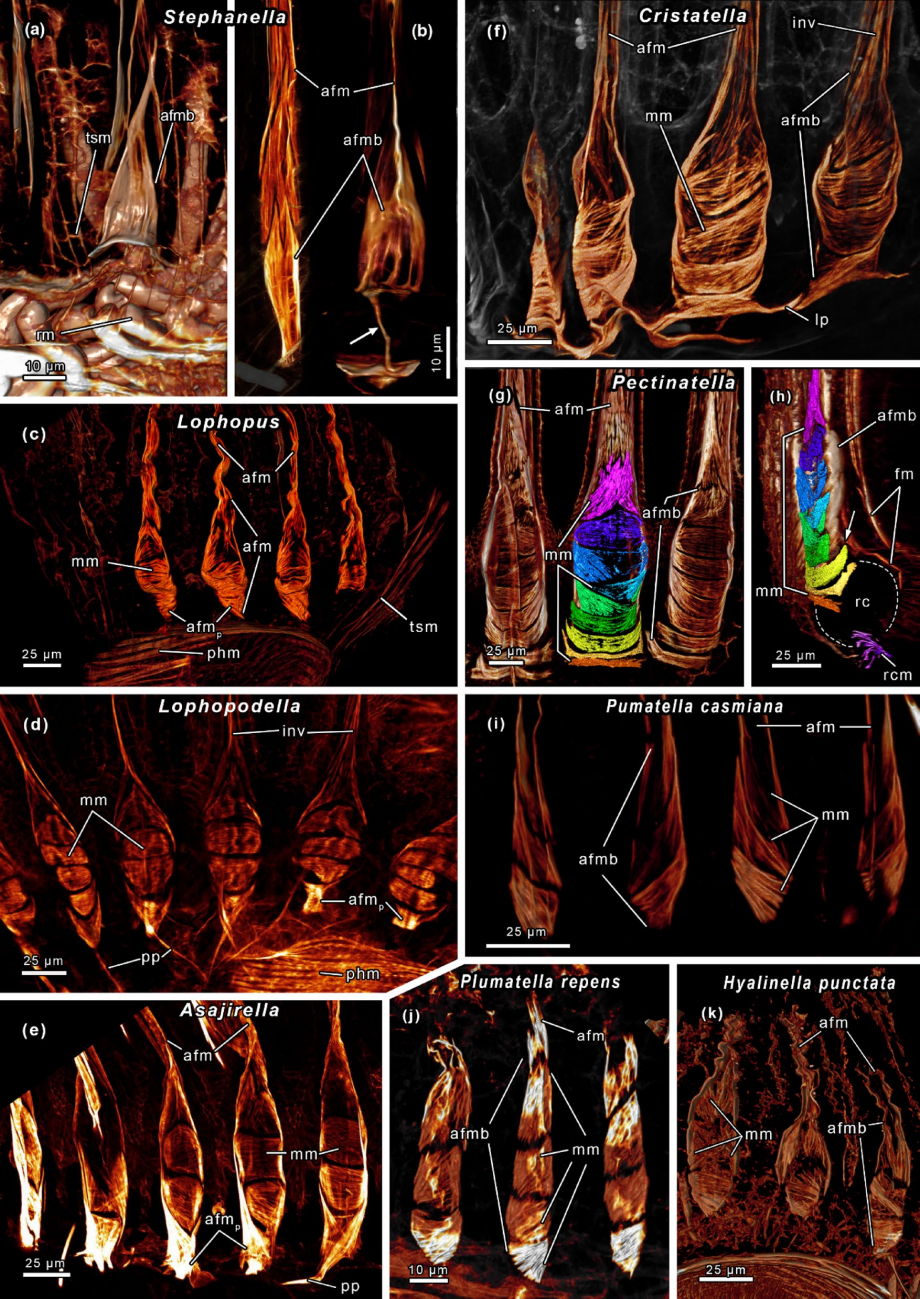
Abfrontal tentacle muscles of several phylactolaemates. **(A-C)** *Stephanella hina*. Abfrontal muscle bases without median muscle bands but longitudinal oriented, stacked muscles. Proximal muscle bases possess a thin process (arrow). **(D)** Oral tentacles of *Lophopous crystallinus*. The abfrontal bases include several median muscle bands. Proximal the abfrontal bases feature crossing muscle strands. **(E)** *Lophopodella carteri*. Abfrontal muscle bases with up to 5 distinct median muscle bands and proximal processes. **(F)** *Asajirella gelatinosa*. Abfrontal bases with at least 3 median bands. Proximal muscle strands cross and ascend laterally of the median muscle bands, distally they result in abfrontal tentacle muscles. Occasionally proximal processes are evident. **(G)** *Cristatella mucedo* with at least 5 median bands tapering distal on the sides. Proximal the bases have lateral projections without connections to adjoining tentacles. **(H-I)** *Pectinatella magnifica* with 9 median bands, similar to *C. mucedo* but without lateral projections. Proximal the median bands extend to the anal side (h, arrow). Ring canal muscles are evident in the proximal border of the ring canal (H). **(J)** *Plumatella casmiana*. Slender abfrontal muscle bases with 3 diagonal orientated muscle bands. **(K)** *Plumatella repens*. Abfrontal bases with 4 distinct packs of diagonal orientated muscle strands. **(L)** *Hyalinella punctata* with broad abfrontal bases, some median muscle bands and longitudinal orientated muscle strands in the distal region. Similar to *Asajirella* the median bands are lined by lateral strands that continue distally as abfrontal tentacle muscles. Abbreviations: **afm** abfrontal tentacle muscle, **afmp** proximal part of the abfrontal tentacle muscle base, **afmb** abfrontal tentacle muscle base, **fm** frontal tentacle muscle, **inv** inverted ‘v’ muscle, **lp** lateral projections, **mm** median muscle bands, **phm** pharynx musculature, **pp** proximal processes, **rc** ring canal, **rcm** ring canal muscle, rm retractor muscle, **tsm** tentacle sheath musculature.

In all phylactolaemates except fredericellids, two lophophoral arms project from the lophophoral base in anal direction and possess lophophoral arm musculature (Figs. 4F and G, 5B and 7). The most prominent lophophoral arm muscles are found in the lophopodid *Asajirella gelatinosa*^43^, whereas the lophopodid *Lophopodella carteri* has much less prominent and *Lophopus crystallinus* only delicate lophophoral arm muscles at its base (Fig. 4F; G). Pectinatellids and cristatellids also have very prominent musculature in the lophophoral arms (Fig. 7C; D^47,69^). On the contrary, hirosellids and the plumatellids *Hyalinella punctata*,* P. emarginata* and *P. repens* have only little lophophoral arm muscles (Fig. 8I^29,47^). Stephanellids and fredericellids lack lophophoral arm muscles. The former have short lophophoral arms, whereas the latter lack them entirely. Consequently, lophophoral arms musculature probably evolved within phylactolaemates and was lost in fredericellids (Figs. 1B; S1^11,15,35,49,74^). The presence and size of lophophoral arm muscles seems to correlate to the size of the lophophoral arms and the lophophore in general.

### Tentacle musculature

Tentacles are provided with two sets of muscles: frontal facing the mouth opening (or feeding tracts on lophophoral arm) and abfrontal on the opposite side (Fig. 5A, B). All tentacle muscles originate from a frontal and abfrontal muscle base, of which the latter is more prominent than the frontal ones (Figs. 5 and 7). Tentacle bases are generally similar in all phylactolaemates but show differences in their finer details.

### Abfrontal muscle bases

Abfrontal tentacle muscles emanate from an abfrontal muscle base, which are more prominent than the frontal ones. They show several features: proximal processes, crossing muscle bundles in the proximal region, transversely oriented median muscle bands, and whether the tentacle muscles ascend continuously or are separated from the base by a gap. These characters vary across families by the number of median muscles and, size of the base and presence/absence of individual features.

**Fig. 9:**
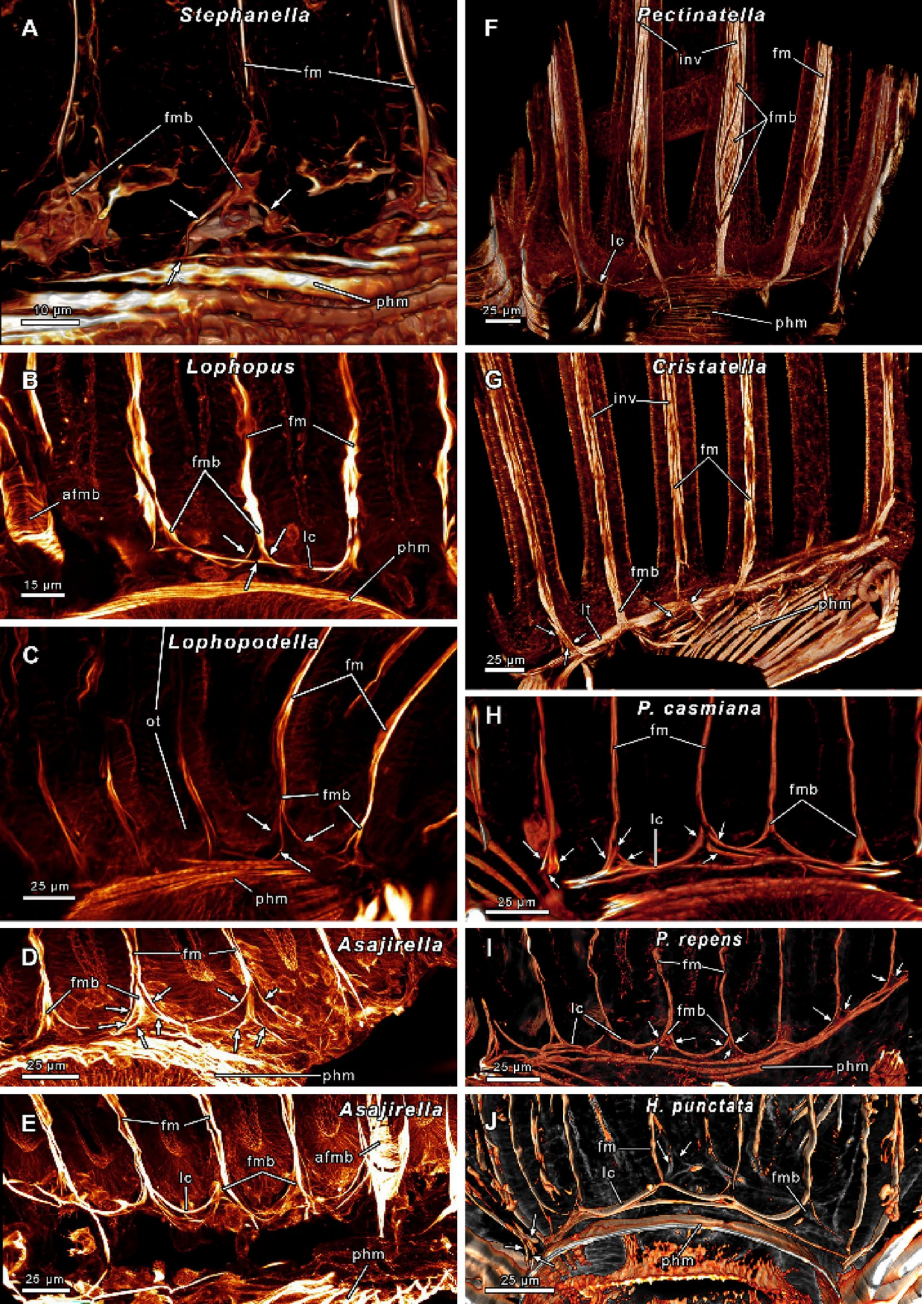
Frontal tentacle muscles of several phylactolaemate families. **(A)** *Stephanella hina*. Slender frontal muscles with at least two rootlets. **(B)** *Lophopus crystallinus*. Frontal muscles with 3 proximal rootlets. Adjoining tentacles are connected via lateral connections. **(C)** *Lophopodella carteri*. Frontal tentacle muscles with three rootlets on the oral side, adjoining tentacles without lateral connections. **(D-E)** *Asajirella gelatinosa*. Frontal muscles with numerous rootlets (arrows). Adjoining tentacles on the oral side feature lateral connections (E). **(F)** *Pectinatella magnifica*. Broad frontal tentacle muscle bases with stacked/diagonal orientated muscle bands. Proximal delicate lateral connections present. **(G)** *Cristatella mucedo*. Pronounced frontal tentacle muscles with inverted ‘v’ structure, 2–3 rootlets and thick lateral connections. (**H)** *Plumatella casmiana* with thin frontal tentacle muscles, 2–3 rootlets (arrows). **(I)** *Plumatella repens*. Like *P. casmiana* with delicate frontal muscles, 2–3 rootlets and lateral connections. **(J)** *Hyalinella punctata*. Similar to *Plumatella* with delicate frontal muscles, mostly 2 rootlets, 3 rootlets in lateral tentacles and connections between adjoining tentacles. Abbreviations: **afmb** abfrontal tentacle muscle base, **fm** frontal tentacle muscle, **fmb** frontal tentacle muscle base, **inv** inverted ‘v’ muscle, **lc** lateral connections, **lt** lateral tentacles, **phm** pharynx musculature, **ot** oral tentacle.

The abfrontal bases of *Stephanella hina* are unique as they lack any median muscle bands but instead consist of several stacked, longitudinal muscles, and include a thread-like proximal process (Figs. 7A; 9 A, B, C^35^). Lophopodids are the only other family with proximal processes, which are least developed in the genus *Lophopus* (Figs. 7B and 9D-F^43^). The large abfrontal bases show a proximal region of crossing muscle fibres in the oral tentacles. After the crossing of these fibres, they extend into the outer, lateral lining of the abfrontal base muscle, especially in *Lophopus* and *Asajirella* (Figs. 7B and 8D and F). Three to five median muscle bands are spanned between this lateral lining in transversal direction (Figs. 7B and 8D and F). Abfrontal tentacle muscles of the tentacles of the lophophoral arms unilaterally connect to the lophophoral arms musculature (Fig. 4F, G^43^).

Cristatellidae and Pectinatellidae have the largest abfrontal bases but lack proximal processes. However, in *Cristatella* the most proximal region of the median muscle band projects laterally to adjacent abfrontal muscle bases, without direct contact (Figs. 7C and 9G^69^). In *Pectinatella*, the proximal median bands are laterally elongated in anal direction (Figs. 7D and 8H and I). With seven (*Cristatella*) and nine (*Pectinatella*) median muscle bands these two genera/families show the highest number observed (Figs. 7C and D and 8G, H and I). Like in lophopodids, the abfrontal bases of the lophophoral arm tentacles are connected to the lophophoral arms musculature (Fig. 7C, D^43,47^).

Hirosellidae and Fredericellidae have the smallest abfrontal bases, composed of obliquely orientated muscle bundles (Fig. 7E, F). Both have a small gap between the base and the tentacle giving them a similar appearance. *Fredericella* lacks lophophoral arm musculature and its abfrontal muscle bases are hence not connected to other muscles. Despite the presence of lophophoral arm muscles in *Hirosella*, they are not connected to the abfrontal tentacle muscles (Fig. 7F^29,49^). In plumatellids, the abfrontal muscle bases comprise three (e.g. *P. casmiana*) to four (e.g. *P. repens*) median muscle bands (Figs. 7G and 8J-K). The genus *Hyalinella* differs by having crossing median muscle bands spanned between a lateral lining of longitudinal muscles, similar to lophopodids (Figs. 7G and 8L).

Overall, the abfrontal muscle bases appear similarly structured among the clades with the exception of median muscle bands, that evolved within phylactolaemates (Figs. 1B; S1). Nevertheless, there is notably variation in finer anatomical details e.g. the gap between the base and the tentacle muscle and the presences of proximal processes. Both features are recognised as ancestral according to ancestral state analysis (Fig. S1). Considerable differences even with between closely related clades, such as, *Hirosella*/*Pectinatella*/*Cristatella*, or within plumatellids complicate evolutionary interpretations and thus suggest limited phylogenetic relevance.

### Frontal tentacle muscles

The frontal tentacle muscles of phylactolaemates show less structural variation when compared to the abfrontal ones and consist mostly of a thin muscle ascending within each tentacle. When present, the bases comprise a variable number of rootlets that can be connected to either neighbouring tentacles, the lophophoral arms muscles or to a distinct circum-oral muscle ring, close to the pharynx musculature (Figs. 5D, E, F, G and H and 9).

*Stephanella hina* has barely any muscular base and the frontal tentacle muscle originate more distal than the abfrontal muscles, and are inconspicuous (Fig. 5D A^35^). In lophopodids, the genera *Lophopus* and *Asajirella* feature three to five rootlets that connect to adjacent frontal muscles, thus forming a continuous connection between the oral tentacles (Figs. 5A, G, H; 8 B, D, E^43^). The muscular bases are prominent in *Asajirella*, but more delicate in the genus *Lophopus*. The genus *Lophopodella* has up to three rootlets that are not connected to other frontal muscles (Figs. 5E and 9C).

Pectinatellidae and Cristatellidae possess the most prominent frontal tentacle muscle bases (Fig. 5F and G F; G). *Pectinatella* features the only bases with obliquely orientated muscle bands, while the lateral connections of the bases are very delicate (Fig. 5F F^47^). The frontal muscles in *Cristatella* are thick muscle bands that proximally connect to a prominent circum-oral muscle ring (in oral/lateral tentacles) with two to three rootlets, or to the lophophoral arms muscles in the corresponding tentacles (Figs. 5G and 8G^47,69^). In plumatellids, hirosellids and fredericellids the frontal muscles are inconspicuous and possess two to three rootlets that connect to adjoining frontal muscles and thereby form a circum-oral muscle ring (Figs. 5H; 8I, J^49^). The frontal muscles of the tentacles of the lophophoral arms root in the muscles of the latter via a single rootlet, whereas in fredericellids they form a continuous muscular connection between adjacent tentacles (see citation above).

Frontal tentacle muscles are connected to lophophoral arm muscles. Frontal muscles of the lateral tentacles mostly have three rootlets, whereas tentacles located within the lophophoral concavity possess only one to two rootlets. Frontal muscles of the tentacles located along the anal side of the lophophoral arms eventually connect with only one rootlet to the lophophoral arms musculature (Fig. 4G A, B, H^43,49^) Hardly any conclusion regarding the ancestral state can be drawn with current data. However, a maximum of two rootlets in oral frontal tentacle muscles is the state found in most families (Fig. S1). Further, different rooting of the tentacle muscles may have functional implications in accordance with different feeding behaviour, as *Fredericella* for example utilises its entire lophophore as a snap-like mechanism to prevent the prey escaping the water current established via cilia; and *Cristatella* e.g. bends its lophophoral arms backwards to selectively reject unsuitable particles.

### Ring Canal musculature

The ring canal is a coelomic channel on the oral side of the lophophoral base. On its lateral sides, it is confluent with the remaining coelomic cavity and supplies the oral tentacles of the lophophore (Fig. 5A). Short muscle fibres radiate along the proximal lining of the ring canal in the phylactolaemates Cristatellidae, Pectinatellidae and several Plumatellidae (Fig. 5A A, B, E, F; 7 A-C; 9I^28,47,69^). Stephanellidae, Fredericellidae and Hirosellidae lack these muscles. They also seem to be absent in lophopodids, but some histological data indicates their presence in *Lophopus* and *Lophophodella* (see Figs. 17 A; 20B, C; 21 A in Bibermair et al.^43^). It was previously suggested that ring canal muscles have been overlooked in plumatellids^28^as supported on the current observations on *Plumatella repens*. Suffice to say, these muscles could still remain undetected, but from current data evolved at least twice within Phylactolaemata (Fig. S1). The function of these muscles is unknown.

### Intertentacular membrane

**Fig. 10:**
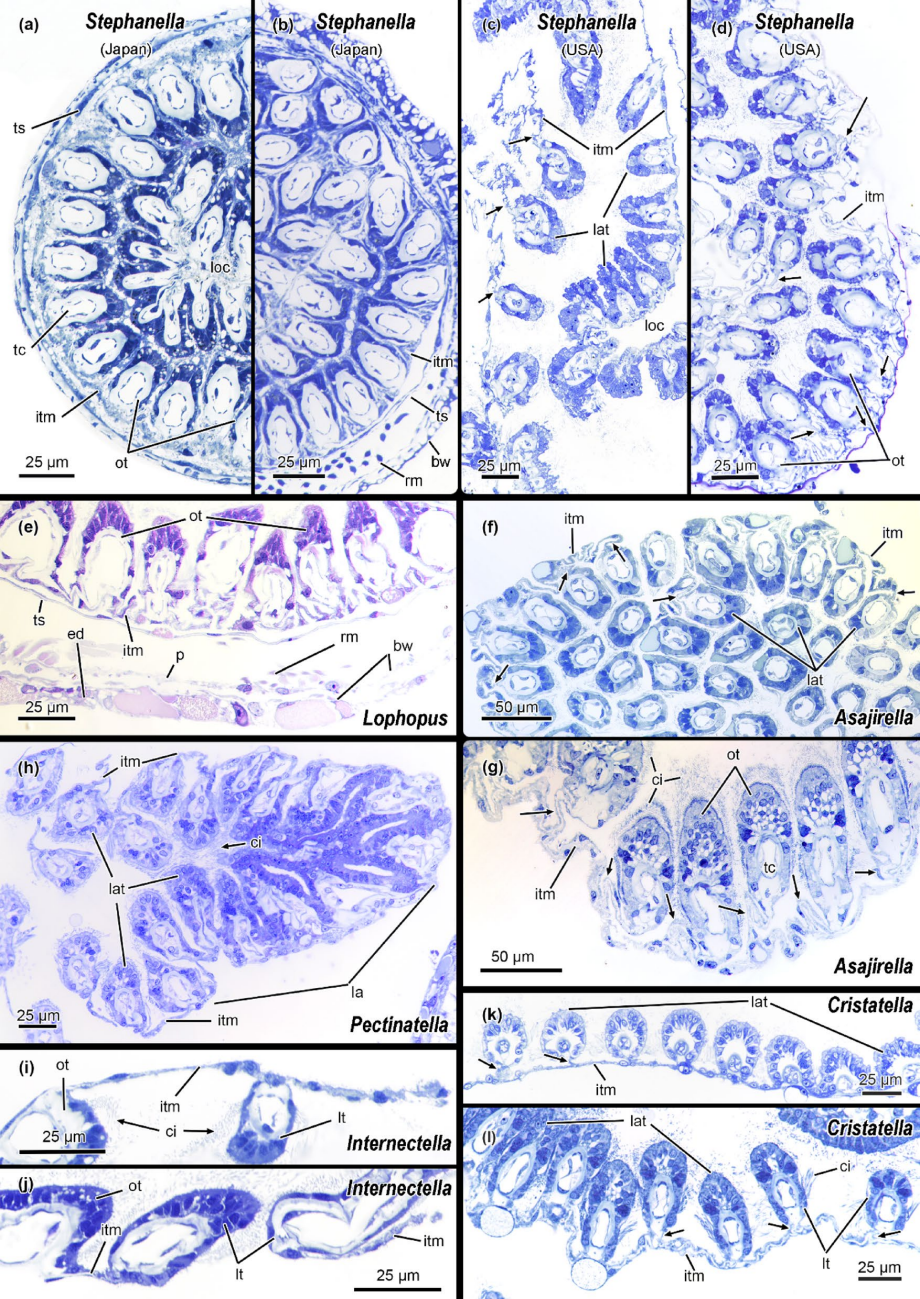
Histological details of the lophophore and intertentacular membrane of several phylactolaemate representatives. **(A-B)** *Stephanella hina* from Japan. The intertentacular membrane without gap in the oral tentacles and integrated into the abfrontal side of the oral (A) and lophophoral arm (B) tentacles. **(C-D)** *S. hina* from U.S.A. The intertentacular membrane is attached to the tentacles of the lophophoral arms (C) and the oral tentacles (D) via lamellae. **(E)** Intertentacular membrane between oral tentacles of *Lophopus crystallinus* without a gap. The membrane is fully integrated into the abfrontal epithelium of all tentacles. **(F-G)** Tentacles of the lophophoral arms (F) and oral tentacles (G) of *Asajirella gelatinosa*. The membrane is attached to the tentacles via lamellae (arrows) in all tentacles. The membrane between the oral tentacles has no gap. **(H)** Tentacles of the lophophoral arm of *Pectinatella magnifica*. The intertentacular membrane is fully integrated into the epithelium of the tentacle. **(I-J)** the intertentacular membrane of the fredericellid *Internectella bulgarica* without any lamellae. **(K-L)** *Cristatella mucedo*. The intertentacular membrane is attached to tentacles via lamellae. Abbreviations: **bw** body wall, **ci** cilia, **ed** epidermis, **itm** intertentacular membrane, **la(t)** lophophoral arm (tentacles), **loc** lophophoral concavity, **lt** lateral tentacles, **ot** oral tentacles, **p** peritoneum, **rm** retractor muscle, **tc** tentacle coelom, **ts** tentacle sheath.

The intertentacular membrane is a thin epidermal duplicature between the proximal area of adjacent tentacles, and apomorphic for phylactolaemates (Fig. 10^15,17,18,53,73^). Differences among phylactolaemate clades are found in the attachment of the intertentacular membrane to the tentacles. The membrane is either fully integrated in the abfrontal side of tentacles, or it attaches to the tentacles via a thin lamella. The former is found in plumatellids, fredericellids^49^, pectinatellids and most lophopodids. Cristatellids, hirosellids and stephanellids possess the second, lamellate form (Fig. 10C, D, K, L). Also, oral and lateral tentacles of the lophopodid *Asajirella gelatinosa* show lamellae (Fig. 10F, G). Moreover, cristatellids and stephanellids can have both conditions (Fig. 10A, B, C, D, K, L). *Stephanella hina* also shows high variability, without lamella (Fig. 10A, B) or with lamella (Fig. 9C, D), which might cooccur^35^and indicate a high variability in the presence or absence of lamellae.

Another character of the intertentacular membrane is the presence of a gap in the membrane between the median oral tentacles. This gap can be found in several plumatellids, fredericellids and hirosellids whereas stephanellids, lophopodids, pectinatellids and cristatellids possess a continuous membrane between all tentacles^15,49,75^. Functionally, this empty space remains cryptic, but has been suggested to be involved as possible outflow tracts for feeding currents. Its presence in the more tubular, elongated, but also erect taxa indicates that it might be more of a functional character rather than phylogenetically relevant. Ultimately, the absence of a gap between oral tentacles and the presence of a lamella are indicated as the ancestral condition (Figs. 1A; S1).

### Development

Sexual reproduction of phylactolaemate bryozoans has recently been summarized in Bibermair et al.^56^. All investigated phylactolaemates brood embryos in embryo sacs^16,63,76–78^. A ciliated mantle larva develops within the embryo sac^3,16,51,56,63,79^. Embryos are nourished by a placental analogue in all studied phylactolaemates via a placental ring, which is considered as location of matrotrophic nutrient exchange but also anchors the larva to the maternal zooid^3,76^. The placental ring has been reported in plumatellids (^16,51^, see also Ostrovsky et al.^80^), whereas the placental contact is spot-like in fredericellids^63^, cristatellids^81,82^and pectinatellids^63^. Data for stephanellids, lophopodids and hirosellids is currently missing. Mantle larvae are characterized by a ciliated hull, or mantle, which serves for propulsion and houses one to four polypides within the mantle^15,16,39,51,63,79,83,84^. Two polypides are present in larvae of Plumatellidae^16,84^and Lophopodidae^83,85,86^. Fredericellidae and Hirosellidae have a single polypide^14,63^, whereas Cristatellidae and Pectinatellidae carry four polypides^87,88^. Sexual reproduction and larvae of stephanellids, the earliest branch of phylactolaemates, is unknown, which renders ancestral state reconstruction difficult. However, mantle larvae probably are apomorphic for phylactolaemates or at least evolved after the Stephanellidae split. Two polypides seem to be the ancestral state for most phylactolaemates whereas four is a shared character of Cristatellidae and Pectinatellidae. A single polypide in the larva seems to be a uniting character of Fredericellidae and Hirosellidae. Also, available data indicate, that a spot-like placenta is probably ancestral for the clade comprising Fredericellidae, Hirosellidae, Cristatellidae and Pectinatellidae, whereas due to the lack of data, the ring-like placenta is so far apomorphic for Plumatellidae (Fig. S1; Table 2).

### Statoblasts

Statoblasts are chitinized capsules that enclose germinative tissue and yolk cells^89^. Statoblasts have various morphologies but usually have an outer periblast and a central capsule that contains the germinative and yolk cells^11,51,89,90^. The most common type of statoblasts are floatoblasts, which have a gas-filled periblast, the annulus^11,12^, and are present in all families except the genus *Fredericella*. They can carry spines and are then referred to as spinoblasts, typical for Pectinatellidae, Cristatellidae and some Lophopodidae^12,22,45,51^. Spinoblasts were traditionally considered as derived^17,53^, whereas more recent phylogenies place lophopodids as earlier branching. Modifications of the typical floatoblast are found in sessoblasts, which are attached to the substrate and have a reduced or vestigial annulus. They are present in the early branching stephanellids, the late-branching plumatellids and hirosellids^11,37,45,89^. Developmental data on sessoblast formation supports an independent evolution of these forms in stephanellids and the plumatellid-fredericellid-hirosellid branch^91^. Further simplified statoblasts are piptoblasts of fredericellids, which completely lack or have an even higher reduced periblast, but sometimes feature a cementing apparatus similar to sessoblasts. The genus *Internectella* is the only fredericellid with a floatoblast that has an annulus on only one half^11,45,92^. Statoblasts in form of floatoblasts are ancestral for all Phylactolaemata (Fig. S1). Sessoblasts as binary character are indicated as ancestral condition in the ancestral state reconstruction. However, they evolved twice independently, once in Stephanellidae and in the clade comprising all remaining families excluding Lophopodidae, when their cystigenic/deuteroplasmatic origin is taken into account (Fig. S1). In piptoblasts, the vestigial attachment apparatus (see Wood & Okamura^34^is indicative of their sessoblast origin, whereas Cristatellidae and Pectinatellidae are considered to have lost sessoblasts, possibly related to their colonial locomotion similar to Lophopodidae, which also lack sessoblasts.

### Neuroanatomy

Recent studies investigated the neuroanatomy of phylactolaemates by ultrastructural, immunological and 3D - reconstructing approaches (e.g^25,29,35,43,48,55,72,74,93–95^).

The central nervous system of phylactolaemate bryozoans consists of a cerebral ganglion, located at the base of the lophophore between the pharynx and intestine. The ganglion is typically a fluid-filled vesicle in all phylactolaemates, which contrasts the more compact and ovoid-shaped ganglion of gymnolaemates^3,25,74^. Its size and morphology can vary between families as seen in lophopodids, where it is notably larger and contains a pronounced lumen, flanked by two prominent epistomial horns—pyramidal structures with large lumina that extend into the epistome. The latter are unique for lophopodids whereas thin epistomial neurite bundles exist in probably all phylactolaemates^43^.

From the cerebral ganglion, a circum-pharyngeal nerve ring projects around the pharynx and innervates the oral/lateral tentacles. On the anal side, two ganglionic horns project into the lophophoral arms. They are usually well-developed and are only reduced in fredericellids, which correlates with the lack of distinct lophophoral arms^74^. In the early branching stephanellids, the ganglionic horns are unique by bending medially towards the mouth opening, which gives it a crescent shape instead of projecting into the short lophophoral arms^35^.

The innervation of the tentacles in phylactolaemates generally follows a similar organization (for detailed description see e.g^.43,55,75^). Some minor differences are reported from the number of lateral nerves connecting neighbouring frontal tentacle nerves^55^. Otherwise, each tentacle has six tentacle neurite bundles, three frontal and three abfrontal. Peripheral nerve plexuses project via several neurite bundles from the cerebral ganglion and the circum-oral nerve ring. It is most prominent at the pharynx, least dense in the intestine and appears consistent among families. Another nervous plexus is documented in the body wall and the tentacle sheath. It also originates from the cerebral ganglion/circum-oral nerve ring and is similar in all investigated phylactolaemates. Minor differences exist, as most phylactolaemates have crosslinked longitudinal neurites, while in some genera of different families, as the plumatellid *Hyalinella* and the lophopodid *Asajirella* lack ‘circular’ neurites entirely^43,48,55,72^. Despite these minor differences, the available data on the neuroanatomy of phylactolaemates indicate a rather uniform architecture. Studies on neurotransmitters (serotonin-like immunoreactivity, FMRF-like immunoreactivity, catecholamine-like immunoreactivity) are few, but are generally only found in the cerebral ganglion of most bryozoans^29,95^. Hence, it draws only a small picture of the nervous system with little variation^95–97^.

## Conclusion

The present study provides the first comprehensive analysis across all recently recognised phylactolaemate families (except Tapajosselidae, of which no living specimen has been observed so far). It combines historical, recent and novel data, that mainly focused on individual families, into a comparative analysis. A broad character matrix was compiled and blotted onto a recent phylogeny. The resulting ancestral state analysis suggests that colonies with serially arranged zooids with large interzooidal distances and non-encrusting ectocysts are ancestral. The myoanatomy of the body wall, apertural region and digestive tract is largely conserved across families, although occasional deviations exist, e.g. a third layer of body wall muscles.

Detailed comparison of the lophophoral base revealed lineage specific variations: despite the lack of some muscle sets in certain families, the presence of epithelial and transversal muscles in the epistome is ancestral; lophophoral arms and tentacle muscles are similarly structured but vary in subtle and complex ways so that they are challenging to classify. There are recurring muscle systems as the cricumpharyngeal muscle ring, proximal epistome muscles and ring canal muscles, that are widespread but often poorly documented. The nervous system is notably conserved with only minimal variation.

Further ancestral conditions are found in the intertentacular membrane with lamellae that serve as contact structures to the tentacles, while the presence of a gap in the intertentacular membrane is novel character within phylactolaemates. Also, floatoblasts are recognised as ancestral, while hooks/spines or the formation of piptoblasts are derived.

Character evolution in phylactolaemates is impaired by numerous small-scale adaptations and also limited fossil evidence. Combined with the monotypic nature of most extant families, the reconstruction of a last common ancestor of phylactolaemates is difficult. Many morphological traits likely reflect adaptations to specific microhabitats. While all phylactolaemates are sessile suspension feeders, differences in substrate preference, feeding dynamics and flow dynamics are less explored. Eventually, future research should integrate ecological context and function to morphological data in order to fully understand the evolutionary adaptations of this group.

## Supplementary Information

Below is the link to the electronic supplementary material.

Supplementary Material 1

Supplementary Material 2

## Data Availability

The datasets produced and/or analysed during the current study are available from the corresponding author on reasonable request. All data needed are included in the paper or available as supplemental file from the publisher.

## Declarations

Specimens were collected in accordance to local guidelines.
