# Supplementary material for: Morphological character evolution and ancestral state reconstruction in phylactolaemate bryozoans
Source: Sci Rep. 2026 Mar 23;16:15106. doi: 10.1038/s41598-026-40223-0 (PMC13171971; doi:10.1038/s41598-026-40223-0)

Character 1: Character 1
Marginal prob. recon. with model Mk1 (est.) [rate 0.22630648 [est.]] -log L.:6.10761715 (Opt.: width 0.0) Reporting likelihoods as Proportional Likelihoods; Threshold when decisions made: 2.0 Calc. by Maximum likelihood reconstruct (Generic categorical) (id# 1227)

0
1

Character:
colony morphology: wide spaced / serial arranged zooids
0= character absent
1= character present

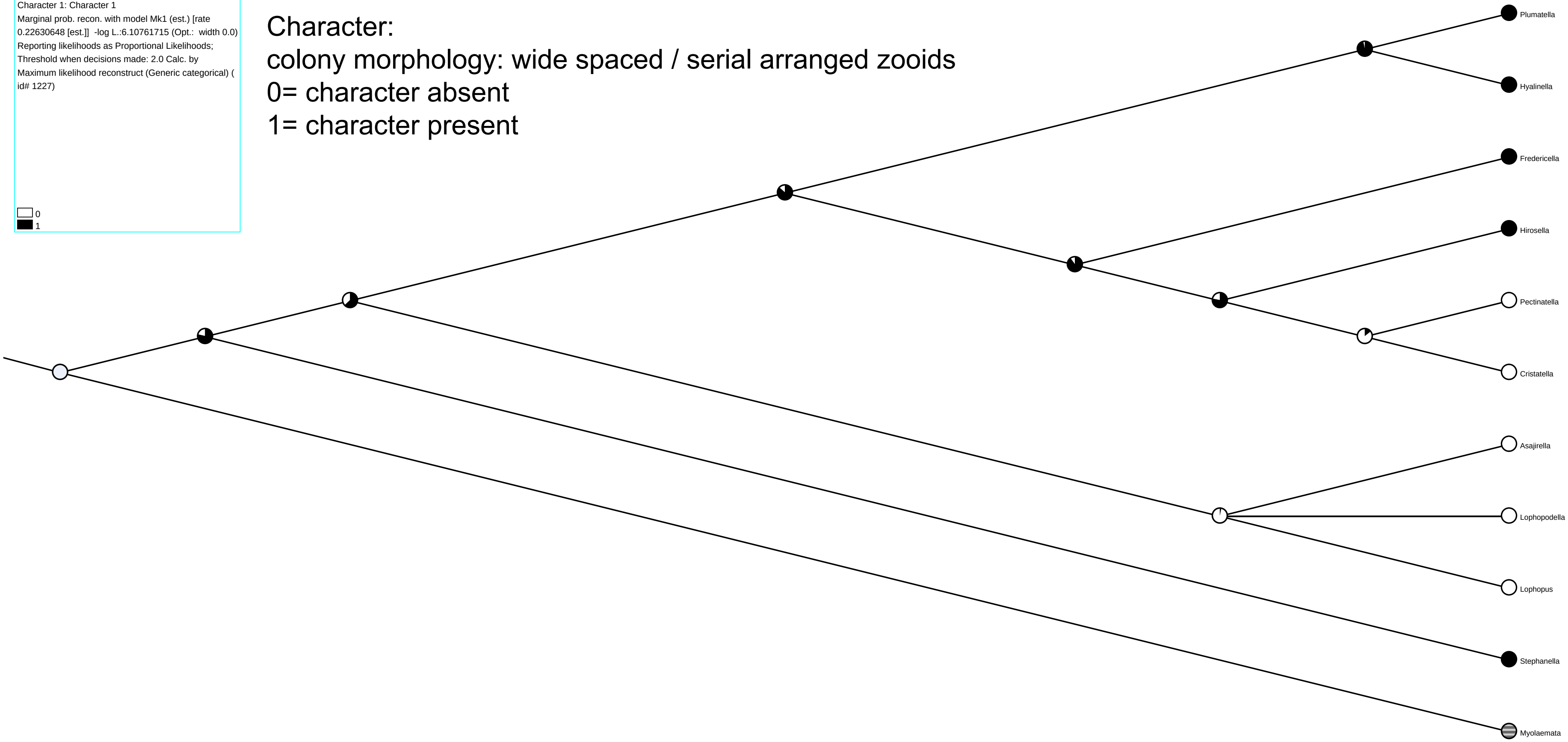

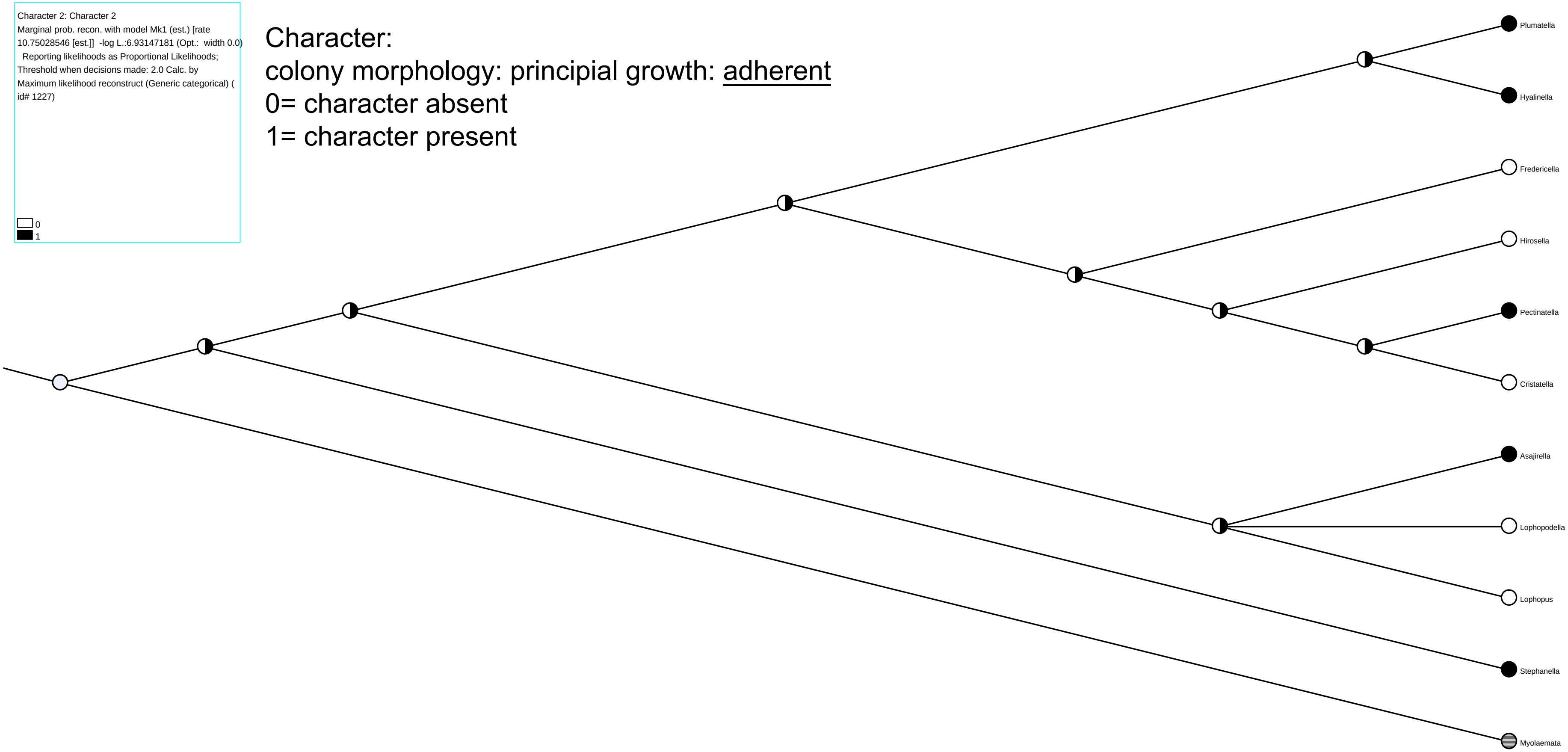
Character 2: Character 2
Marginal prob. recon. with model Mk1 (est.) [rate 10.75028546 [est.]] -log L.:6.93147181 (Opt.: width 0.0)
Reporting likelihoods as Proportional Likelihoods;
Threshold when decisions made: 2.0 Calc. by
Maximum likelihood reconstruct (Generic categorical) (id# 1227)
0
1
Character:
colony morphology: principial growth: adherent
0= character absent
1= character present
Plumatella
Hyalinella
Fredericella
Hirosella
Pectinatella
Cristatella
Asajirella
Lophopodella
Lophopus
Stephanella
Myolaemata

Character 3: Character 3
Marginal prob. recon. with model Mk1 (est.) [rate 0.17691727 [est.]] -log L.:5.79478713 (Opt.: width 0.0) Reporting likelihoods as Proportional Likelihoods; Threshold when decisions made: 2.0 Calc. by Maximum likelihood reconstruct (Generic categorical) (id# 1227)

0
1

Character:
colony morphology: principial growth: errect
0= character absent
1= character present

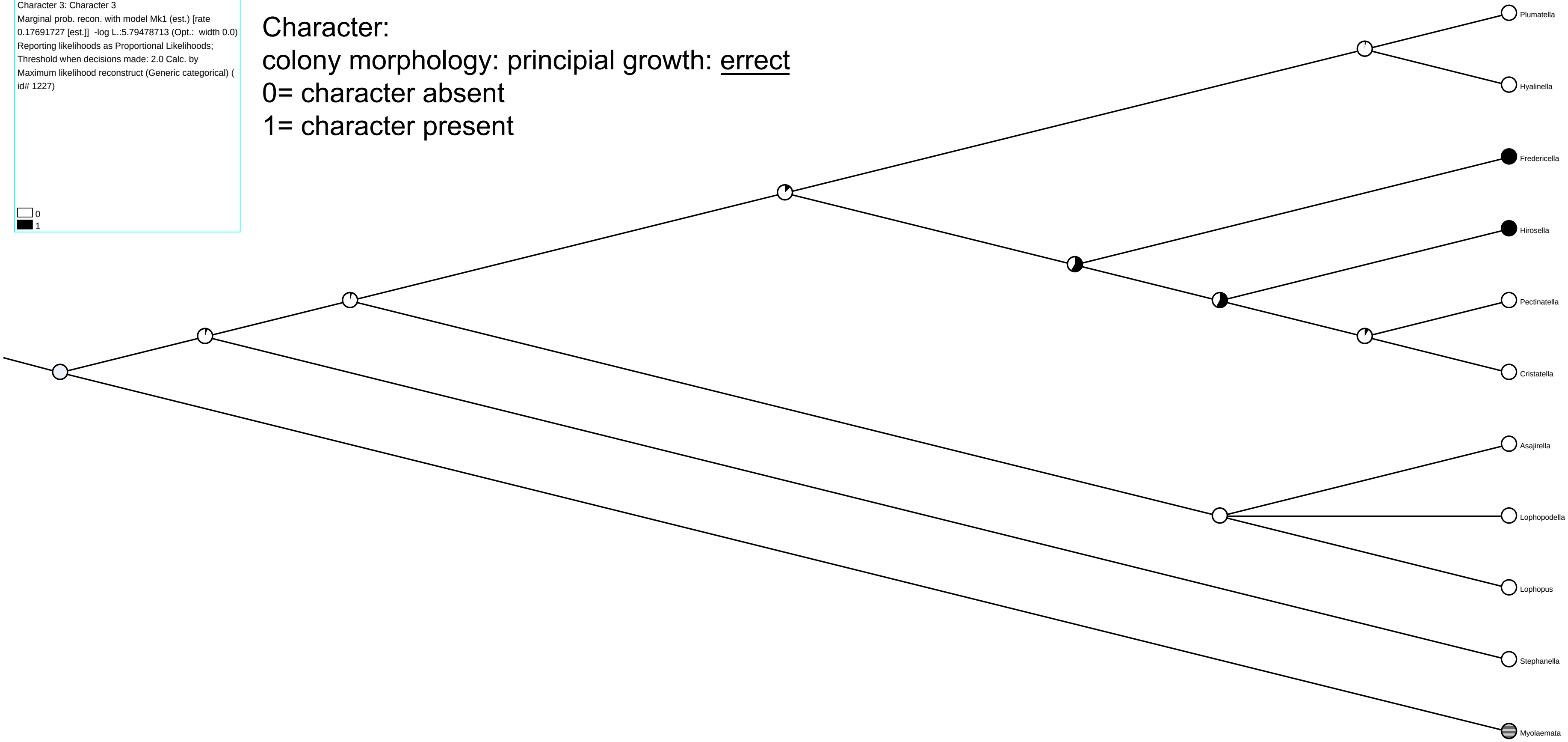

Character 4: Character 4
Marginal prob. recon. with model Mk1 (est.) [rate 0.51349549 [est.]] -log L.:6.87861493 (Opt.: width 0.0) Reporting likelihoods as Proportional Likelihoods; Threshold when decisions made: 2.0 Calc. by Maximum likelihood reconstruct (Generic categorical) (id# 1227)

0
1

Character:
colony morphology: principial growth: <u>creeping</u>
0= character absent
1= character present

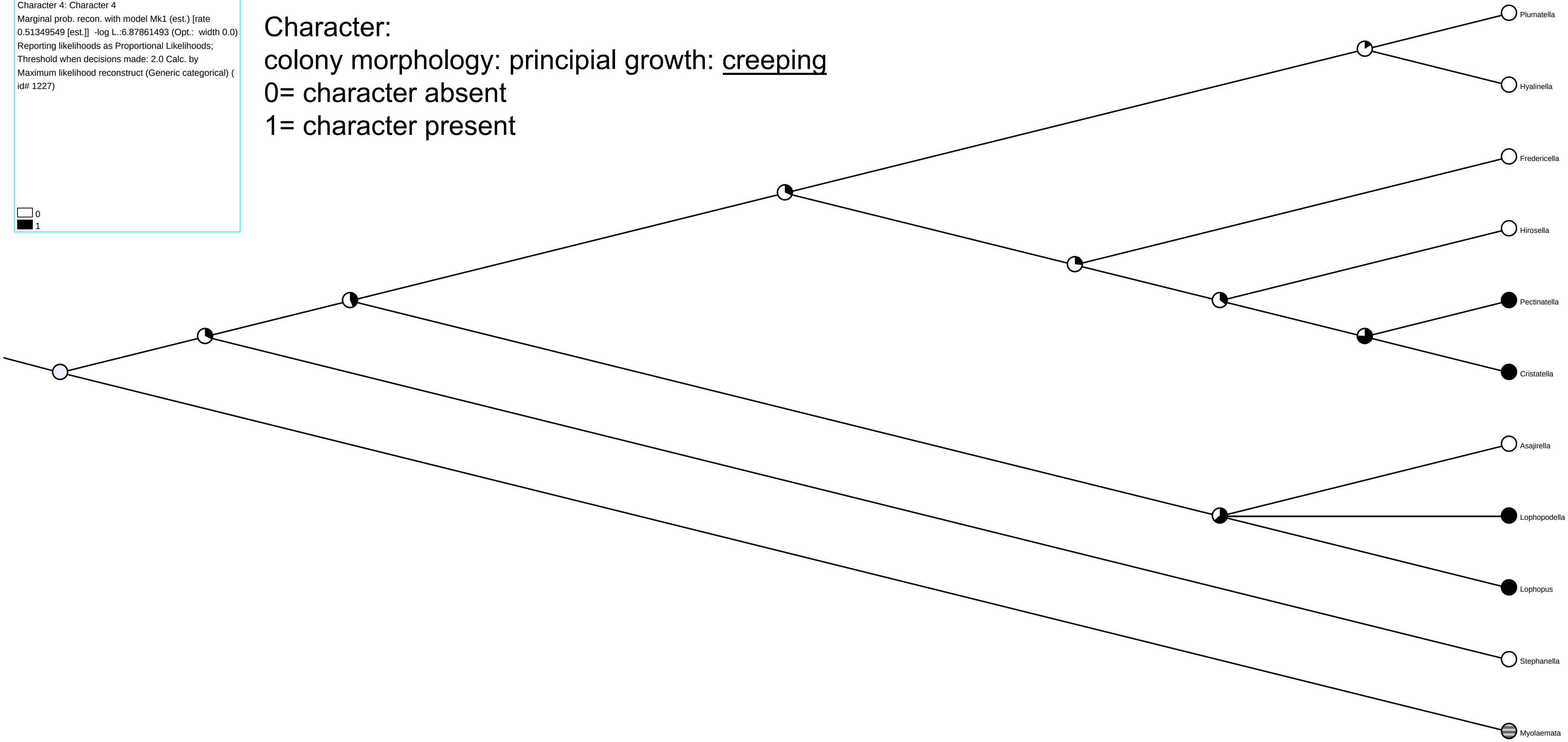

Character 5: Character 5
Marginal prob. recon. with model Mk1 (est.) [rate 0.21882639 [est.]] -log L.:5.9757088 (Opt.: width 0.0) Reporting likelihoods as Proportional Likelihoods; Threshold when decisions made: 2.0 Calc. by Maximum likelihood reconstruct (Generic categorical) (id# 1227)

Character:
colony morphology: ectocyst: encrusting
0= character absent
1= character present

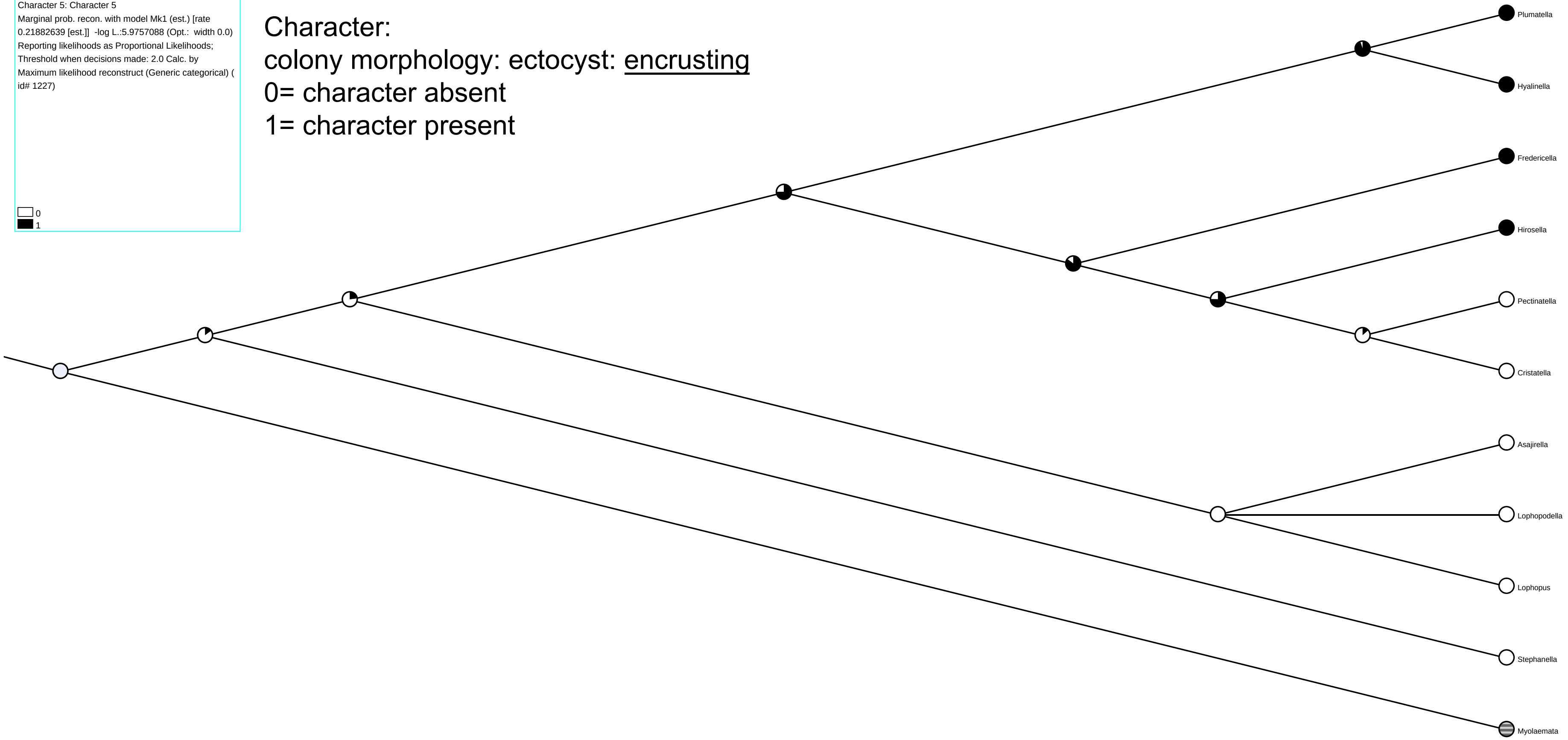

Character 6: Character 6
Marginal prob. recon. with model Mk1 (est.) [rate 9.47586718 [est.]] -log L.:6.93147181 (Opt.: width 0.0) Reporting likelihoods as Proportional Likelihoods; Threshold when decisions made: 2.0 Calc. by Maximum likelihood reconstruct (Generic categorical) (id# 1227)

Character:
colony morphology: ectocyst: gelatinous
0= character absent
1= character present

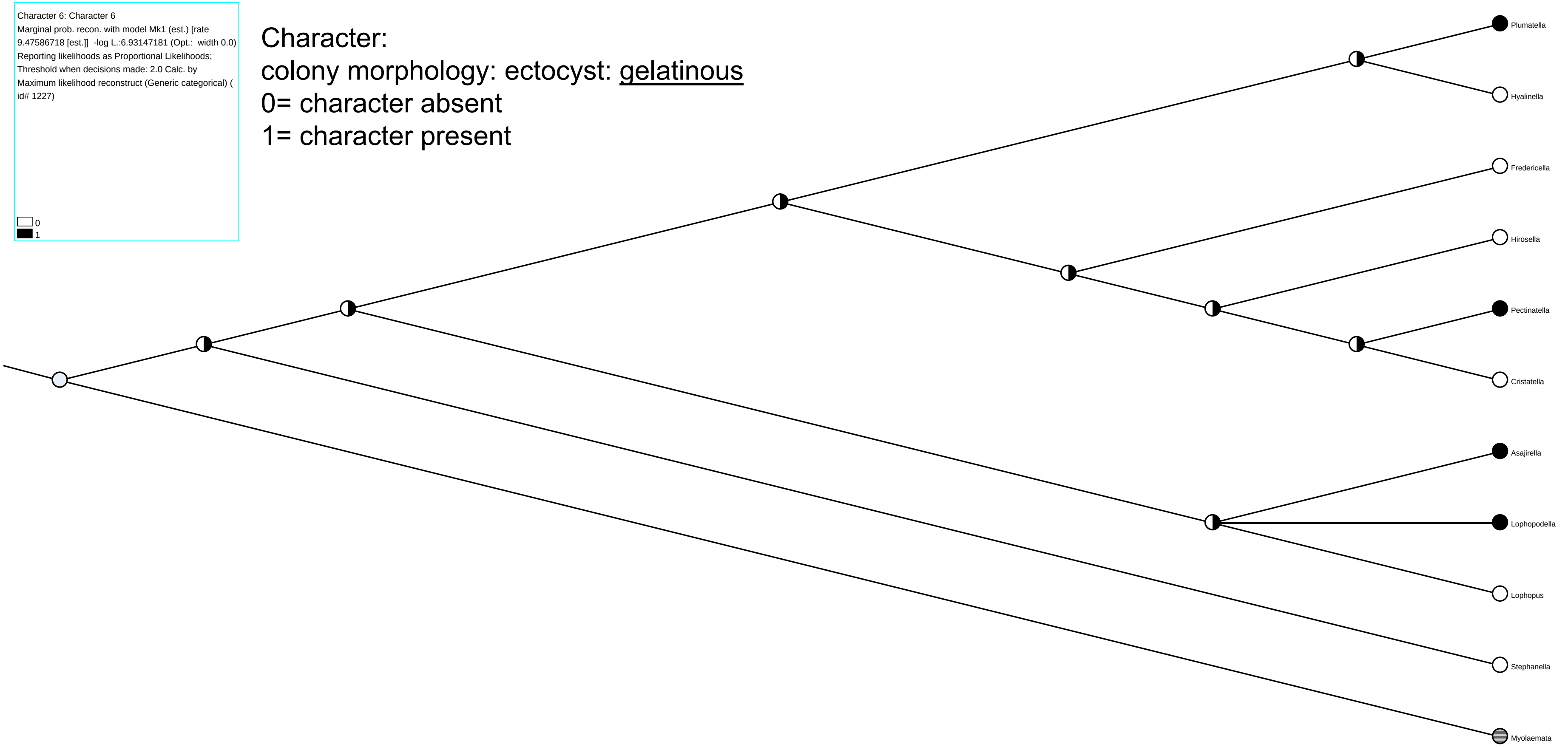

Character 7: Character 7
Marginal prob. recon. with model Mk1 (est.) [rate 10.73917347 [est.]] -log L.:6.93147181 (Opt.: width 0.0)
Reporting likelihoods as Proportional Likelihoods; Threshold when decisions made: 2.0 Calc. by Maximum likelihood reconstruct (Generic categorical) (id# 1227)

0
1

Character:
colony morphology: ectocyst: membranous
0= character absent
1= character present

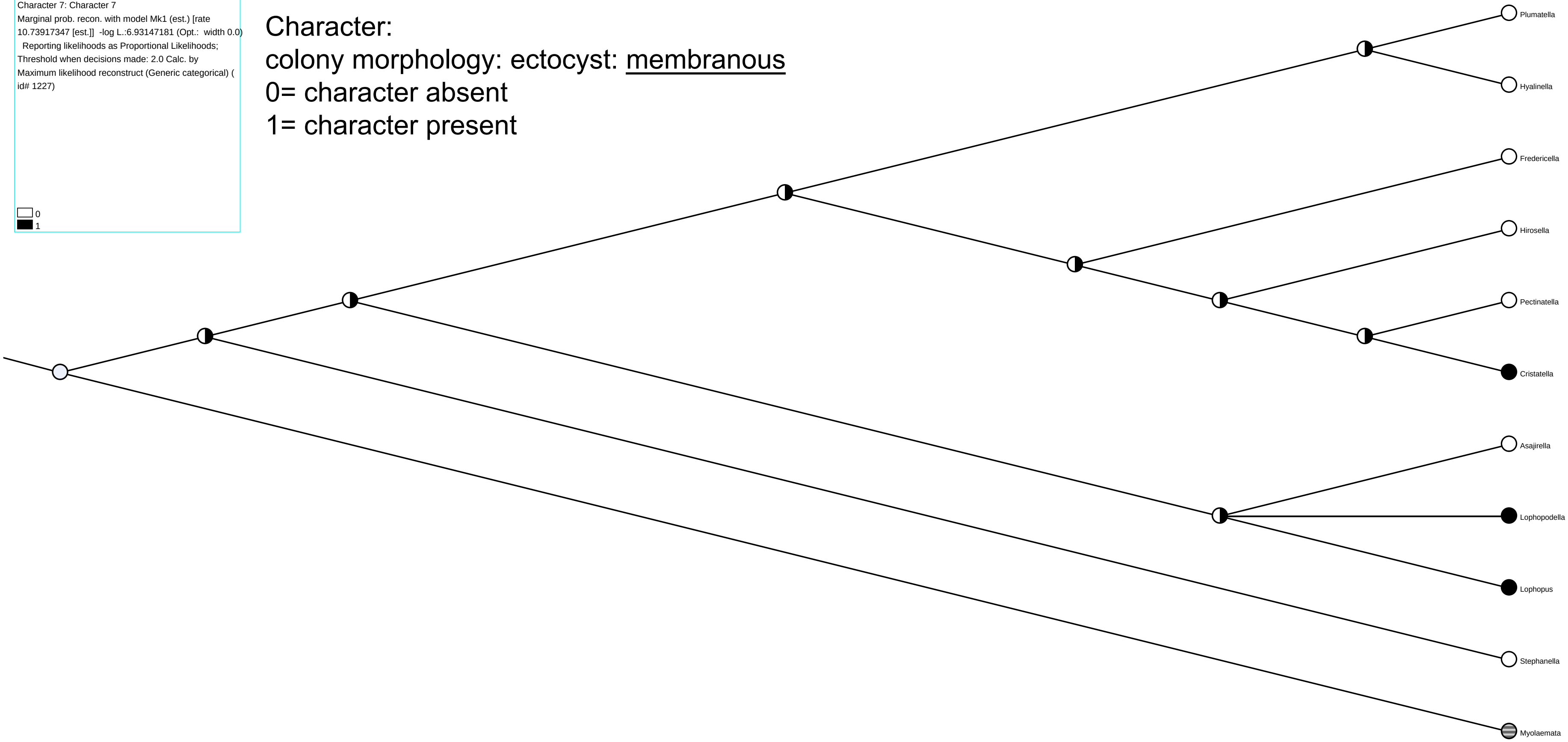

Character 8: Character 8
Marginal prob. recon. with model Mk1 (est.) [rate 0.06538409 [est.]] -log L.:3.76788124 (Opt.: width 0.0) Reporting likelihoods as Proportional Likelihoods; Threshold when decisions made: 2.0 Calc. by Maximum likelihood reconstruct (Generic categorical) (id# 1227)

0
1

Character:
colony morphology: ectocyst: tubular
0= character absent
1= character present

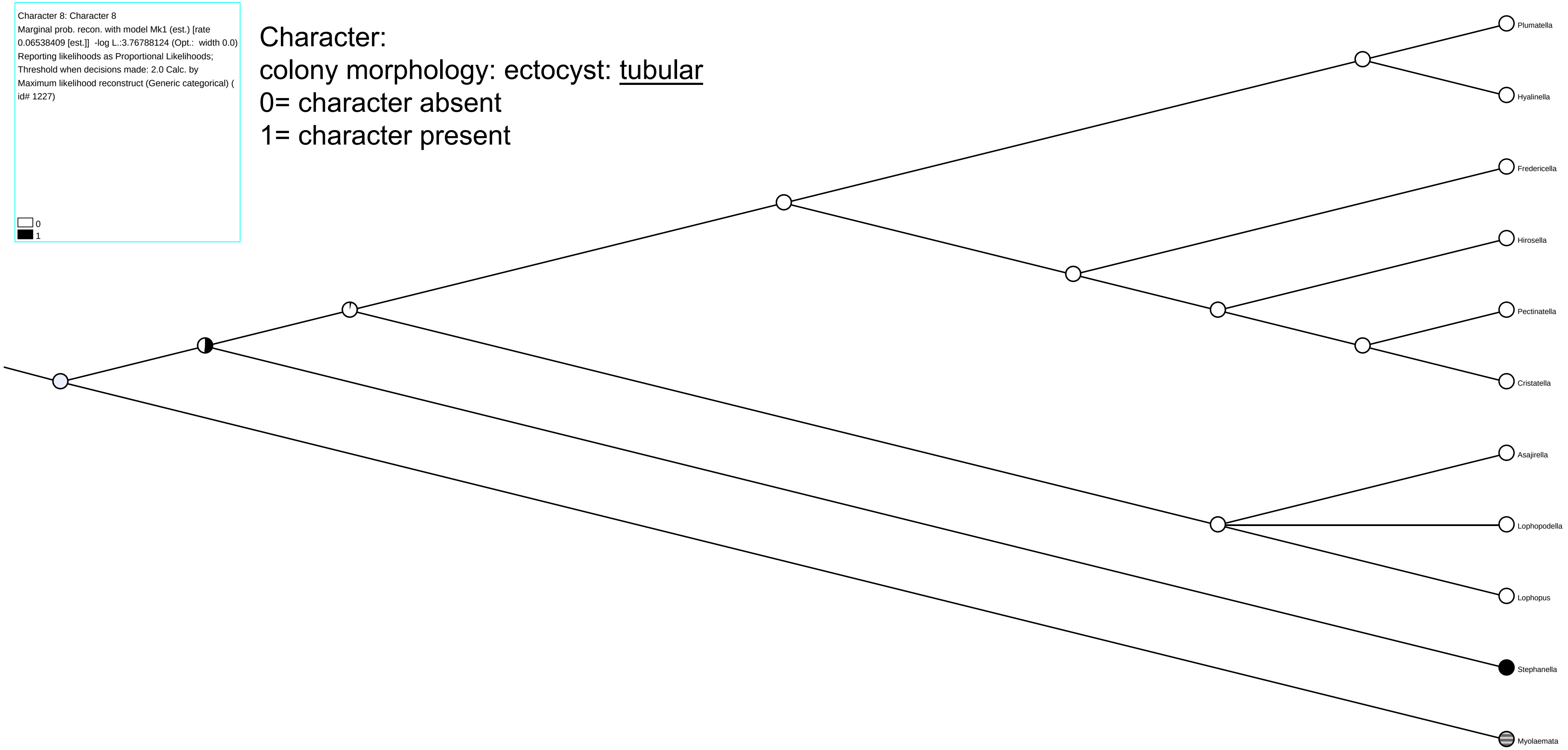

Character 9: Character 9
Marginal prob. recon. with model Mk1 (est.) [rate 0.67236206 [est.]] -log L.:4.84691472 (Opt.: width 0.0) Reporting likelihoods as Proportional Likelihoods; Threshold when decisions made: 2.0 Calc. by Maximum likelihood reconstruct (Generic categorical) (id# 1227)

0
1

Character:
colony morphology: ectocyst: loose
0= character absent
1= character present

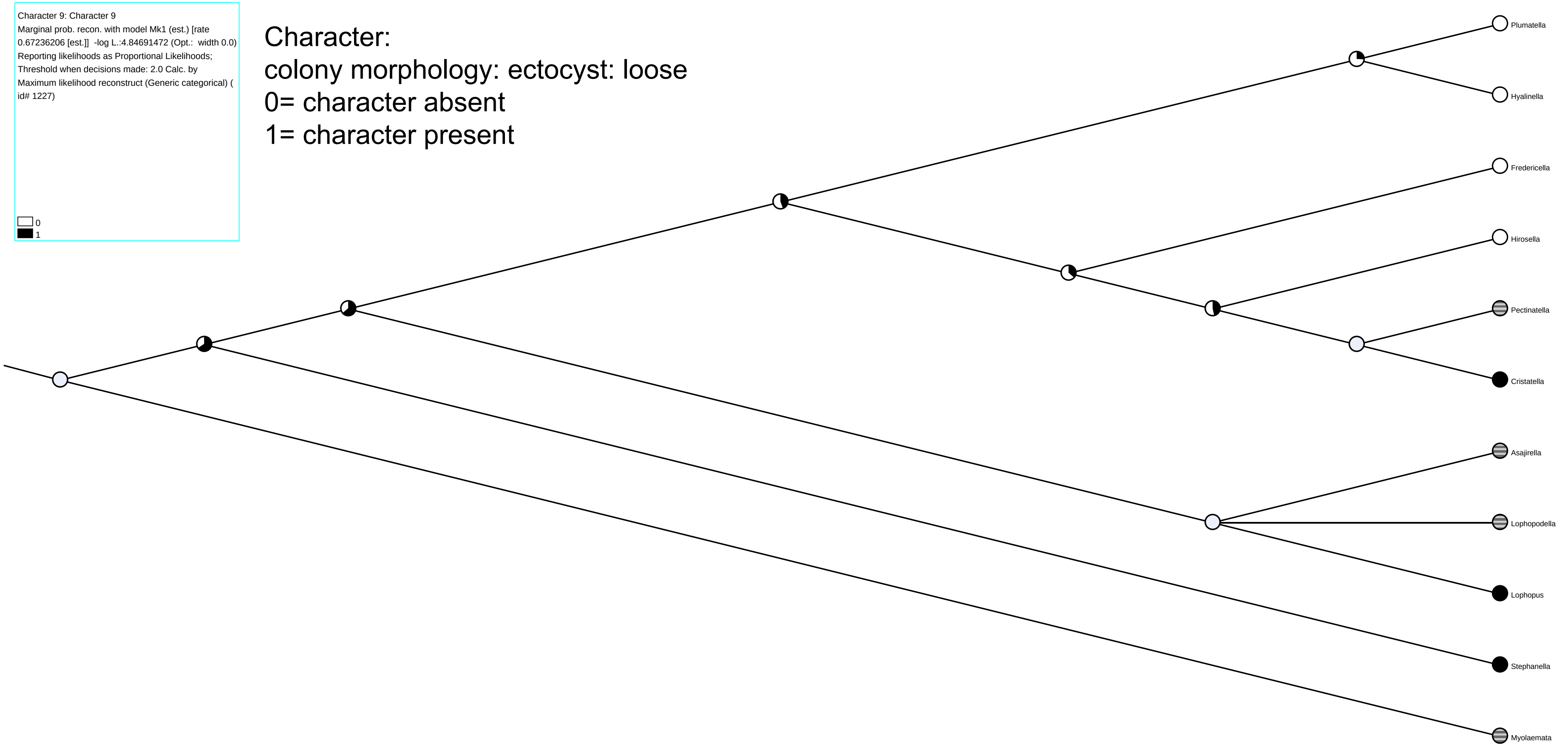

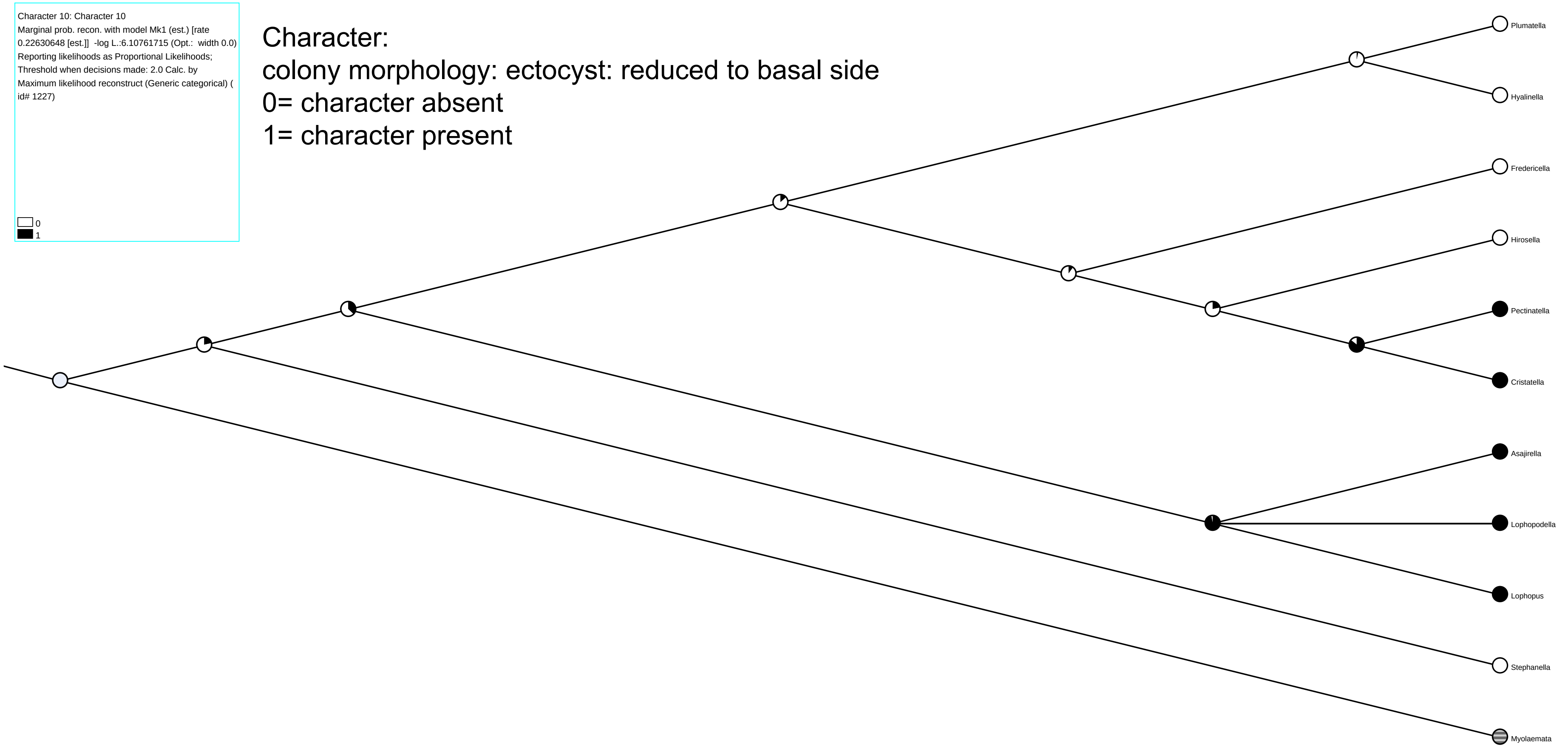
Character 10: Character 10
Marginal prob. recon. with model Mk1 (est.) [rate 0.22630648 [est.]] -log L.:6.10761715 (Opt.: width 0.0)
Reporting likelihoods as Proportional Likelihoods;
Threshold when decisions made: 2.0 Calc. by
Maximum likelihood reconstruct (Generic categorical) (
id# 1227)
0
1
Character:
colony morphology: ectocyst: reduced to basal side
0= character absent
1= character present
Plumatella
Hyalinella
Fredericella
Hirosella
Pectinatella
Cristatella
Asajirella
Lophopodella
Lophopus
Stephanella
Myolaemata

Character 11: Character 11
Marginal prob. recon. with model Mk1 (est.) [rate 0.06538409 [est.]] -log L.:3.76788124 (Opt.: width 0.0) Reporting likelihoods as Proportional Likelihoods; Threshold when decisions made: 2.0 Calc. by Maximum likelihood reconstruct (Generic categorical) (id# 1227)

0
1

Character:
colony morphology: oral budding direction
0= character absent
1= character present

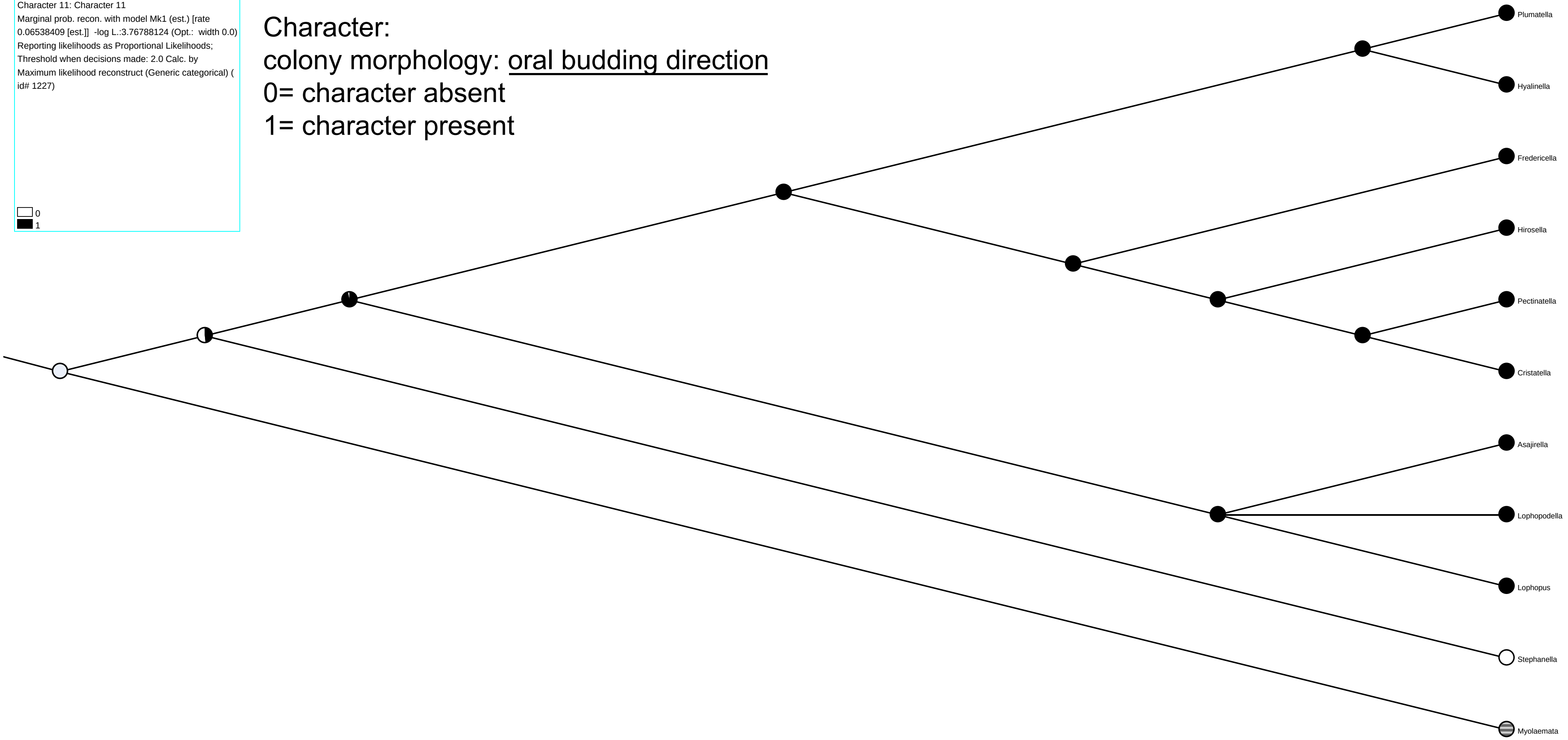

Character:
colony morphology: <u>lateral budding direction</u>
0= character absent
1= character present

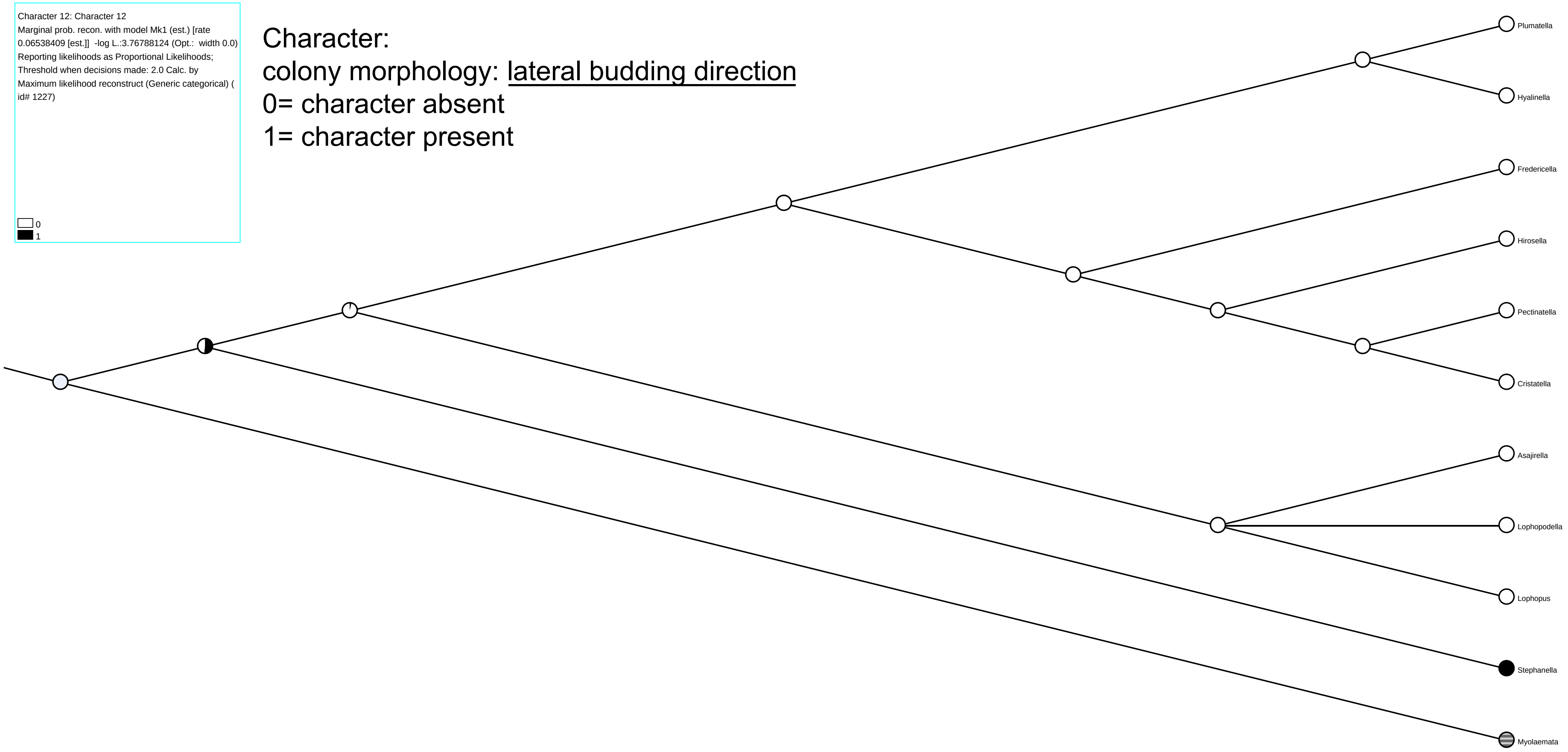

Character 13: Character 13
Categorical data likelihood calculations currently require state distribution to include only contiguous states starting at 0 (e.g., character with only states 2 and 3 not allowed, and should instead be recoded to states 0 and 1). Calculations for one or more characters were not completed. Calc. by Maximum likelihood reconstruct (Generic categorical) (id# 1227)

1

Character:
body wall musculature: circular muscles
0= character absent
1= character present

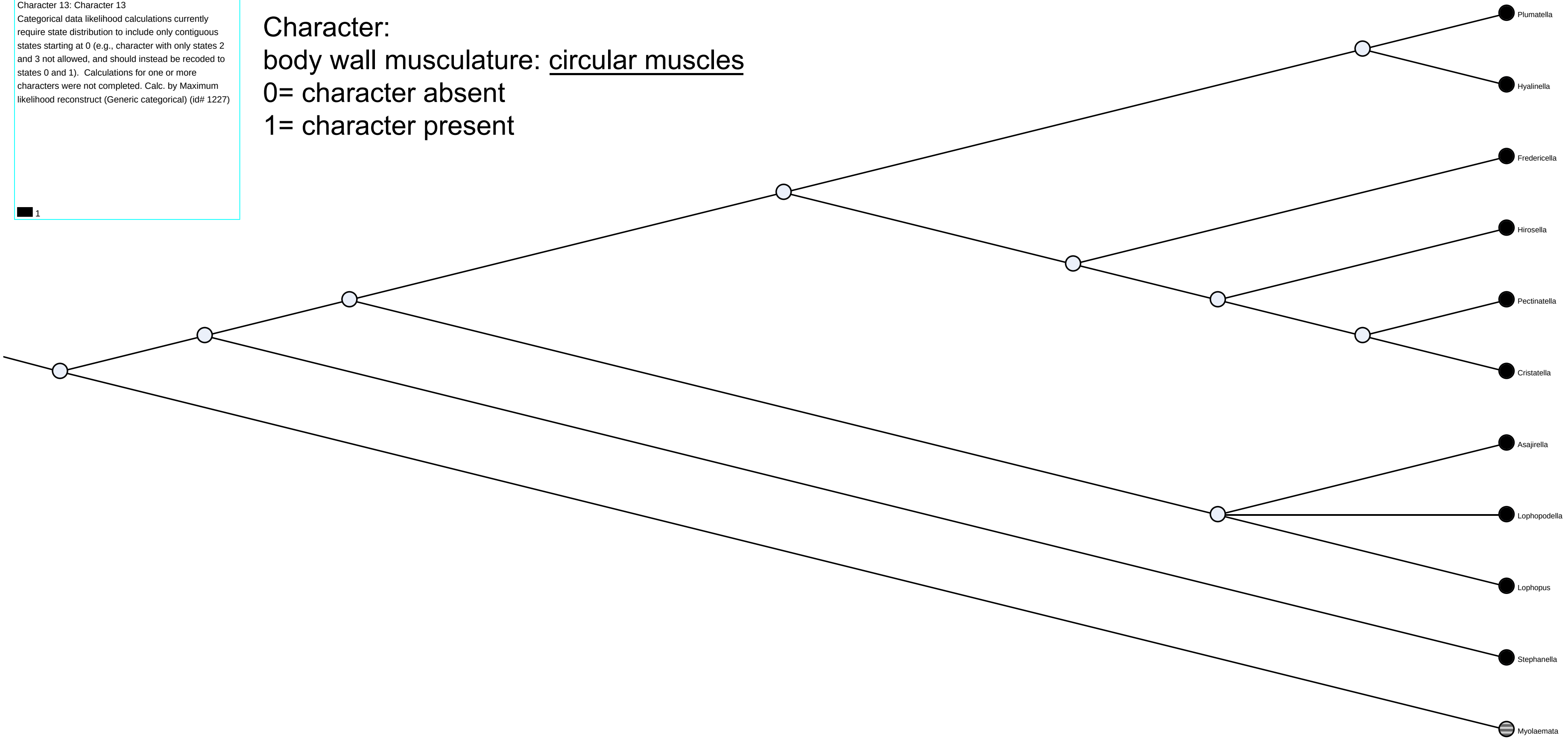

Character 14: Character 14
Categorical data likelihood calculations currently require state distribution to include only contiguous states starting at 0 (e.g., character with only states 2 and 3 not allowed, and should instead be recoded to states 0 and 1). Calculations for one or more characters were not completed. Calc. by Maximum likelihood reconstruct (Generic categorical) (id# 1227)

1

Character:
body wall musculature: longitudinal muscles
0= character absent
1= character present

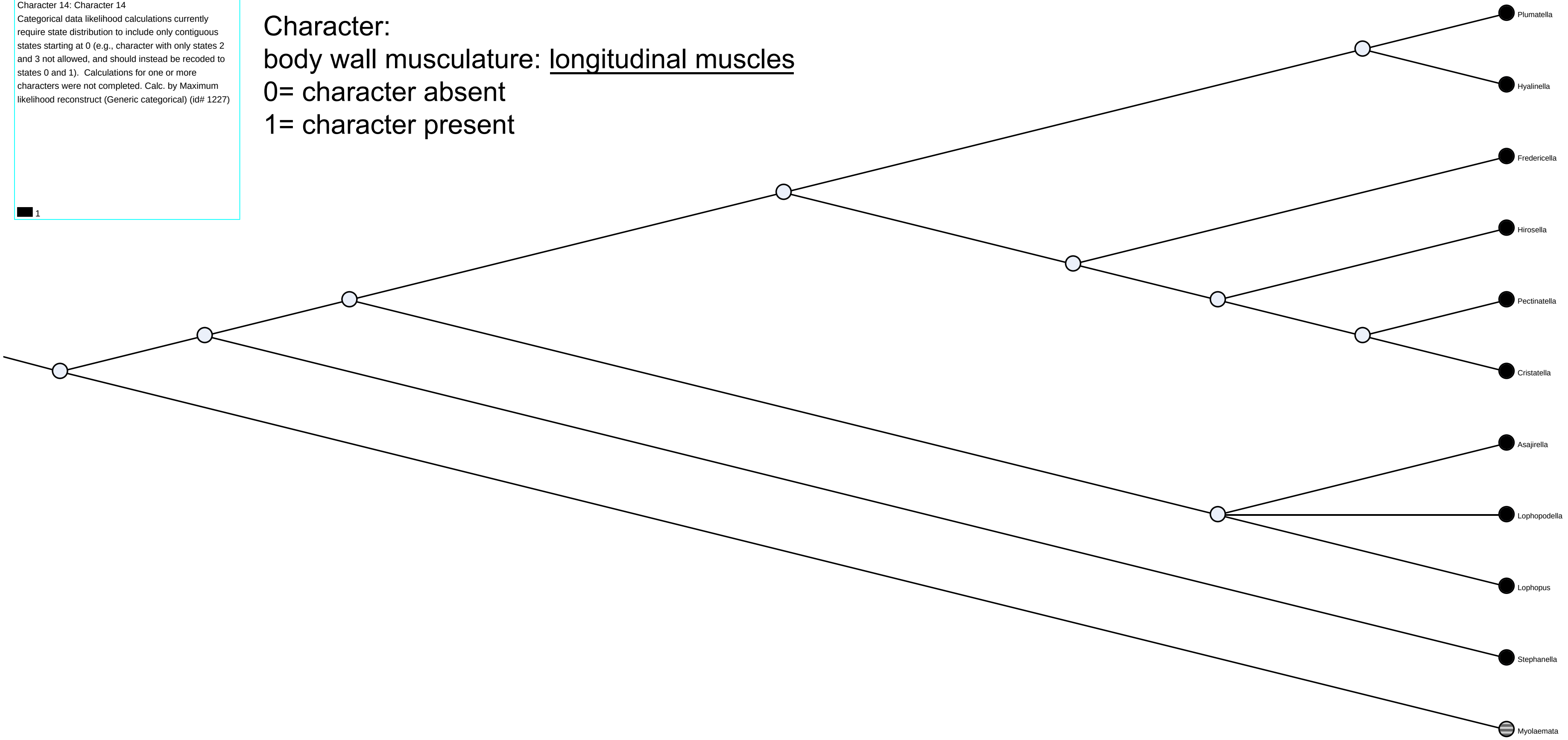

Character:
body wall musculature: diagnonal muscles
0= character absent
1= character present

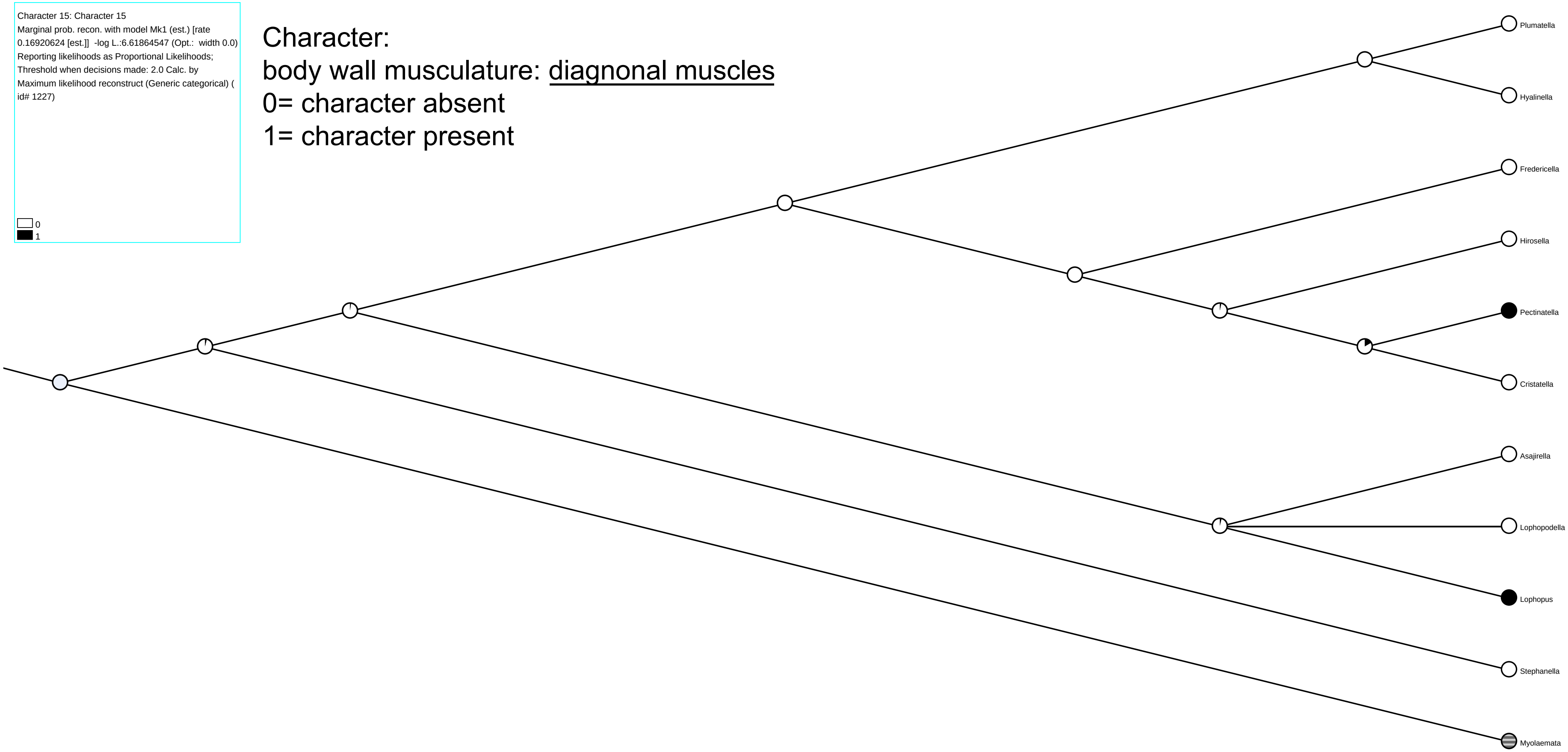

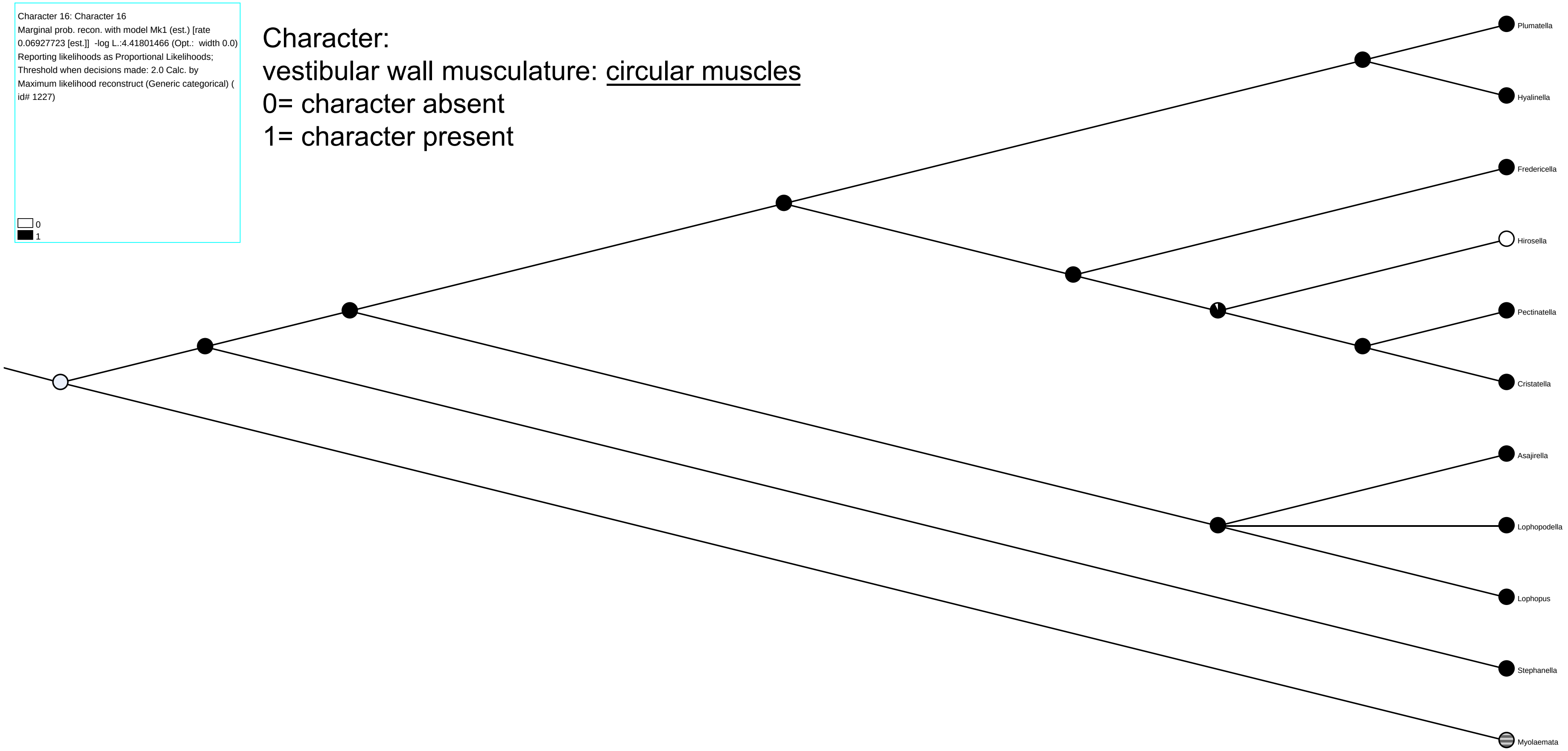
Character 16: Character 16
Marginal prob. recon. with model Mk1 (est.) [rate 0.06927723 [est.]] -log L.:4.41801466 (Opt.: width 0.0) Reporting likelihoods as Proportional Likelihoods; Threshold when decisions made: 2.0 Calc. by Maximum likelihood reconstruct (Generic categorical) (id# 1227)
0
1
Character:
vestibular wall musculature: circular muscles
0= character absent
1= character present
Plumatella
Hyalinella
Fredericella
Hirosella
Pectinatella
Cristatella
Asajirella
Lophopodella
Lophopus
Stephanella
Myolaemata

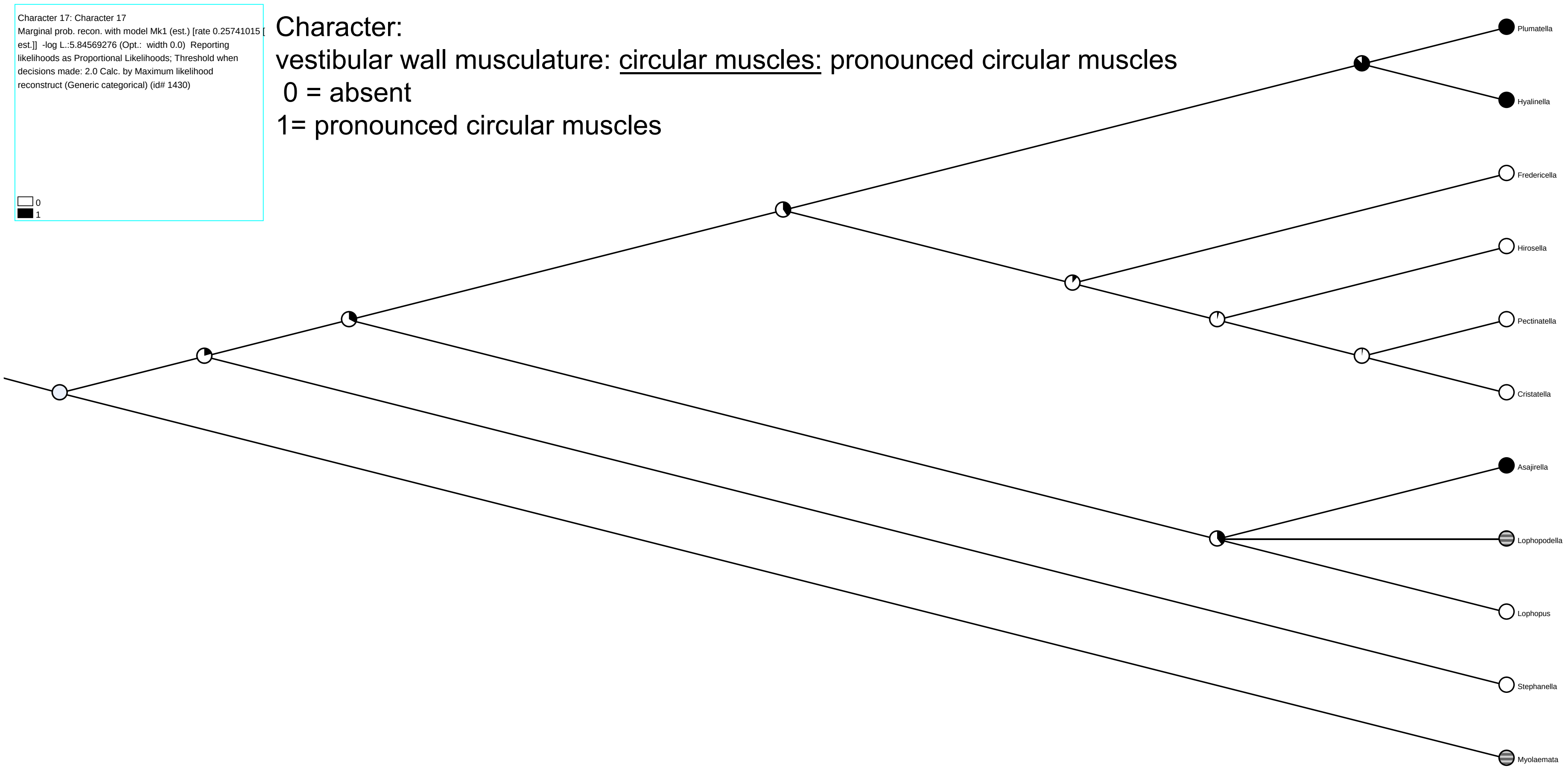

Character 17: Character 17
Marginal prob. recon. with model Mk1 (est.) [rate 0.25741015 [est.]] -log L.:5.84569276 (Opt.: width 0.0) Reporting likelihoods as Proportional Likelihoods; Threshold when decisions made: 2.0 Calc. by Maximum likelihood reconstruct (Generic categorical) (id# 1430)
0
1
Character:
vestibular wall musculature: circular muscles: pronounced circular muscles
0 = absent
1= pronounced circular muscles
Plumatella
Hyalinella
Fredericella
Hirosella
Pectinatella
Cristatella
Asajirella
Lophopodella
Lophopus
Stephanella
Myolaemata

Character 18: Character 18
Categorical data likelihood calculations currently require state distribution to include only contiguous states starting at 0 (e.g., character with only states 2 and 3 not allowed, and should instead be recoded to states 0 and 1). Calculations for one or more characters were not completed. Calc. by Maximum likelihood reconstruct (Generic categorical) (id# 1227)

■ 1

Character:
vestibular wall musculature: longitudinal muscles
0= character absent
1= character present

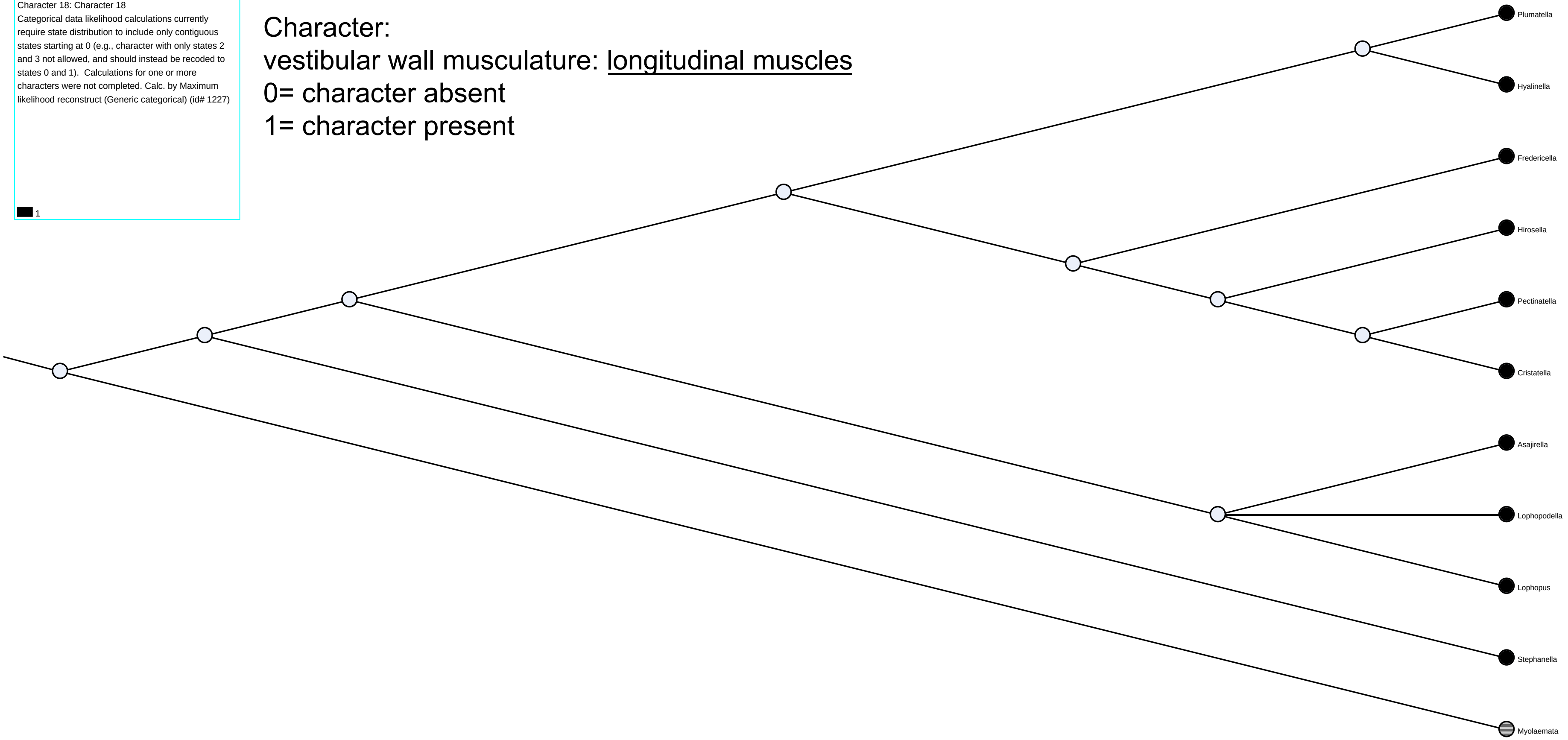

Character 19: Character 19
Marginal prob. recon. with model Mk1 (est.) [rate 0.07906257 [est.]] -log L.:4.28598709 (Opt.: width 0.0) Reporting likelihoods as Proportional Likelihoods; Threshold when decisions made: 2.0 Calc. by Maximum likelihood reconstruct (Generic categorical) (id# 1430)

0
1

Character:
vestibular wall musculature: longitudinal muscles: pronounced longitudinal muscles
0 = absent
1 = pronounced longitudinal muscles

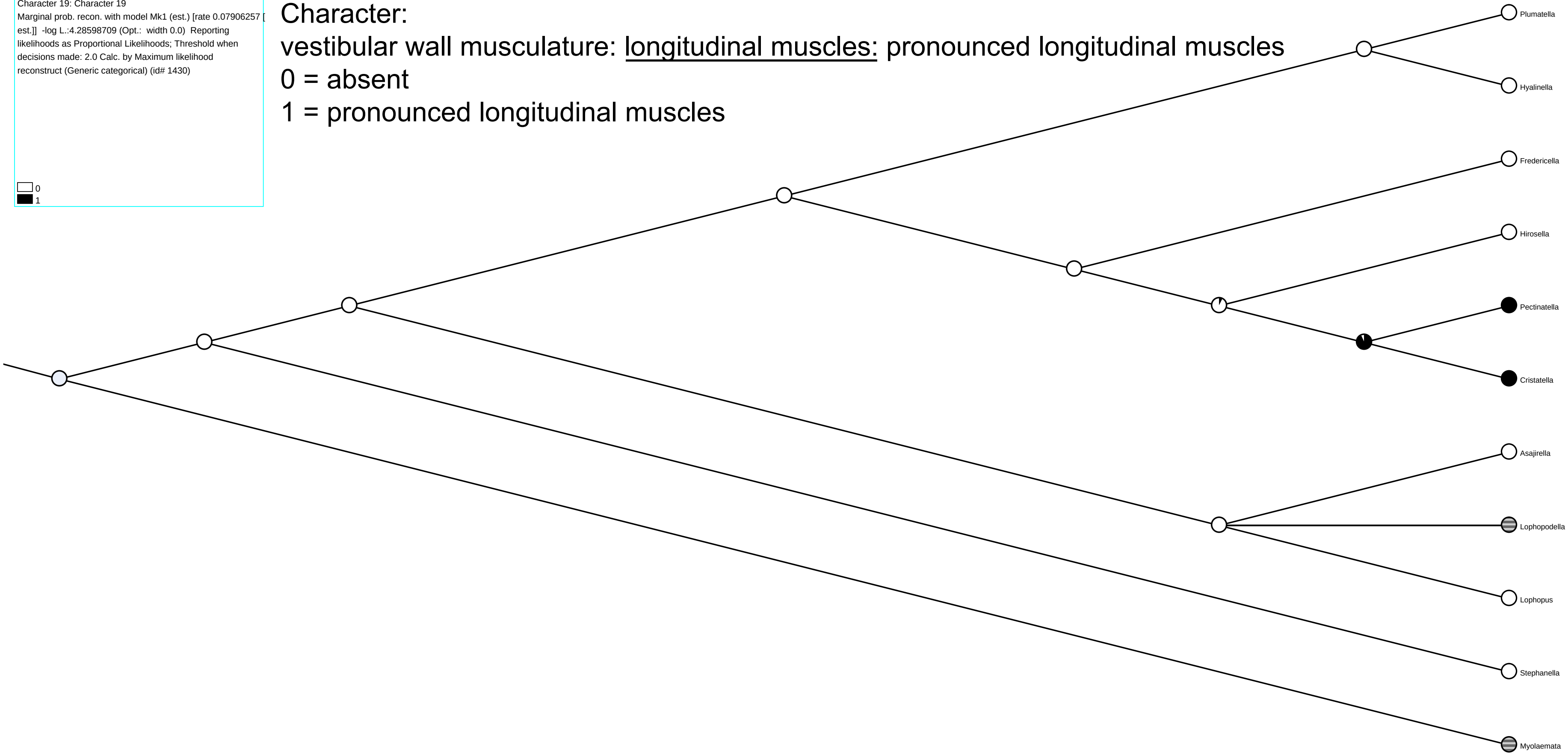

Character 20: Character 20
Marginal prob. recon. with model Mk1 (est.) [rate 0.07050939 [est.]] -log L.:3.69979753 (Opt.: width 0.0) Reporting likelihoods as Proportional Likelihoods; Threshold when decisions made: 2.0 Calc. by Maximum likelihood reconstruct (Generic categorical) (id# 1430)

0
1

Character:
vestibular wall musculature: diaphragmatic sphincter muscle
0 = undifferentiated
1= present

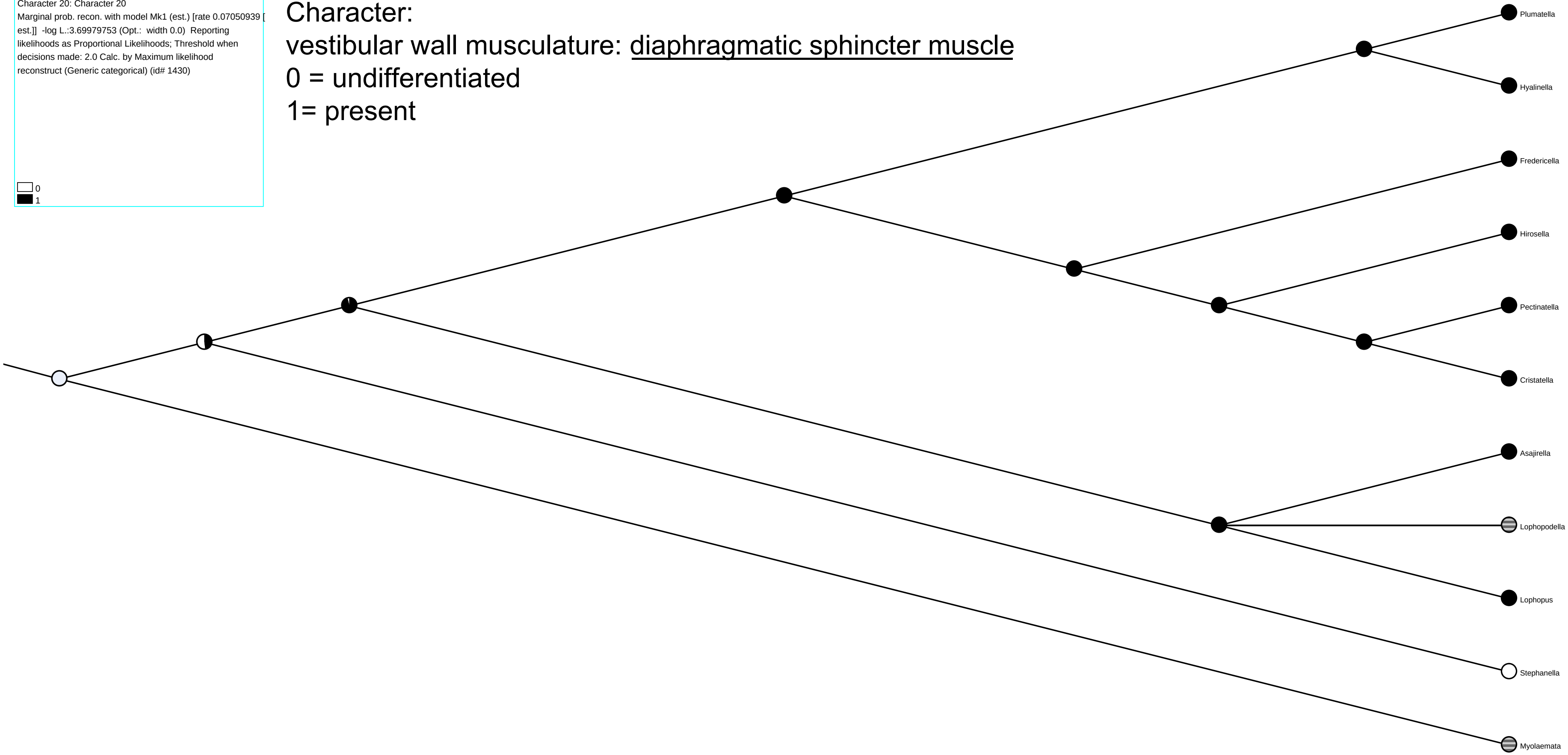

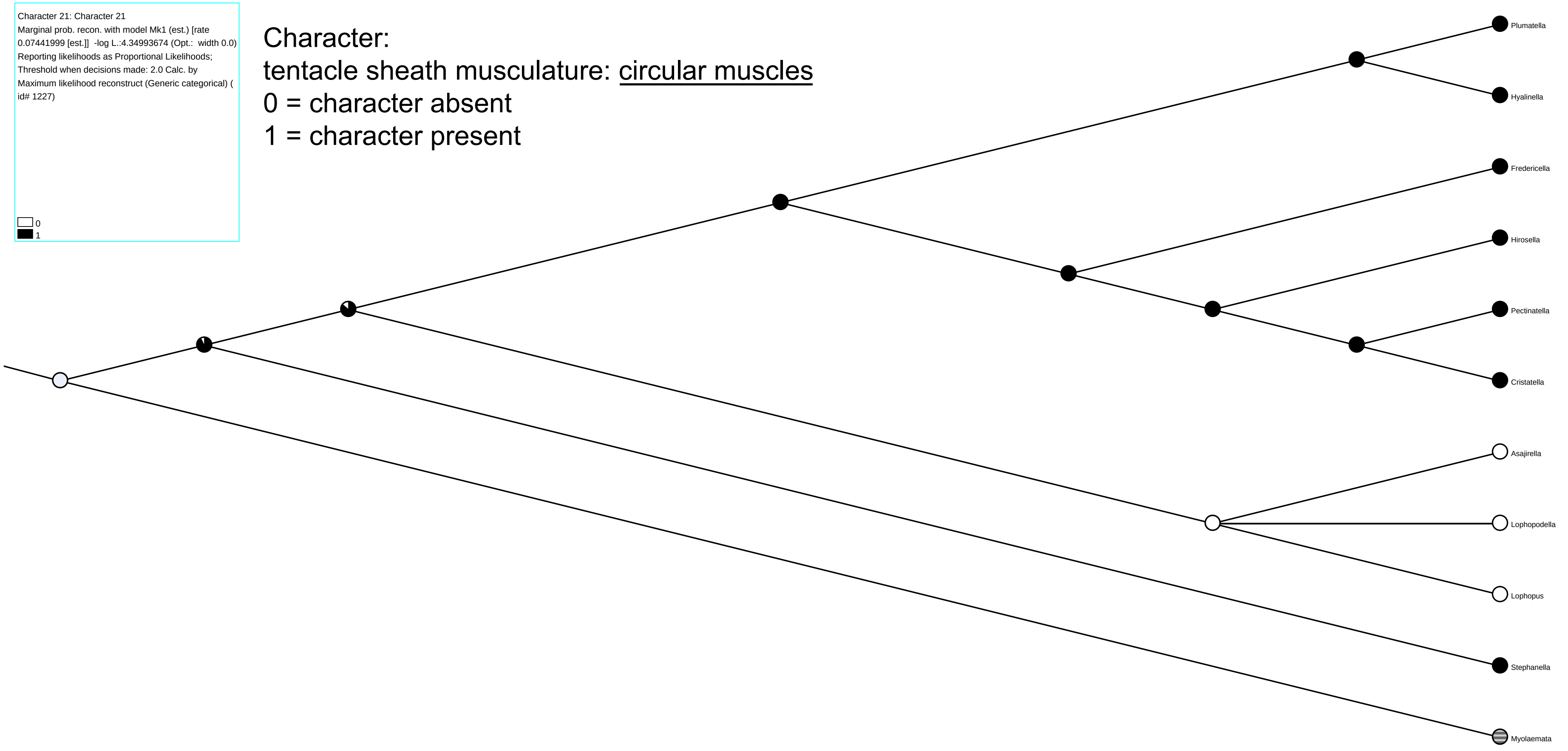
Character 21: Character 21
Marginal prob. recon. with model Mk1 (est.) [rate 0.07441999 [est.]] -log L.:4.34993674 (Opt.: width 0.0) Reporting likelihoods as Proportional Likelihoods; Threshold when decisions made: 2.0 Calc. by Maximum likelihood reconstruct (Generic categorical) (id# 1227)
0
1
Character:
tentacle sheath musculature: circular muscles
0 = character absent
1 = character present
Plumatella
Hyalinella
Fredericella
Hirosella
Pectinatella
Cristatella
Asajirella
Lophopodella
Lophopus
Stephanella
Myolaemata

Character 22: Character 22
Categorical data likelihood calculations currently require state distribution to include only contiguous states starting at 0 (e.g., character with only states 2 and 3 not allowed, and should instead be recoded to states 0 and 1). Calculations for one or more characters were not completed. Calc. by Maximum likelihood reconstruct (Generic categorical) (id# 1430)

Character:
tentacle sheath musculature: circular muscles: abberations
A = pronounced circular muscles in tentacle sheath
D = circular muscles restricted to basal region
0 = present

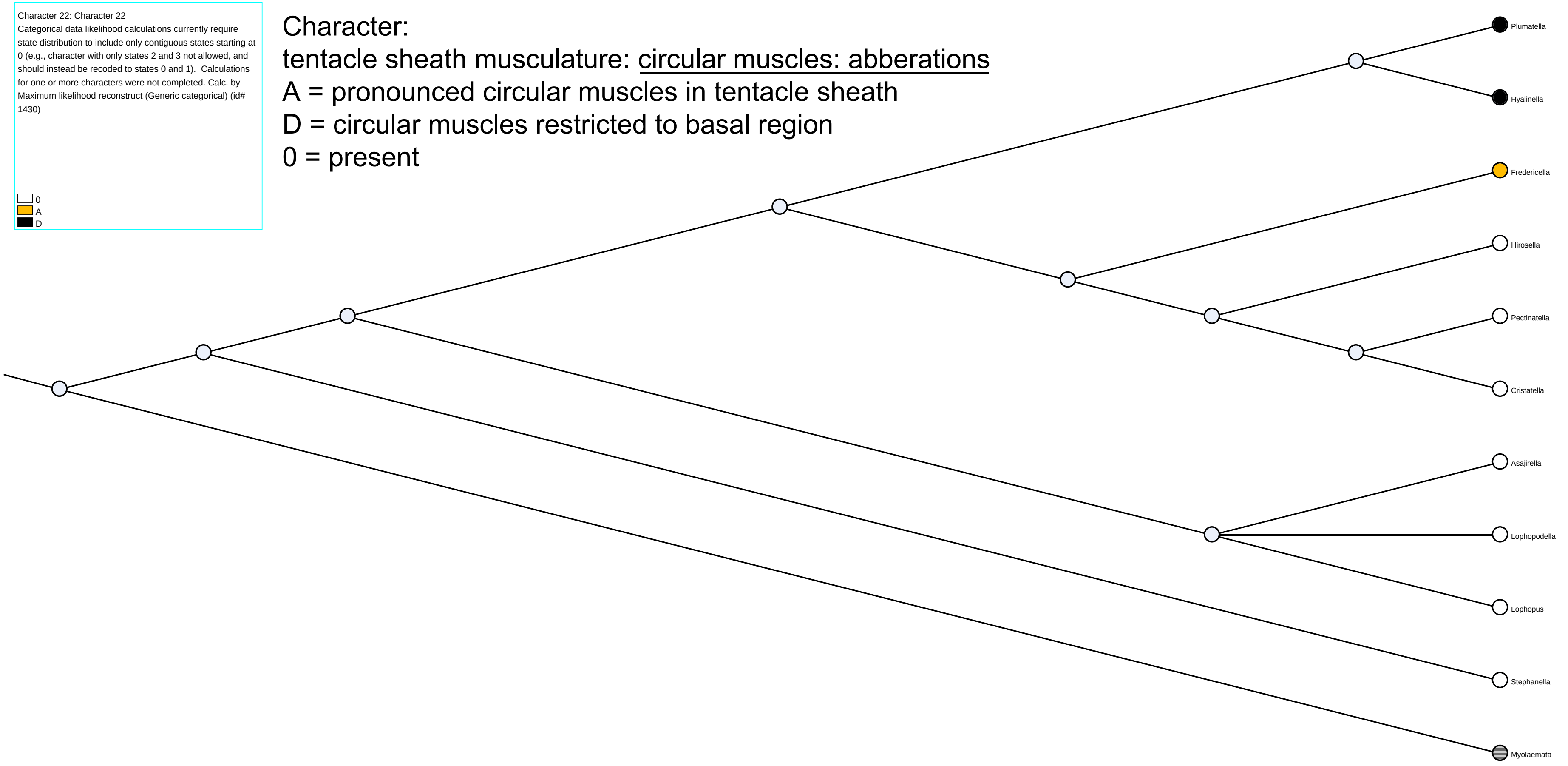

Character 23: Character 23
Categorical data likelihood calculations currently require state distribution to include only contiguous states starting at 0 (e.g., character with only states 2 and 3 not allowed, and should instead be recoded to states 0 and 1). Calculations for one or more characters were not completed. Calc. by Maximum likelihood reconstruct (Generic categorical) (id# 1227)

1

Character:
tentacle sheath musculature: longitudinal muscles
0 = character absent
1 = character present

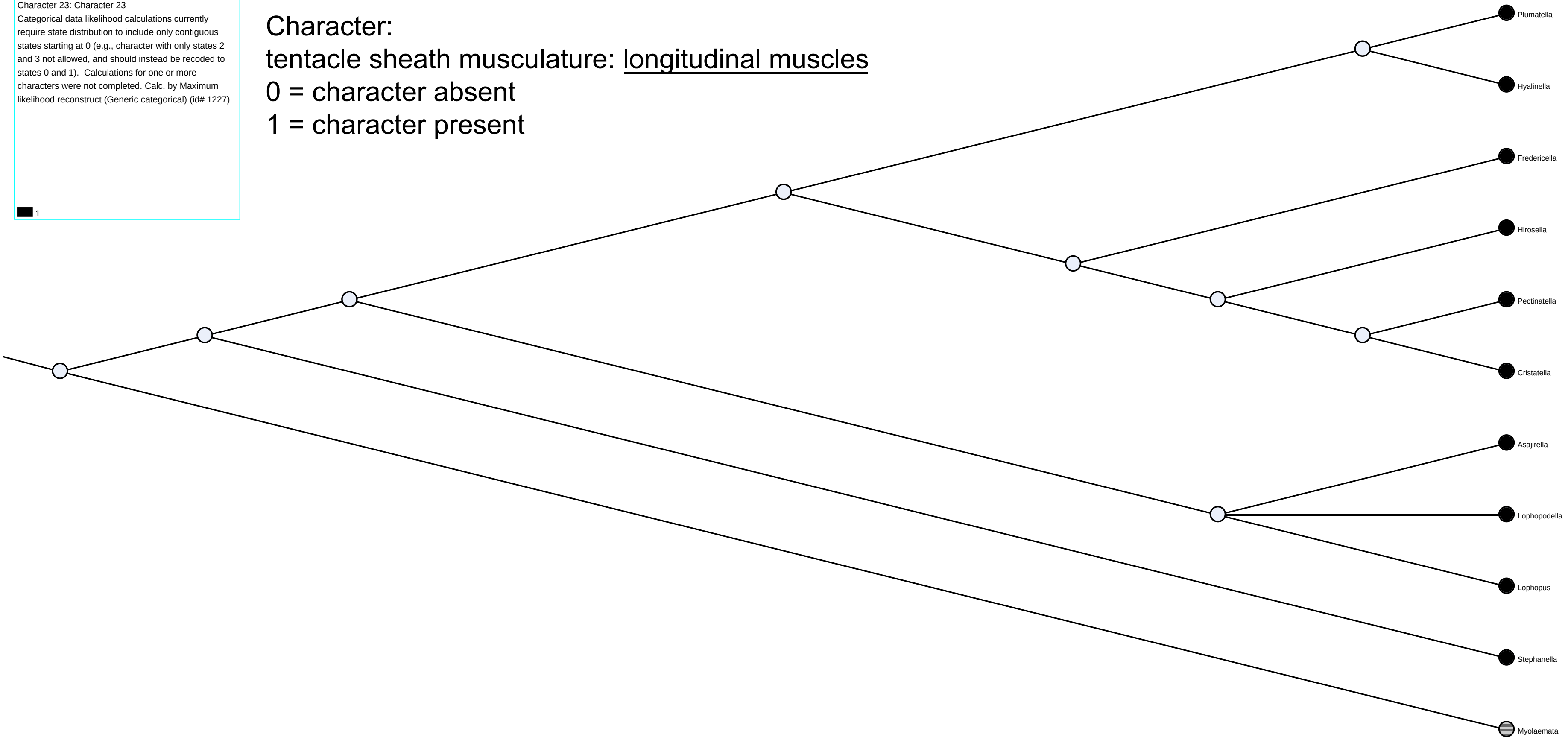

Character 24: Character 24
Marginal prob. recon. with model Mk1 (est.) [rate 0.16451055 [est.]] -log L.:5.89685542 (Opt.: width 0.0) Reporting likelihoods as Proportional Likelihoods; Threshold when decisions made: 2.0 Calc. by Maximum likelihood reconstruct (Generic categorical) (id# 1430)

0
1

Character:
tentacle sheath musculature: <u>longitudinal muscles: abberations</u>
1 = Pronounced longitudinal muscles in the tentacle sheath
0 = no abberation

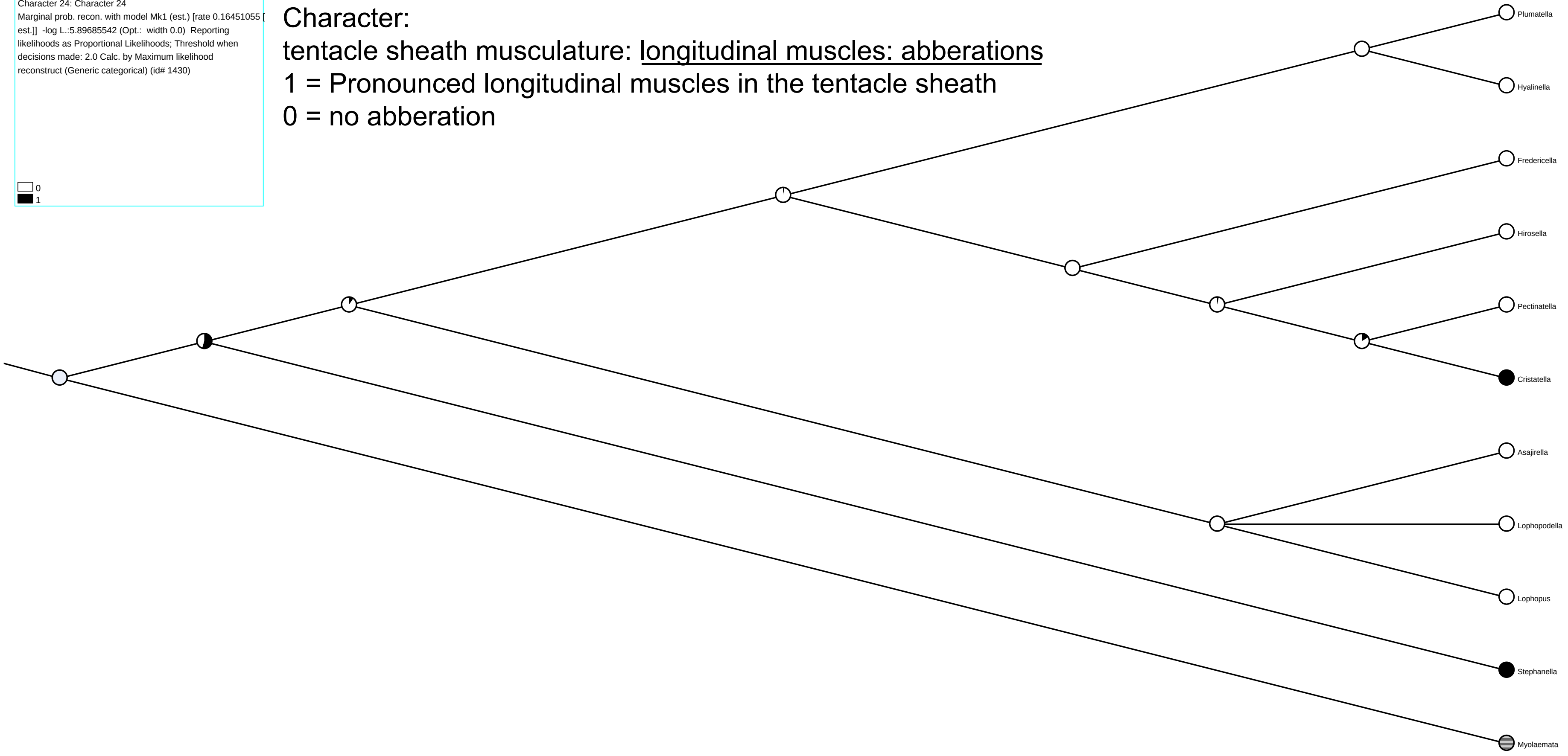

Character 25: Character 25
Categorical data likelihood calculations currently require state distribution to include only contiguous states starting at 0 (e.g., character with only states 2 and 3 not allowed, and should instead be recoded to states 0 and 1). Calculations for one or more characters were not completed. Calc. by Maximum likelihood reconstruct (Generic categorical) (id# 1227)

1

Character:
apertual musculature: <u>vestibular dilatators</u>
0 = character absent
1 = character present

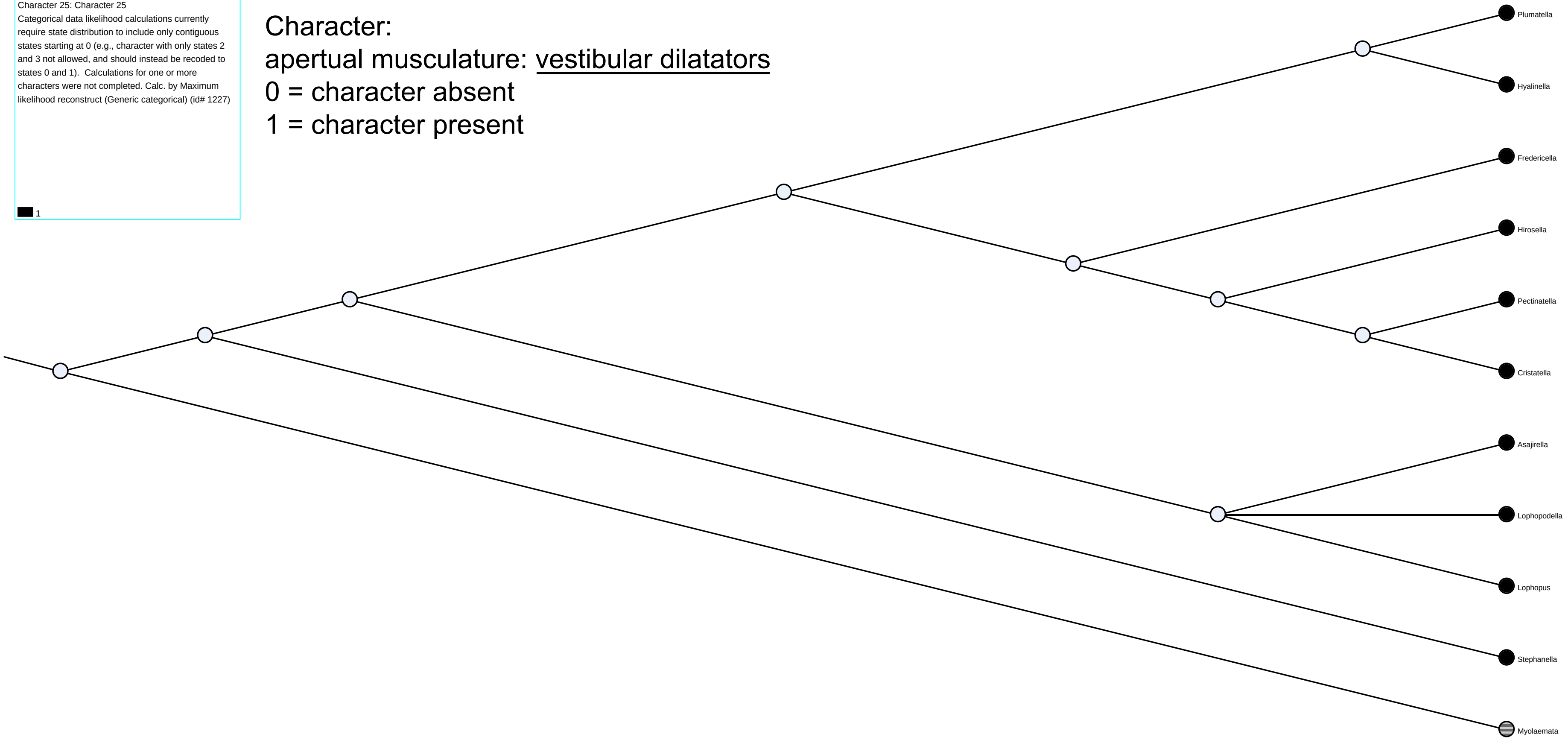

Character 26: Character 26
Categorical data likelihood calculations currently require state distribution to include only contiguous states starting at 0 (e.g., character with only states 2 and 3 not allowed, and should instead be recoded to states 0 and 1). Calculations for one or more characters were not completed. Calc. by Maximum likelihood reconstruct (Generic categorical) (id# 1227)

Character:
apertual musculature: duplicature bands
1 = character present

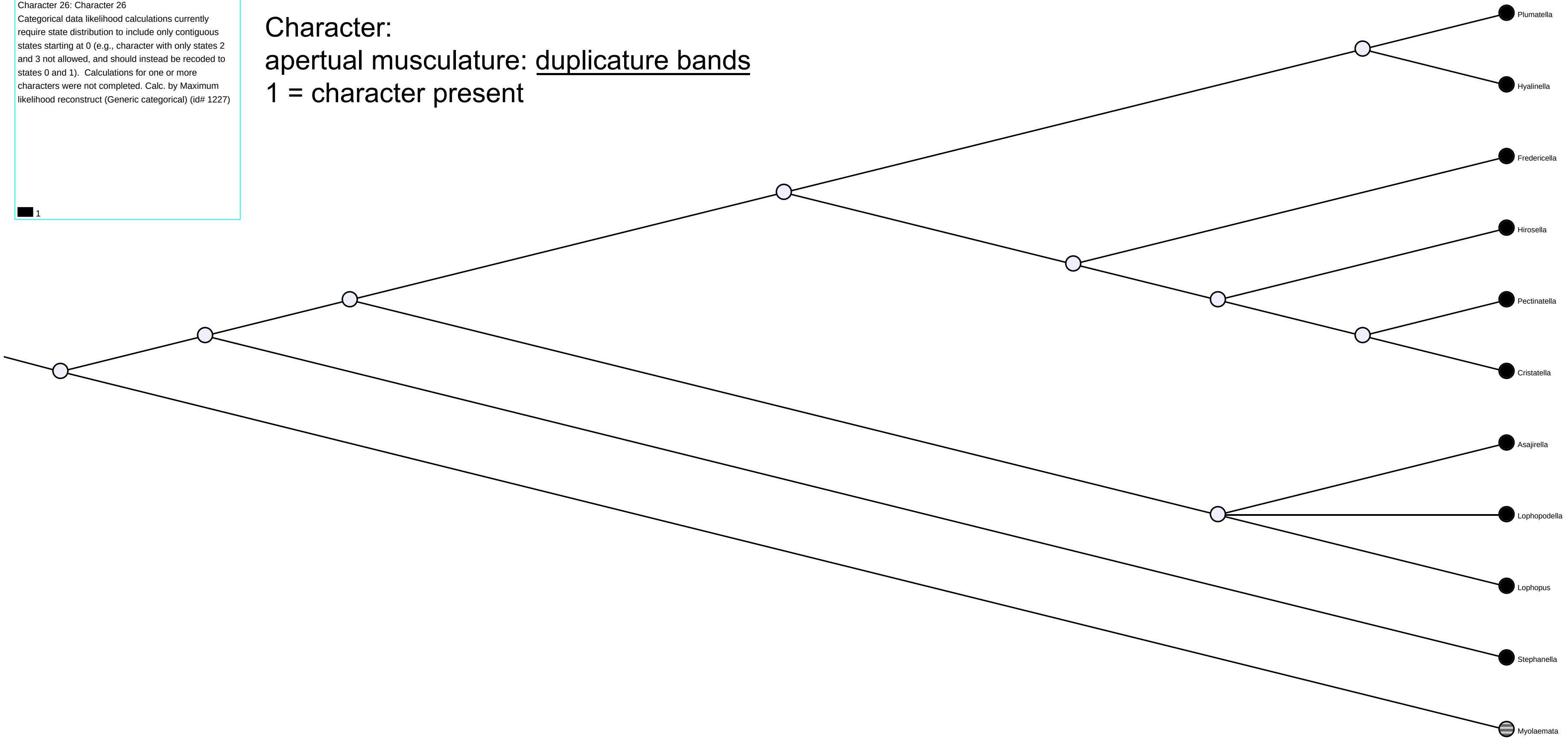

Character 27: Character 27
Categorical data likelihood calculations currently require state distribution to include only contiguous states starting at 0 (e.g., character with only states 2 and 3 not allowed, and should instead be recoded to states 0 and 1). Calculations for one or more characters were not completed. Calc. by Maximum likelihood reconstruct (Generic categorical) (id# 1227)

E
F

Character:
apertual musculature: duplicature bands: attachment location
E = attach to body wall + tentacle sheath
F = attach to body wall + diaphragmatic sphincter

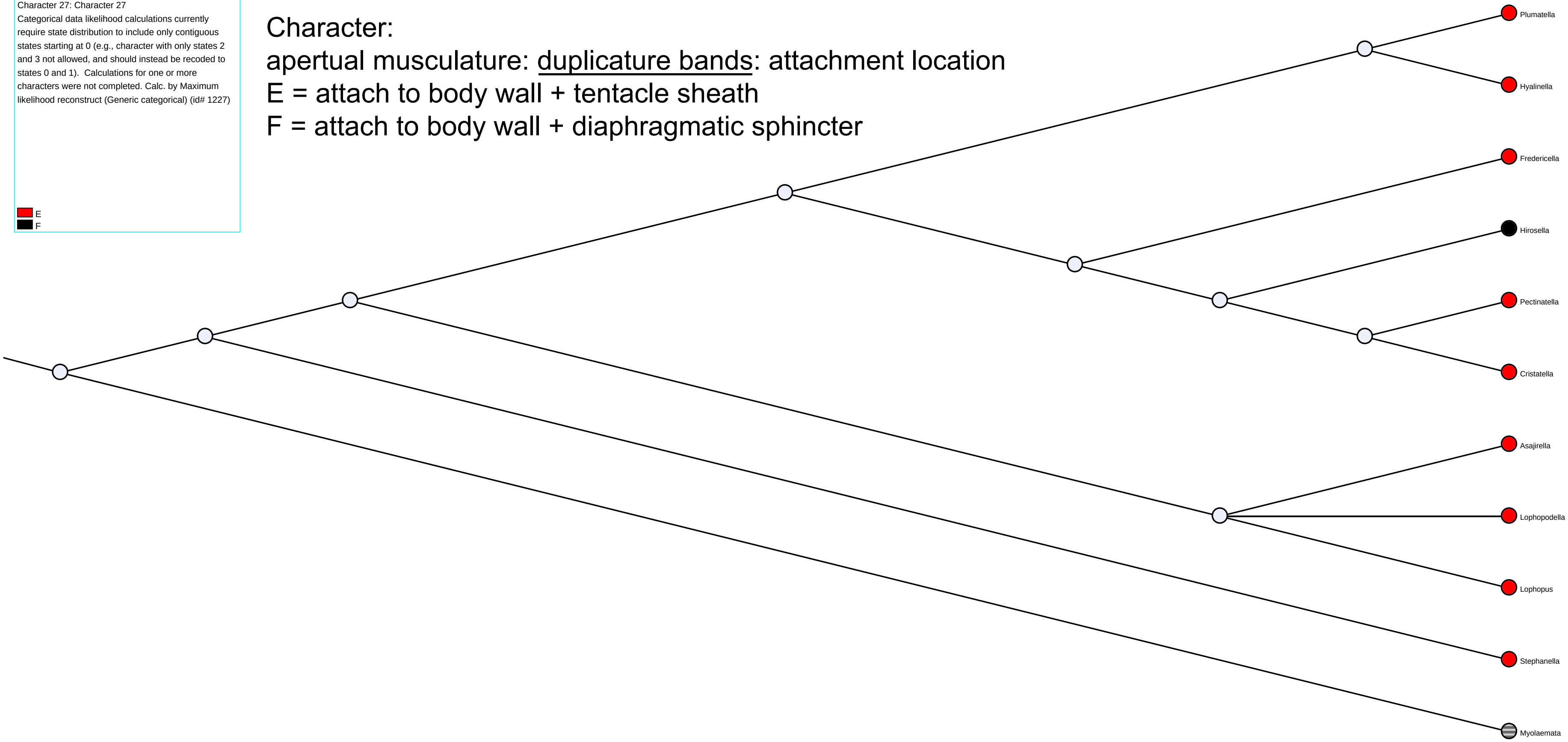

Character 28: Character 28
Marginal prob. recon. with model Mk1 (est.) [rate 0.07441999 [est.]] -log L.:4.34993674 (Opt.: width 0.0) Reporting likelihoods as Proportional Likelihoods; Threshold when decisions made: 2.0 Calc. by Maximum likelihood reconstruct (Generic categorical) (id# 1430)

0
1

Character: epistome: shape
0 = dome shaped
1 = flap like

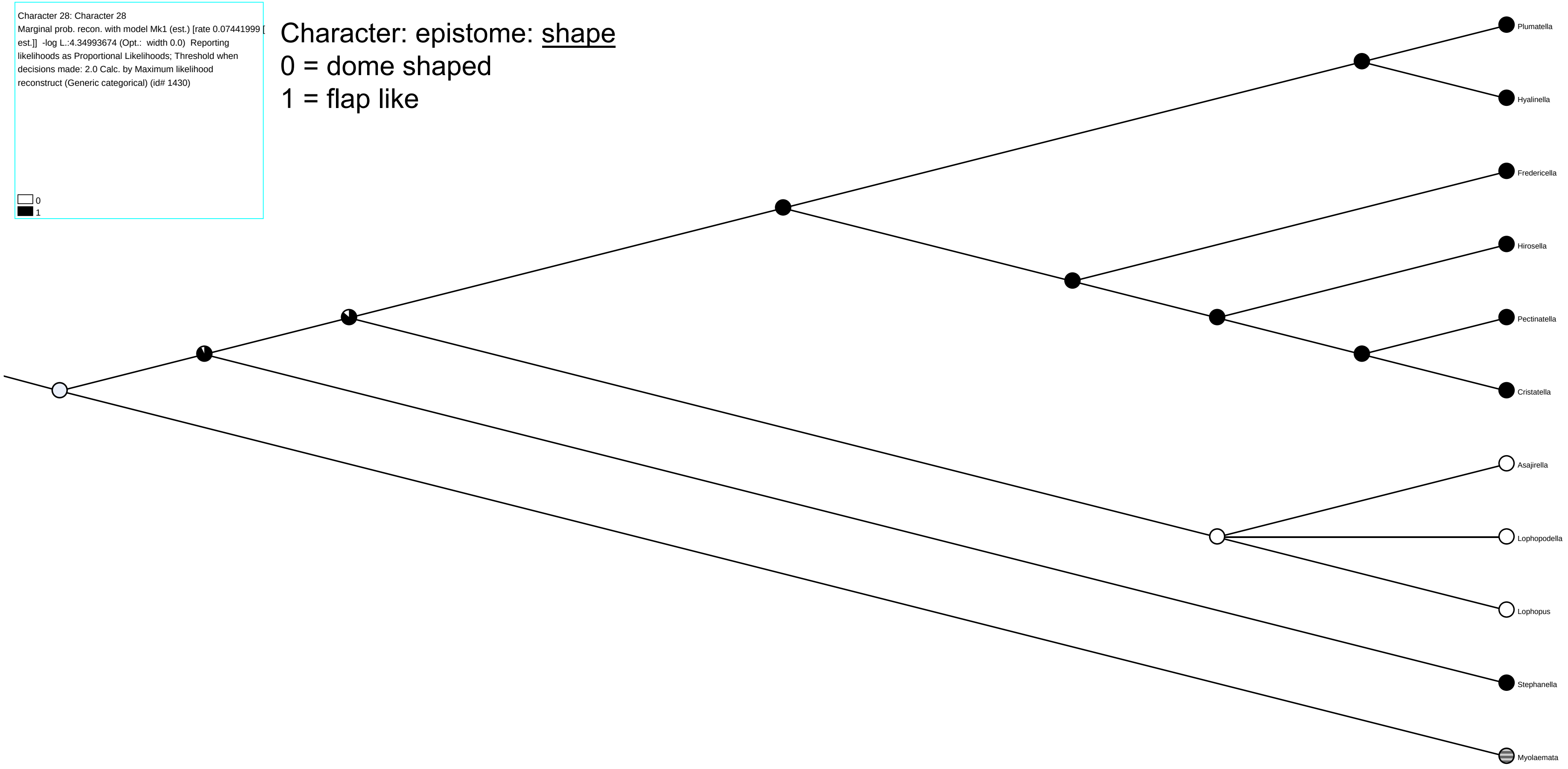

Character 29: Character 29
Marginal prob. recon. with model Mk1 (est.) [rate 0.06855384 [est.]] -log L.:4.42273006 (Opt.: width 0.0)
Reporting likelihoods as Proportional Likelihoods;
Threshold when decisions made: 2.0 Calc. by
Maximum likelihood reconstruct (Generic categorical) (id# 1227)

Character:
epistome: musculature basket
0 = character absent
1 = character present

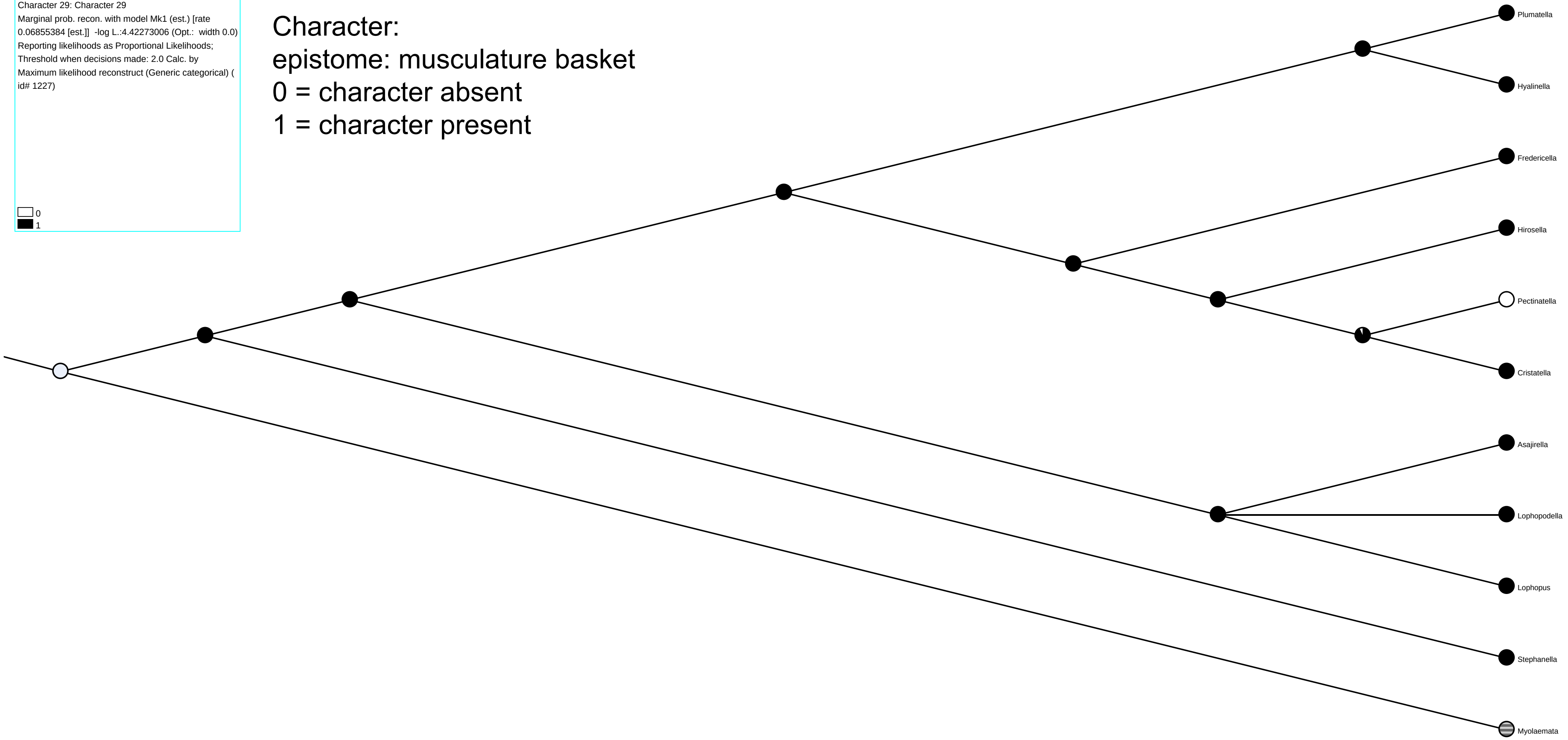

Character 30: Character 30
Marginal prob. recon. with model Mk1 (est.) [rate 0.61721812 [est.]] -log L.:6.89470057 (Opt.: width 0.0) Reporting likelihoods as Proportional Likelihoods; Threshold when decisions made: 2.0 Calc. by Maximum likelihood reconstruct (Generic categorical) (id# 1227)

0
1

Character:
epistome: musculature transversal
0 = character absent
1 = character present

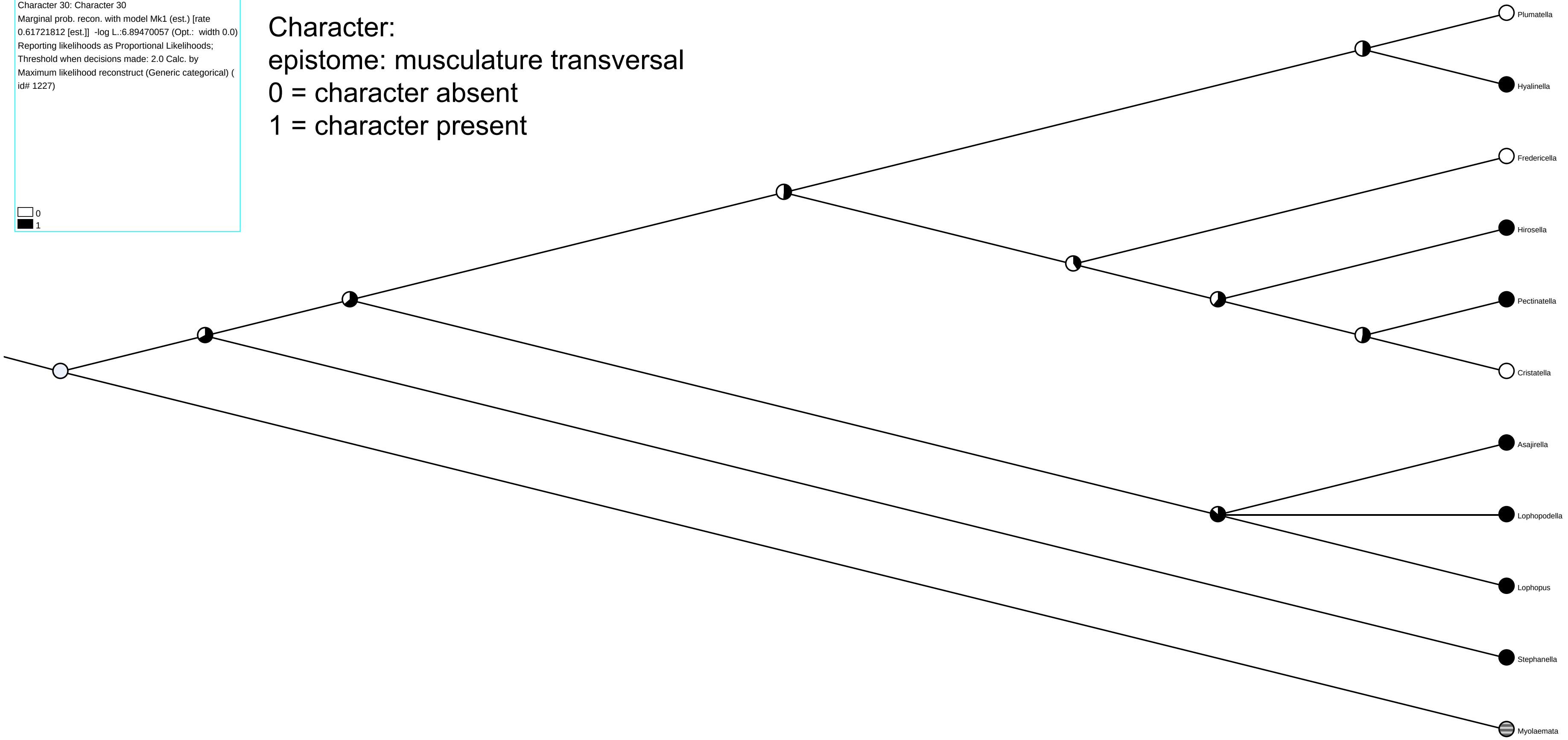

Character 31: Character 31
Categorical data likelihood calculations currently require state distribution to include only contiguous states starting at 0 (e.g., character with only states 2 and 3 not allowed, and should instead be recoded to states 0 and 1). Calculations for one or more characters were not completed. Calc. by Maximum likelihood reconstruct (Generic categorical) (id# 1227)

A
B
I
J

Character:
lophophore muscles: lophophoral arm muscle
A = pronounced
B = present
I = delicate
J = absent

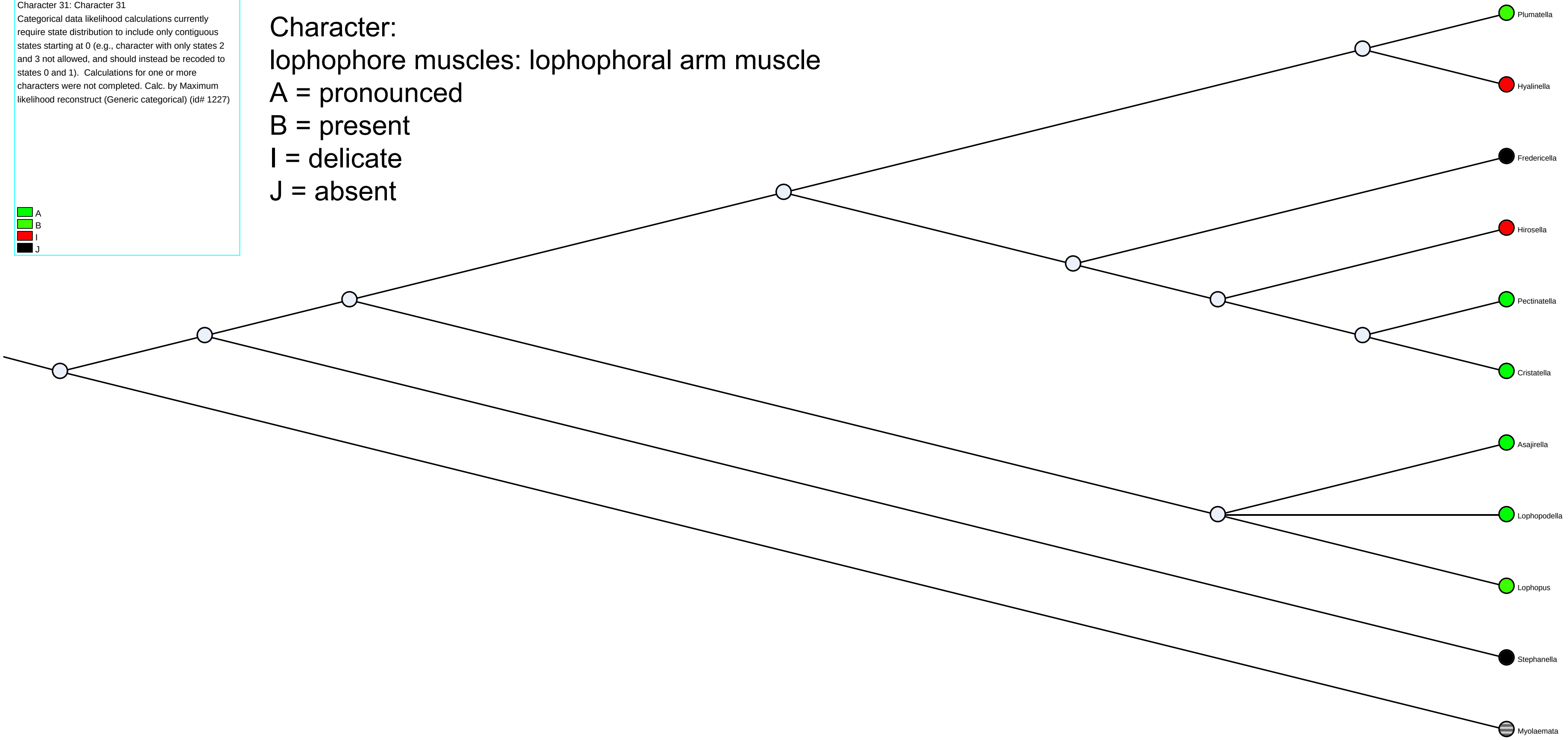

Character 32: Character 32
Marginal prob. recon. with model Mk1 (est.) [rate 0.15858967 [est.]] -log L.:5.96228763 (Opt.: width 0.0)
Reporting likelihoods as Proportional Likelihoods; Threshold when decisions made: 2.0 Calc. by Maximum likelihood reconstruct (Generic categorical) (id# 1227)

0
1

Character:
lophophoral muscles: circum pharyngeal muscle
0 = character absent
1 = character present

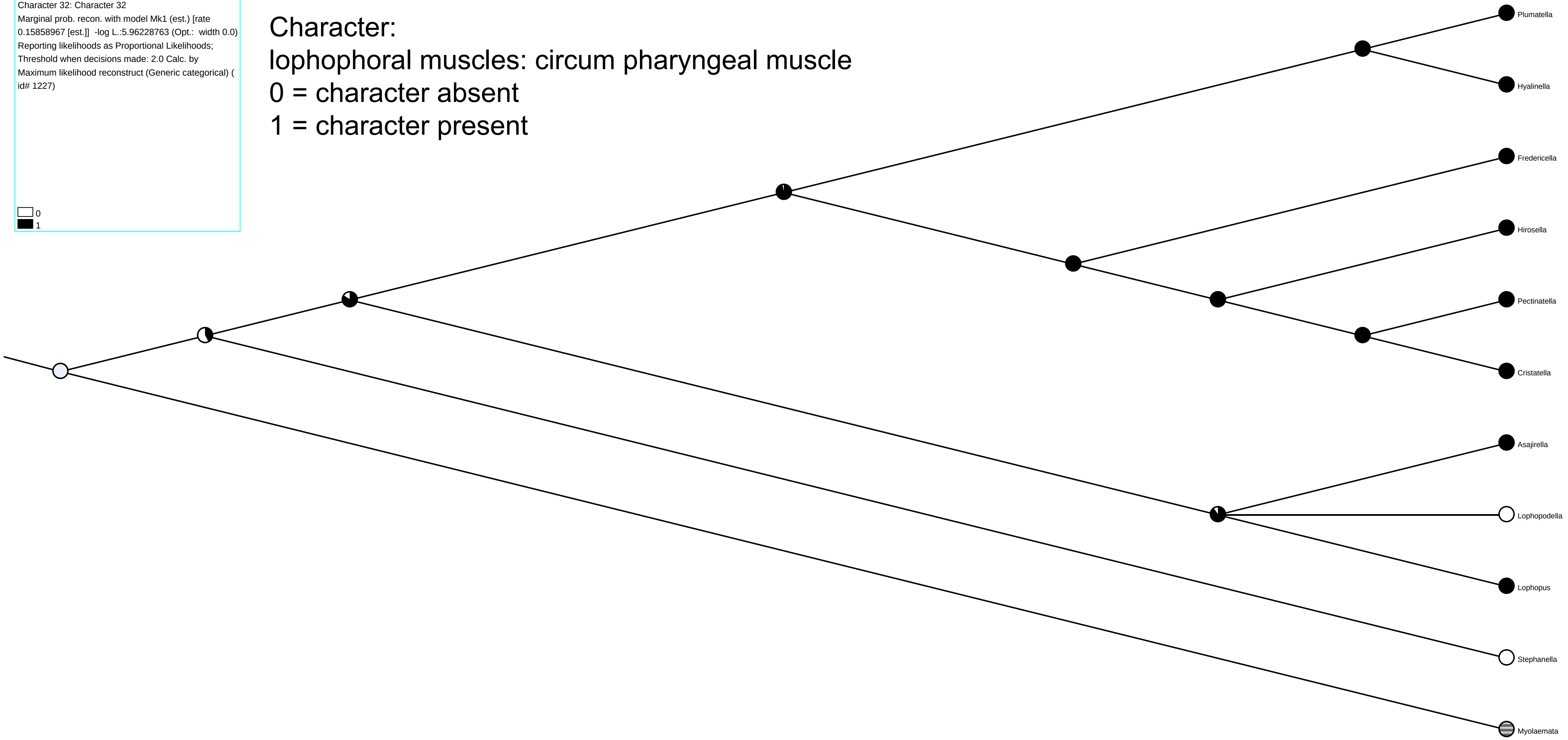

Character 33: Character 33
Marginal prob. recon. with model Mk1 (est.) [rate 0.22132713 [est.]] -log L.:6.04245663 (Opt.: width 0.0) Reporting likelihoods as Proportional Likelihoods; Threshold when decisions made: 2.0 Calc. by Maximum likelihood reconstruct (Generic categorical) (id# 1227)

Character:
lophophoral muscles: ring canal muscles
0 = character absent
1 = character present

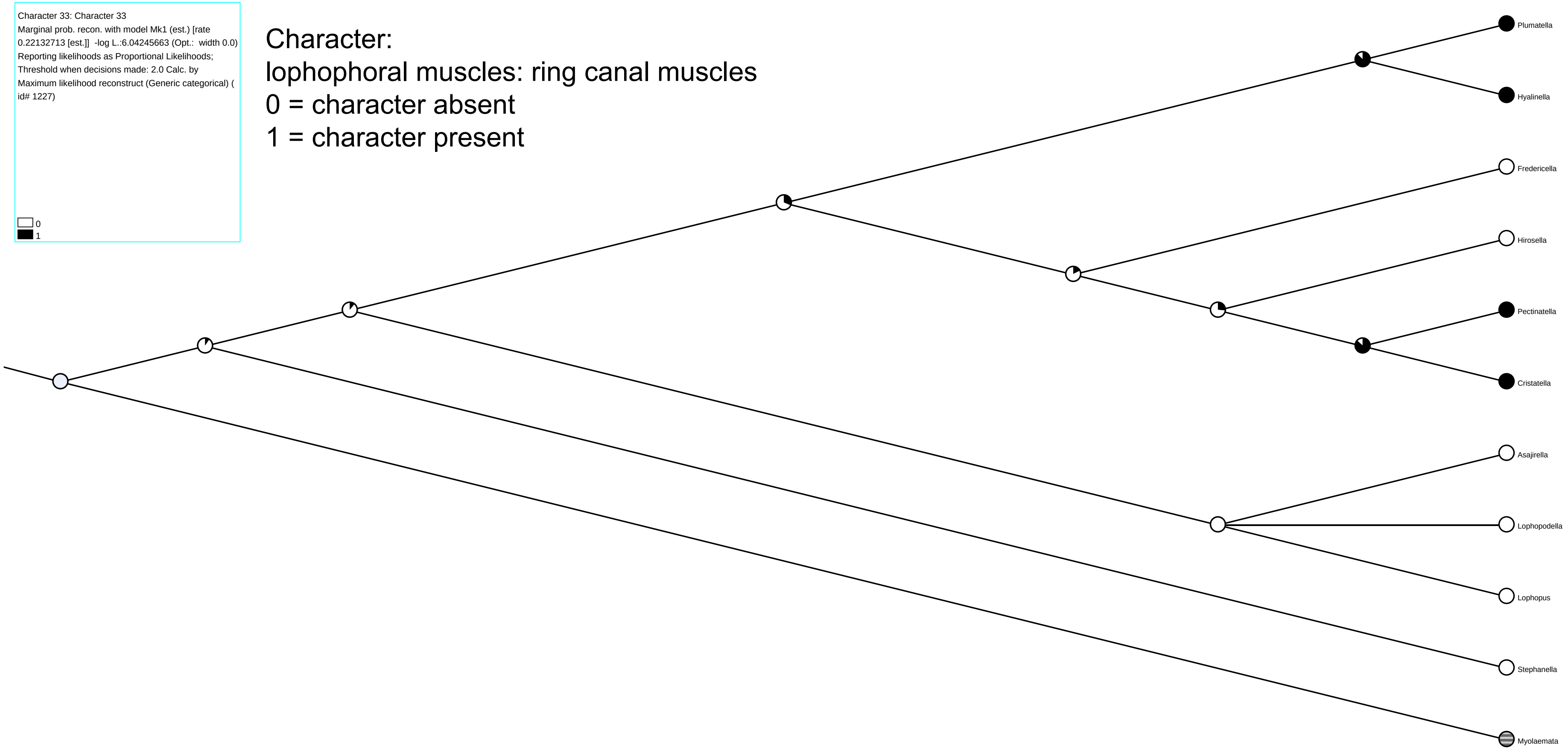

Character 34: Character 34
Marginal prob. recon. with model Mk1 (est.) [rate 0.06538409 [est.]] -log L.:3.76788124 (Opt.: width 0.0)
Reporting likelihoods as Proportional Likelihoods;
Threshold when decisions made: 2.0 Calc. by Maximum likelihood reconstruct (Generic categorical) (id# 1227)

0
1

Character:
abfrontal tentacle muscles: median bands
0 = character absent
1 = character present

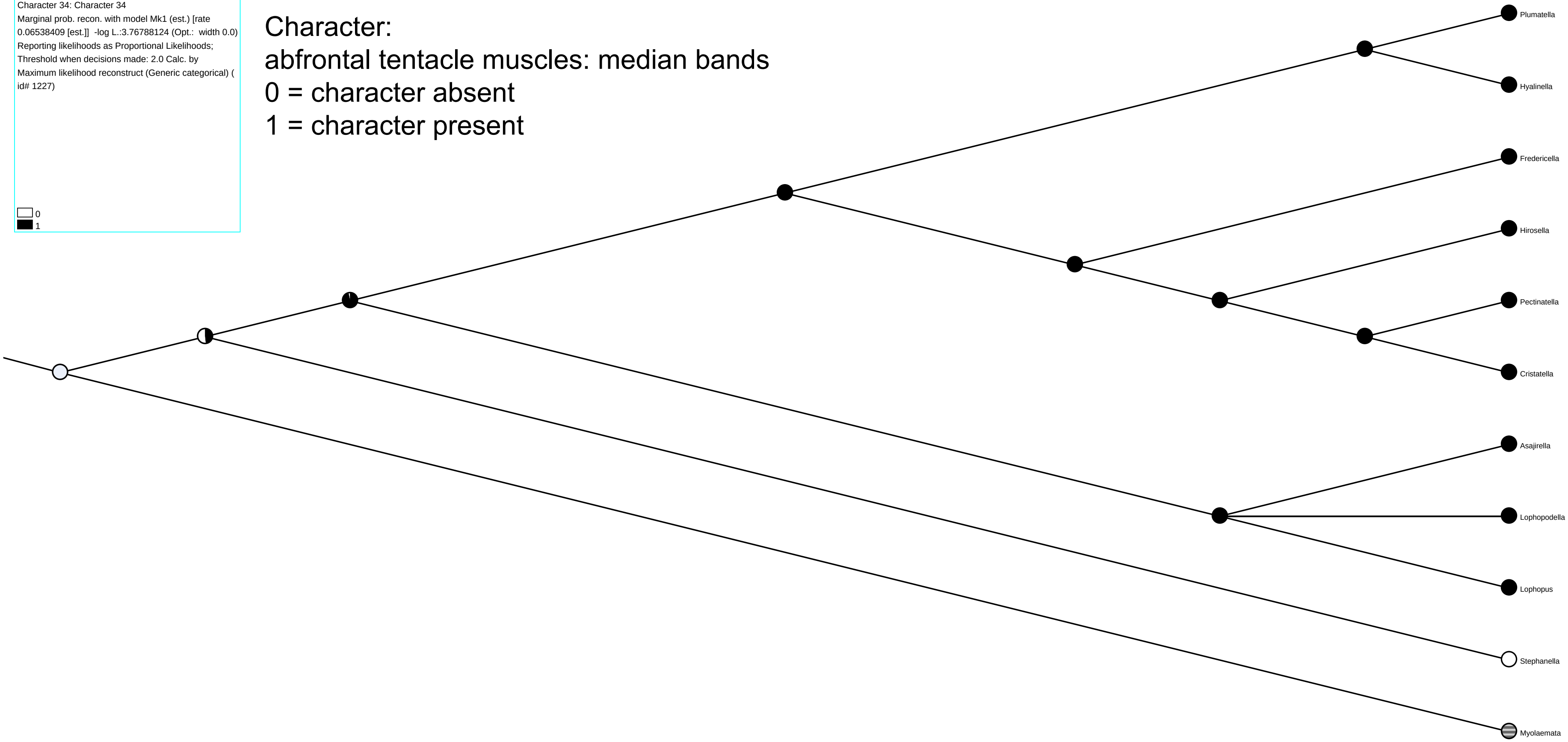

Character 35: Character 35
Marginal prob. recon. with model Mk1 (est.) [rate 0.06538409 [est.]] -log L.:3.76788124 (Opt.: width 0.0) Reporting likelihoods as Proportional Likelihoods; Threshold when decisions made: 2.0 Calc. by Maximum likelihood reconstruct (Generic categorical) (id# 1227)

0
1

Character:
abfrontal tentacle muscles: "stacked" base muscles (opposed to median bands)
0 = character absent
1 = character present

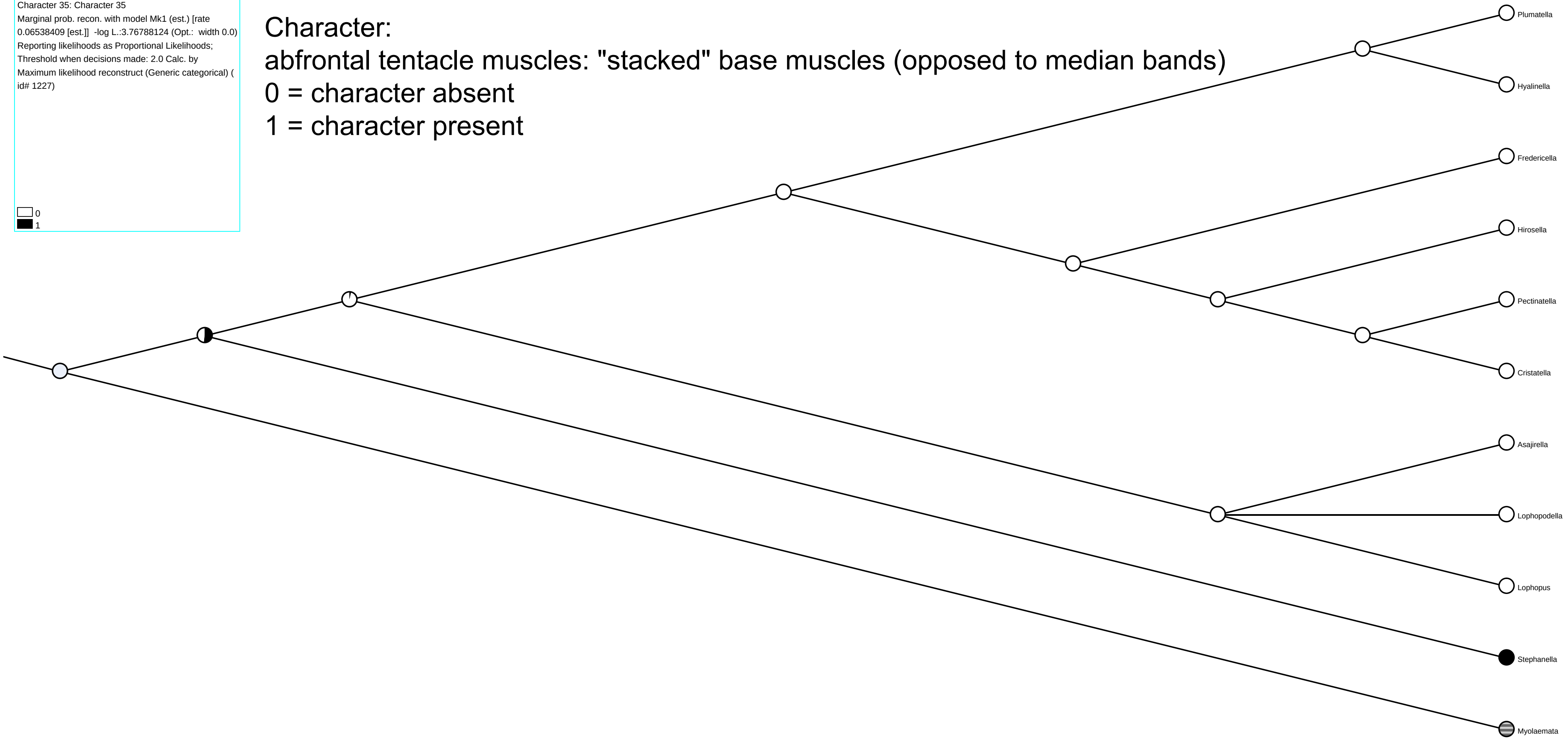

Character:
abfrontal tentacle muscles: gap between base / muscle
0 = character absent
1 = character present

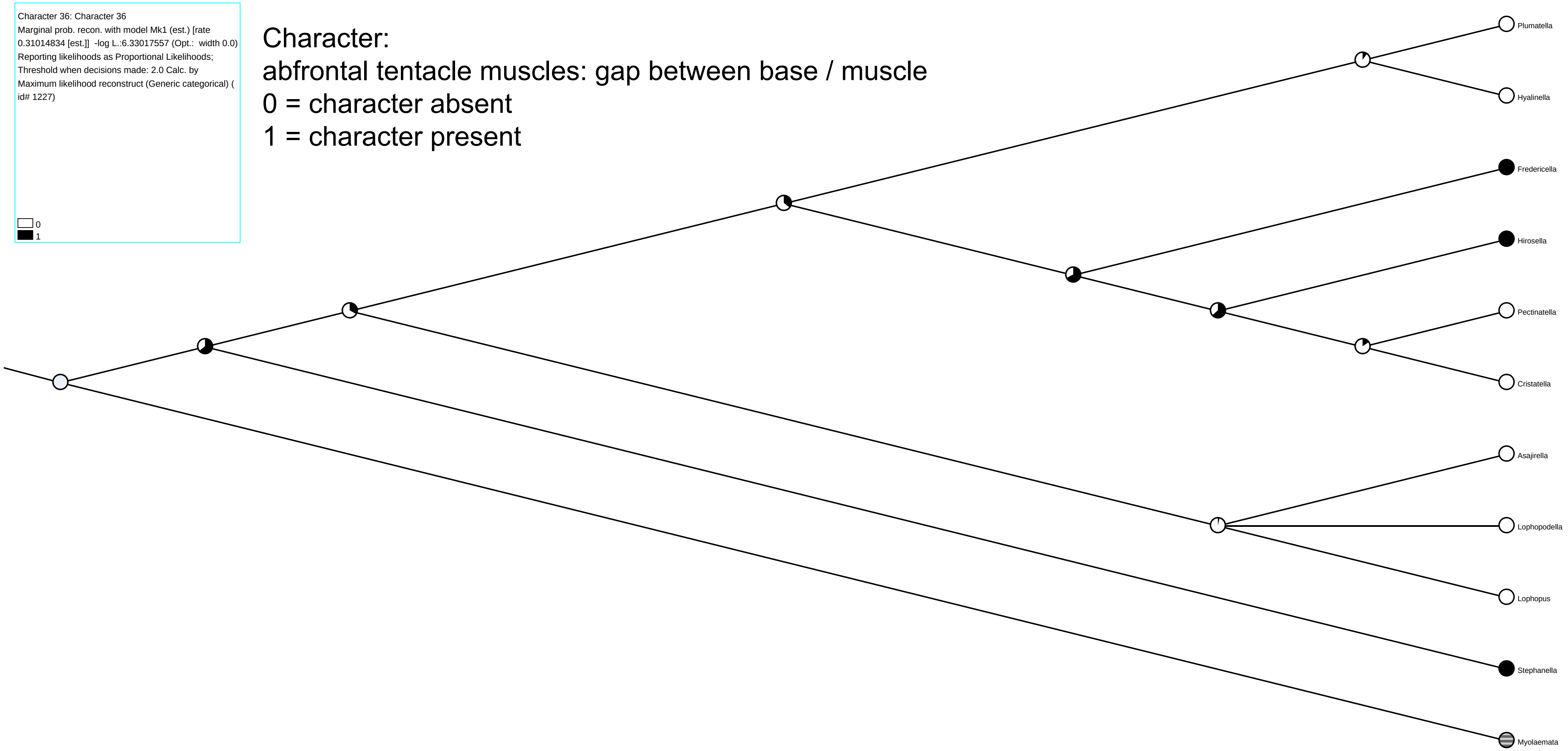

Character 37: Character 37
Marginal prob. recon. with model Mk1 (est.) [rate 0.07796854 [est.]] -log L.:4.29205572 (Opt.: width 0.0) Reporting likelihoods as Proportional Likelihoods; Threshold when decisions made: 2.0 Calc. by Maximum likelihood reconstruct (Generic categorical) (id# 1227)

0
1

Character:
abfrontal tentacle muscles: proximal process
0 = character absent
1 = character present

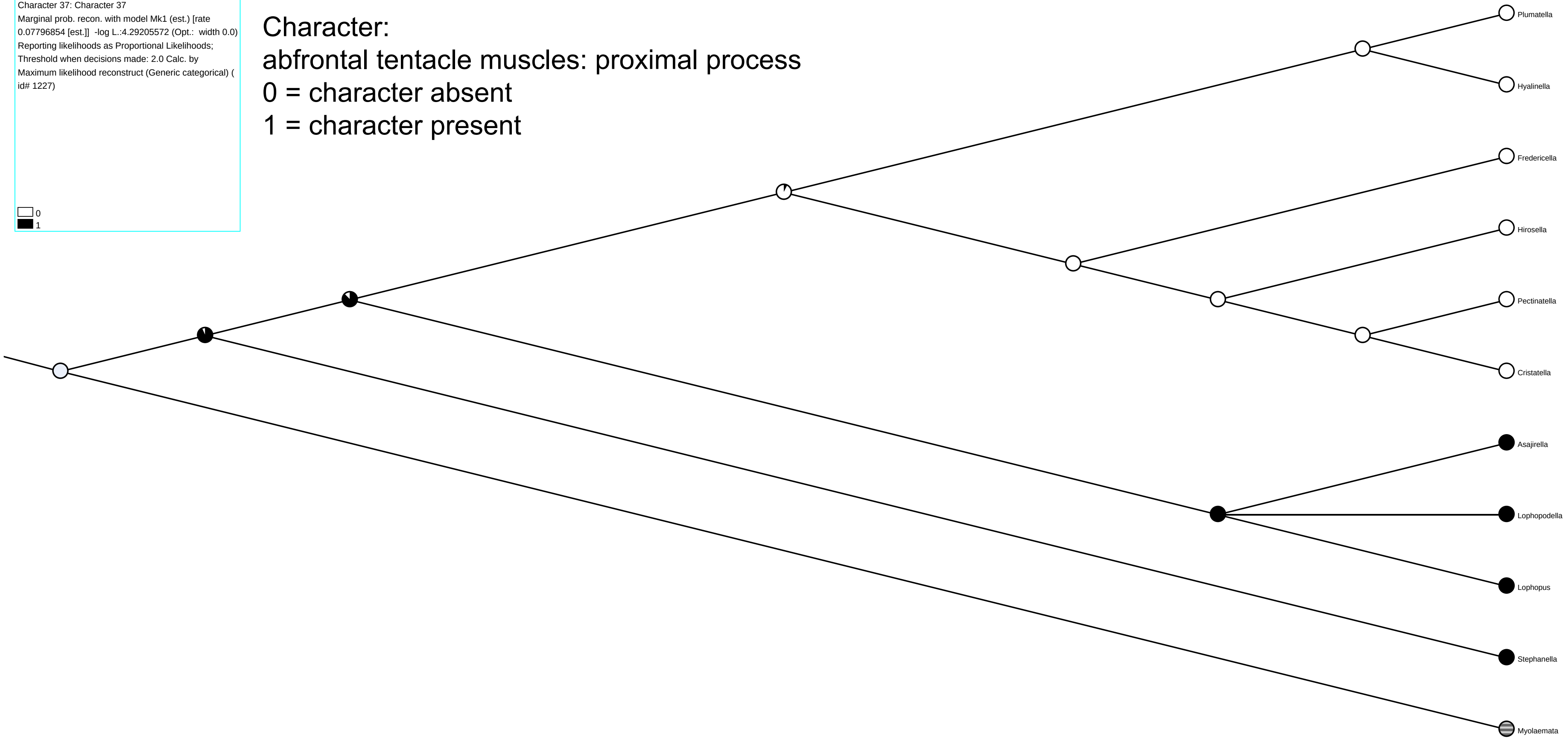

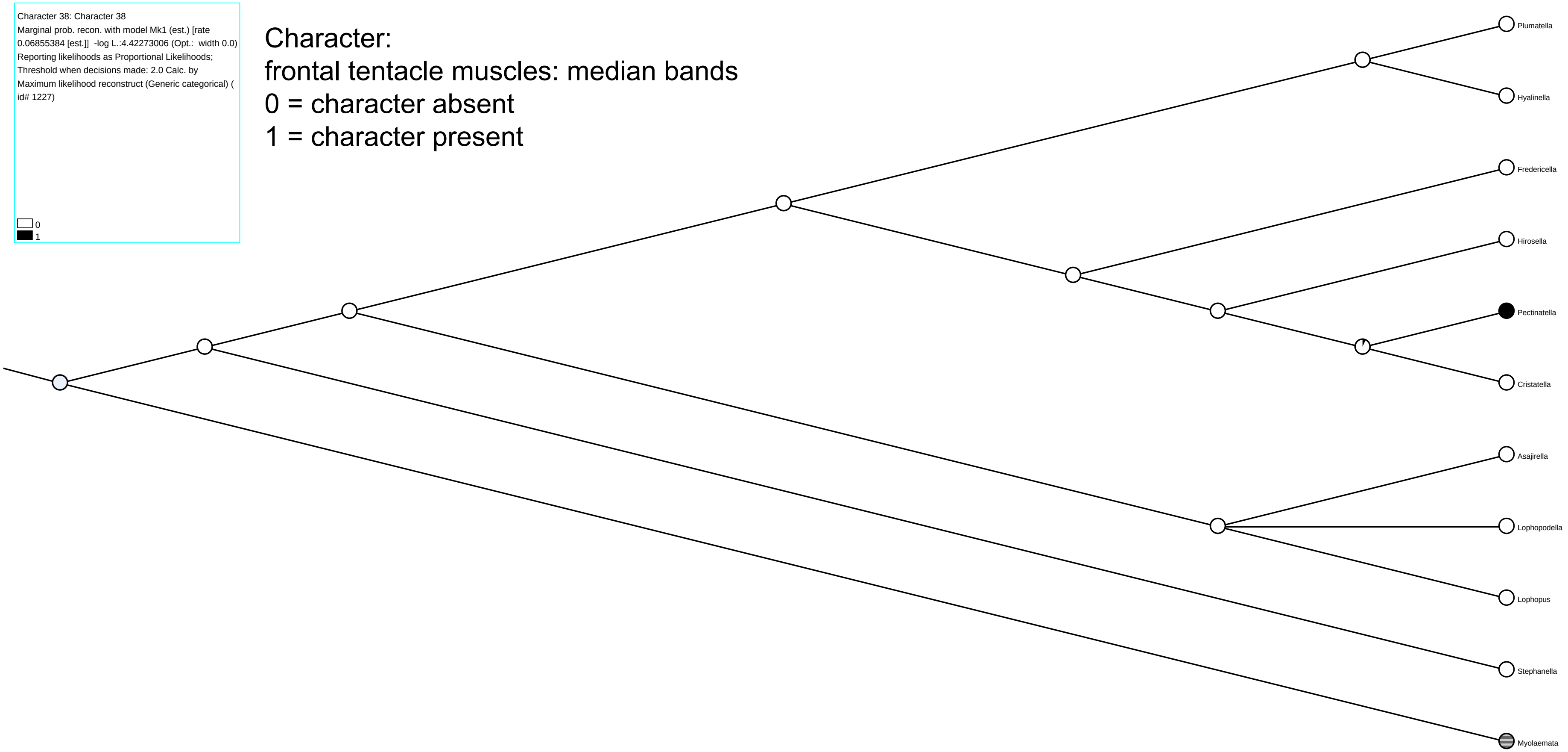
Character 38: Character 38
Marginal prob. recon. with model Mk1 (est.) [rate 0.06855384 [est.]] -log L.:4.42273006 (Opt.: width 0.0) Reporting likelihoods as Proportional Likelihoods; Threshold when decisions made: 2.0 Calc. by Maximum likelihood reconstruct (Generic categorical) (id# 1227)
0
1
Character:
frontal tentacle muscles: median bands
0 = character absent
1 = character present
Plumatella
Hyalinella
Fredericella
Hirosella
Pectinatella
Cristatella
Asajirella
Lophopodella
Lophopus
Stephanella
Myolaemata

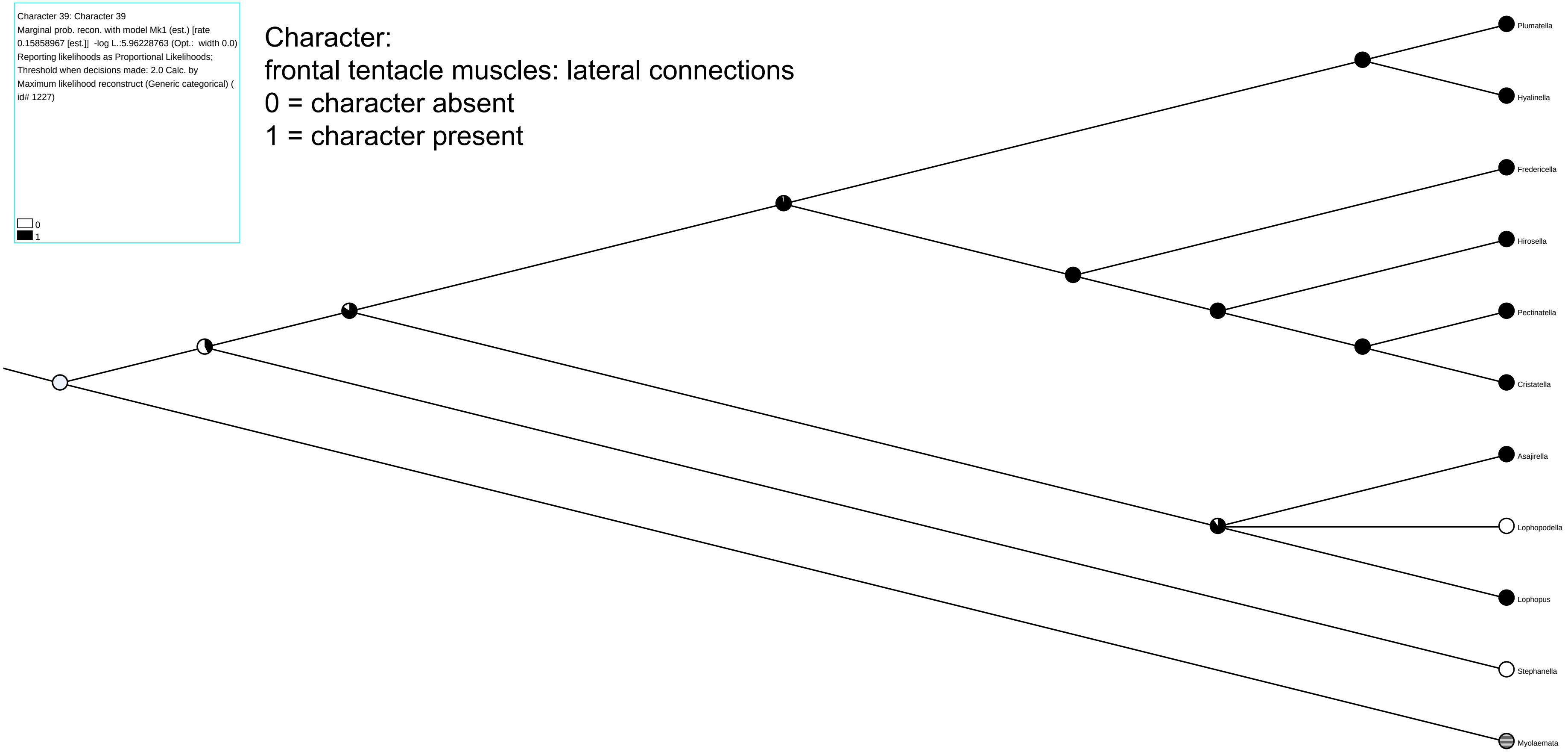
Character 39: Character 39
Marginal prob. recon. with model Mk1 (est.) [rate 0.15858967 [est.]] -log L.:5.96228763 (Opt.: width 0.0) Reporting likelihoods as Proportional Likelihoods; Threshold when decisions made: 2.0 Calc. by Maximum likelihood reconstruct (Generic categorical) (id# 1227)
0
1
Character:
frontal tentacle muscles: lateral connections
0 = character absent
1 = character present
Plumatella
Hyalinella
Fredericella
Hirosella
Pectinatella
Cristatella
Asajirella
Lophopodella
Lophopus
Stephanella
Myolaemata

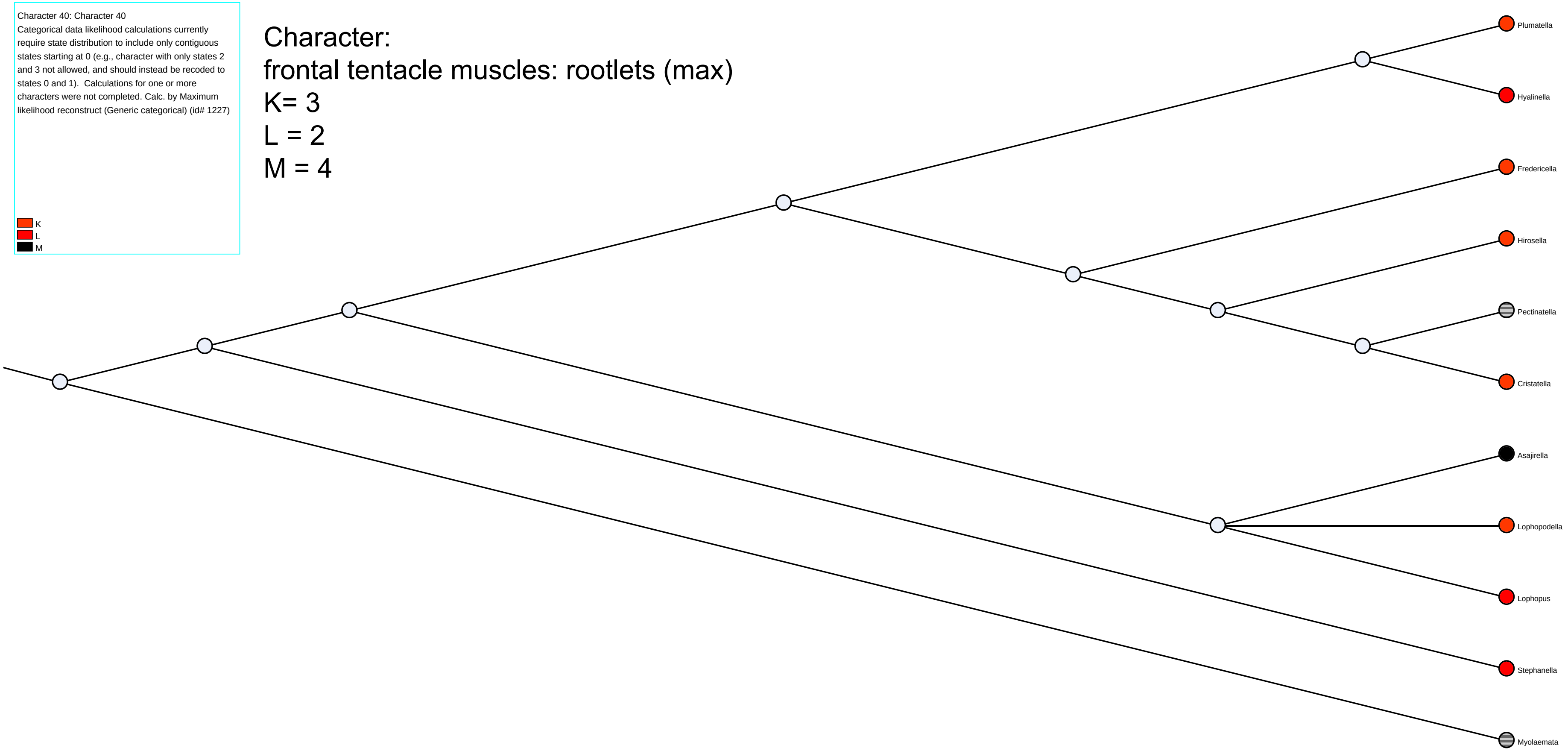
Character 40: Character 40
Categorical data likelihood calculations currently require state distribution to include only contiguous states starting at 0 (e.g., character with only states 2 and 3 not allowed, and should instead be recoded to states 0 and 1). Calculations for one or more characters were not completed. Calc. by Maximum likelihood reconstruct (Generic categorical) (id# 1227)
K
L
M
Character:
frontal tentacle muscles: rootlets (max)
K= 3
L = 2
M = 4
Plumatella
Hyalinella
Fredericella
Hirosella
Pectinatella
Cristatella
Asajirella
Lophopodella
Lophopus
Stephanella
Myolaemata

Character 41: Character 41
Marginal prob. recon. with model Mk1 (est.) [rate 0.3646547 [est.]] -log L.:5.32599375 (Opt.: width 0.0) Reporting likelihoods as Proportional Likelihoods; Threshold when decisions made: 2.0 Calc. by Maximum likelihood reconstruct (Generic categorical) (id# 1227)

0
1

Character:
intertentacular membrane: gap between oral tentacles
0 = character absent
1 = character present

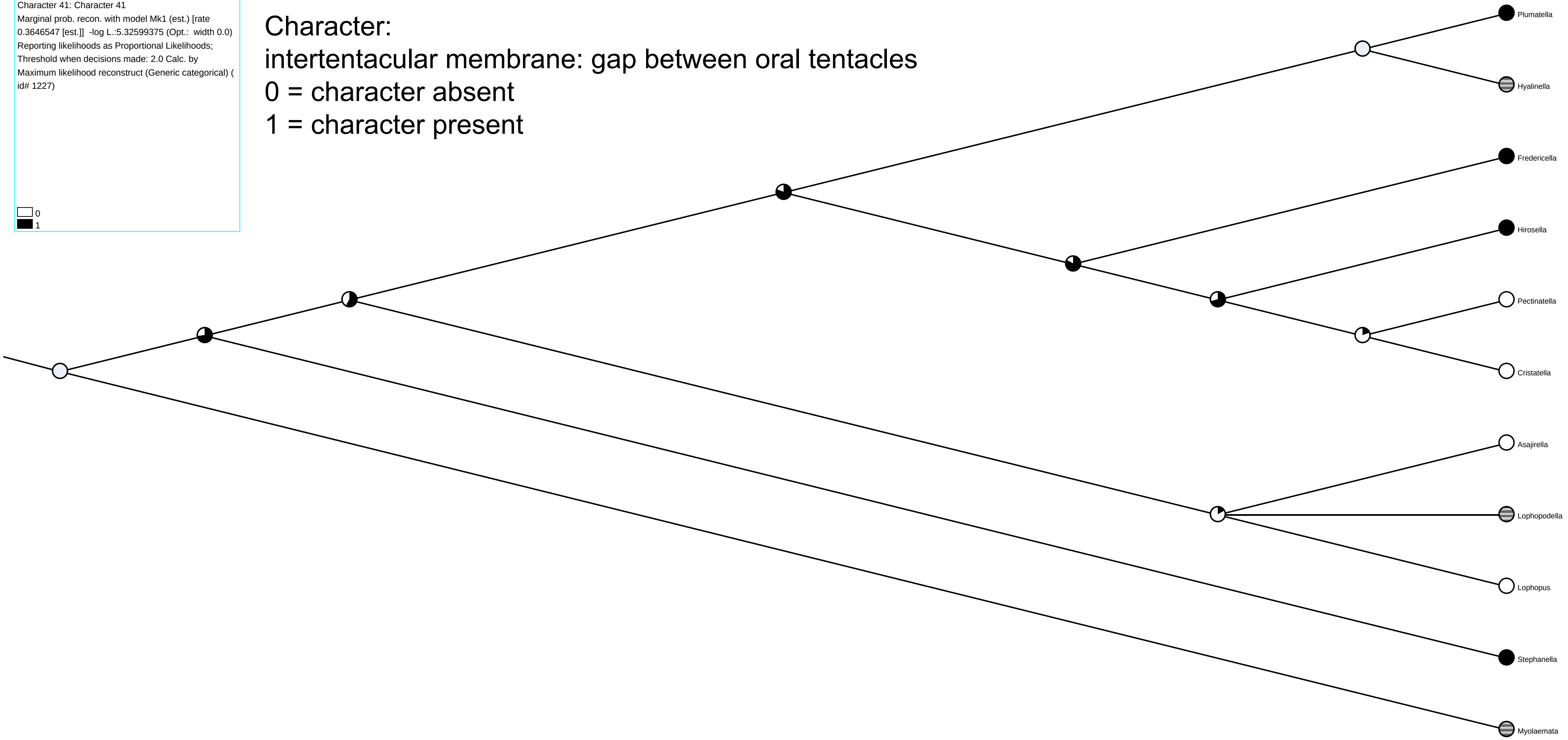

Character:
intertentacular membrane: attached to tentacla via lamella
0 = character absent
1 = character present

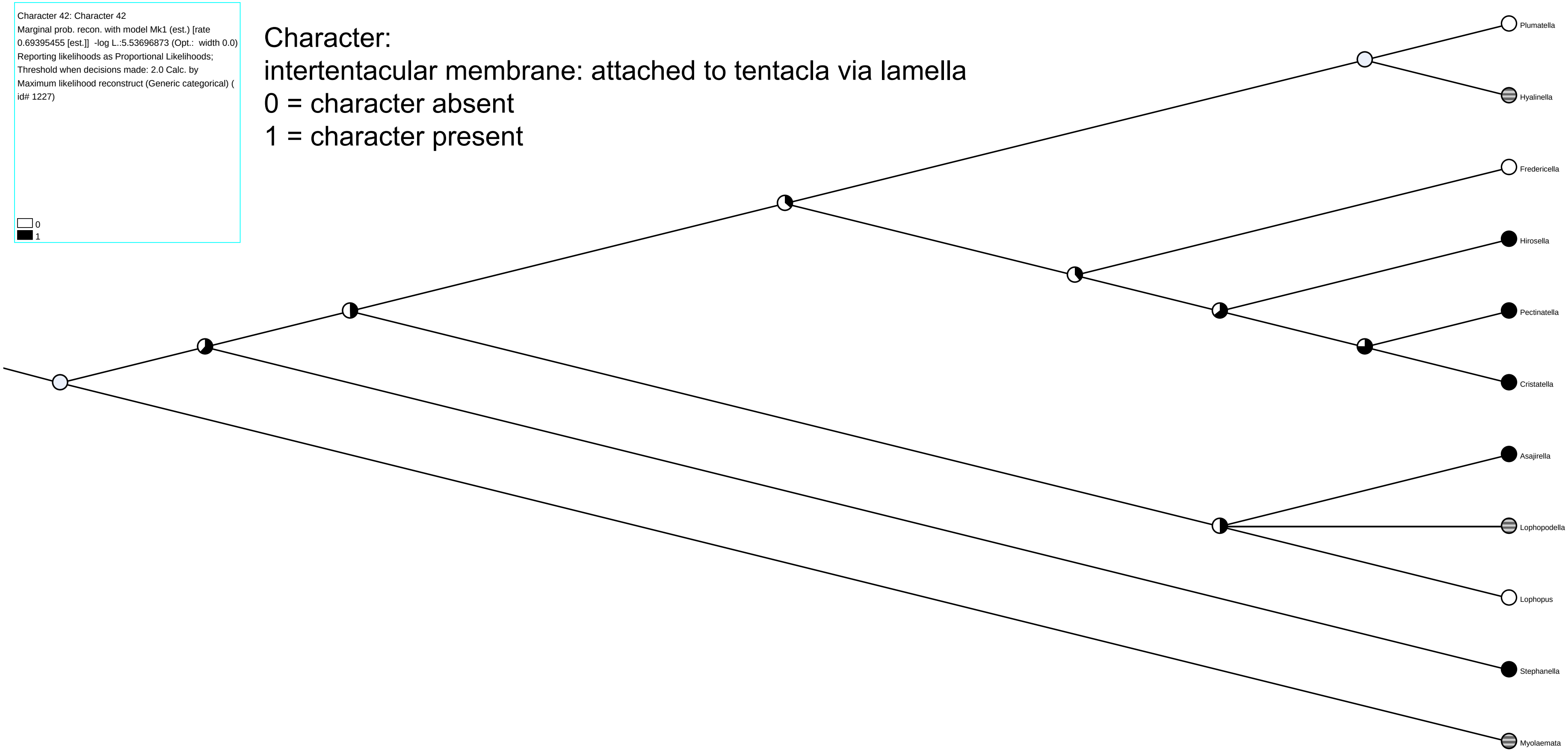

Character 43: Character 43
Categorical data likelihood calculations currently require state distribution to include only contiguous states starting at 0 (e.g., character with only states 2 and 3 not allowed, and should instead be recoded to states 0 and 1). Calculations for one or more characters were not completed. Calc. by Maximum likelihood reconstruct (Generic categorical) (id# 1227)

B
J
N

Character:
central nervous system: ganglionic horns
B = present
J = reduced
N = crescent shaped

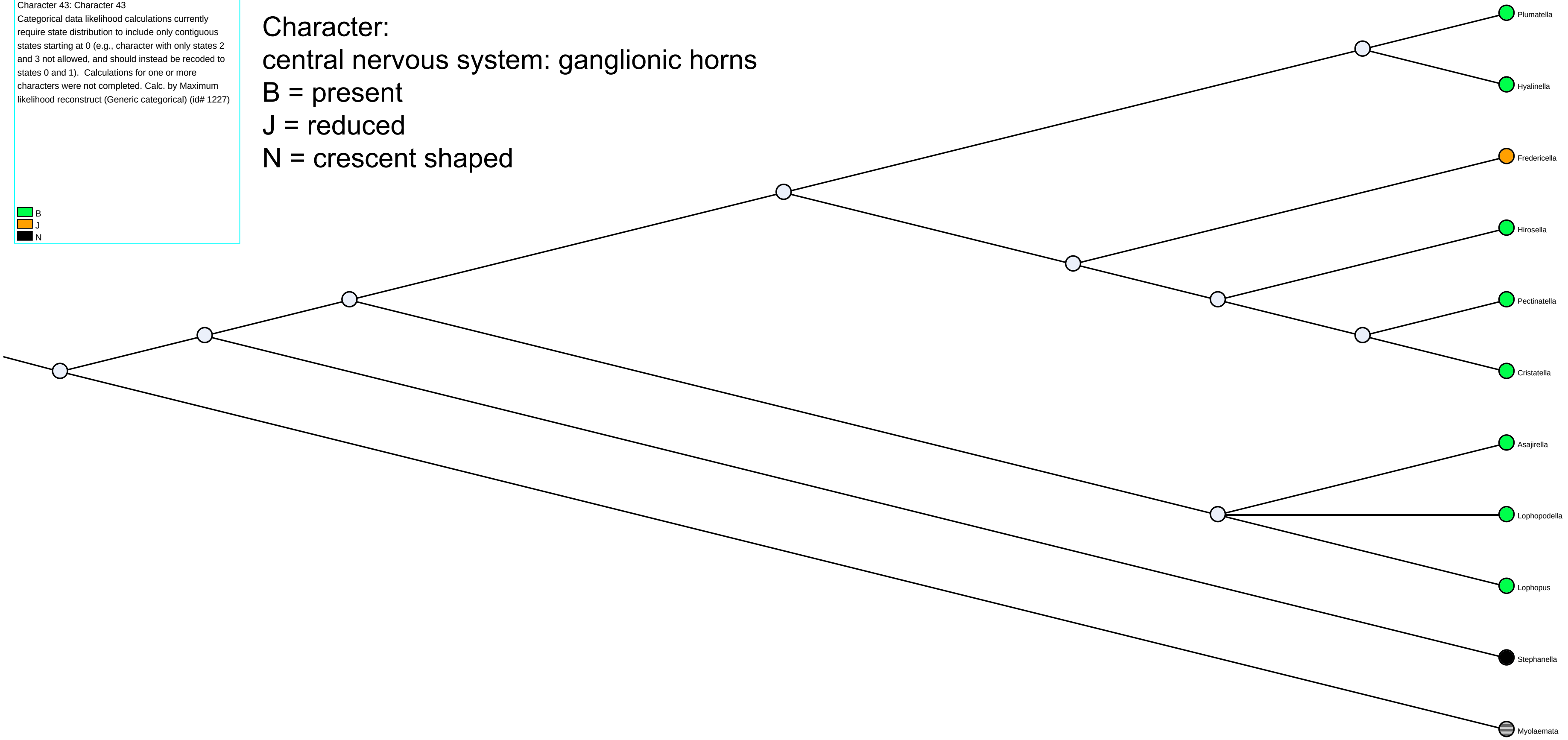

Character 44: Character 44
Marginal prob. recon. with model Mk1 (est.) [rate 0.07441999 [est.]] -log L.:4.34993674 (Opt.: width 0.0)
Reporting likelihoods as Proportional Likelihoods;
Threshold when decisions made: 2.0 Calc. by
Maximum likelihood reconstruct (Generic categorical) (id# 1227)

0
1

Character:
central nervous system: enlargment lumesn / epistomial horns
0 = character absent
1 = character present

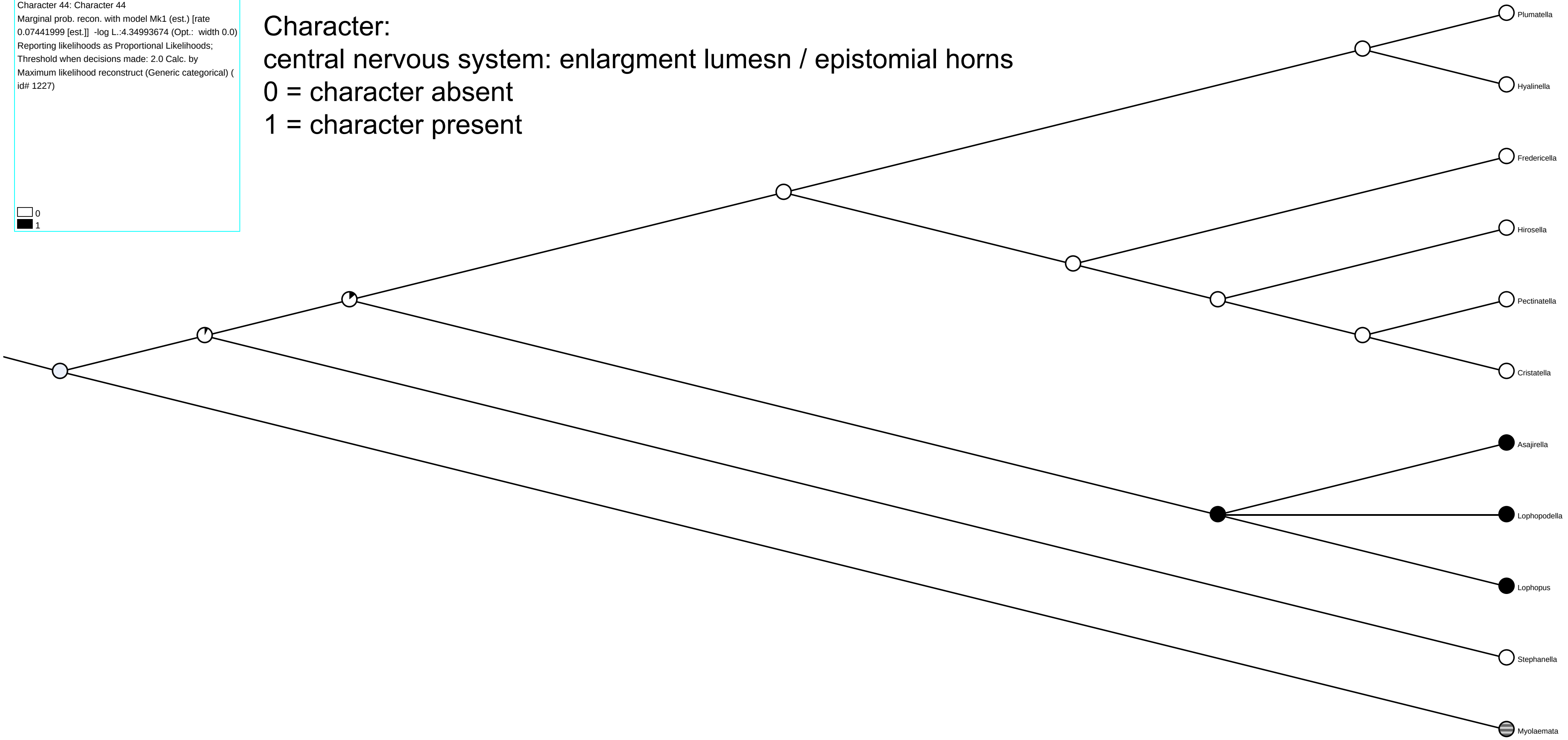

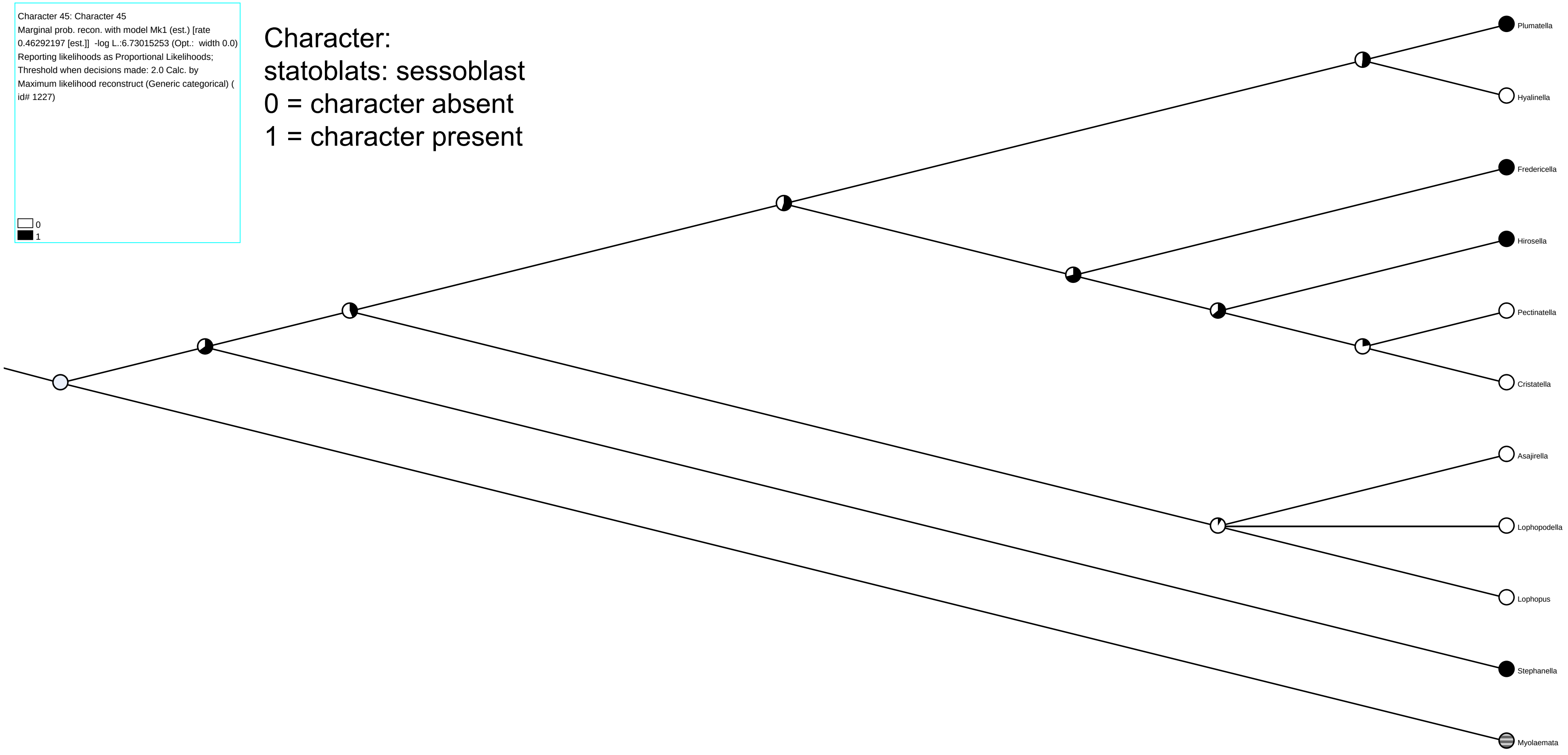
Character 45: Character 45
Marginal prob. recon. with model Mk1 (est.) [rate 0.46292197 [est.]] -log L.:6.73015253 (Opt.: width 0.0)
Reporting likelihoods as Proportional Likelihoods;
Threshold when decisions made: 2.0 Calc. by
Maximum likelihood reconstruct (Generic categorical) (id# 1227)
0
1
Character:
statoblats: sessoblast
0 = character absent
1 = character present
Plumatella
Hyalinella
Fredericella
Hirosella
Pectinatella
Cristatella
Asajirella
Lophopodella
Lophopus
Stephanella
Myolaemata

Character 46: Character 46
Categorical data likelihood calculations currently require state distribution to include only contiguous states starting at 0 (e.g., character with only states 2 and 3 not allowed, and should instead be recoded to states 0 and 1). Calculations for one or more characters were not completed. Calc. by Maximum likelihood reconstruct (Generic categorical) (id# 1430)

Character:
statoblats: sessoblast development
0 = no sessoblast
O = cystigenic
P = deutoplasmic

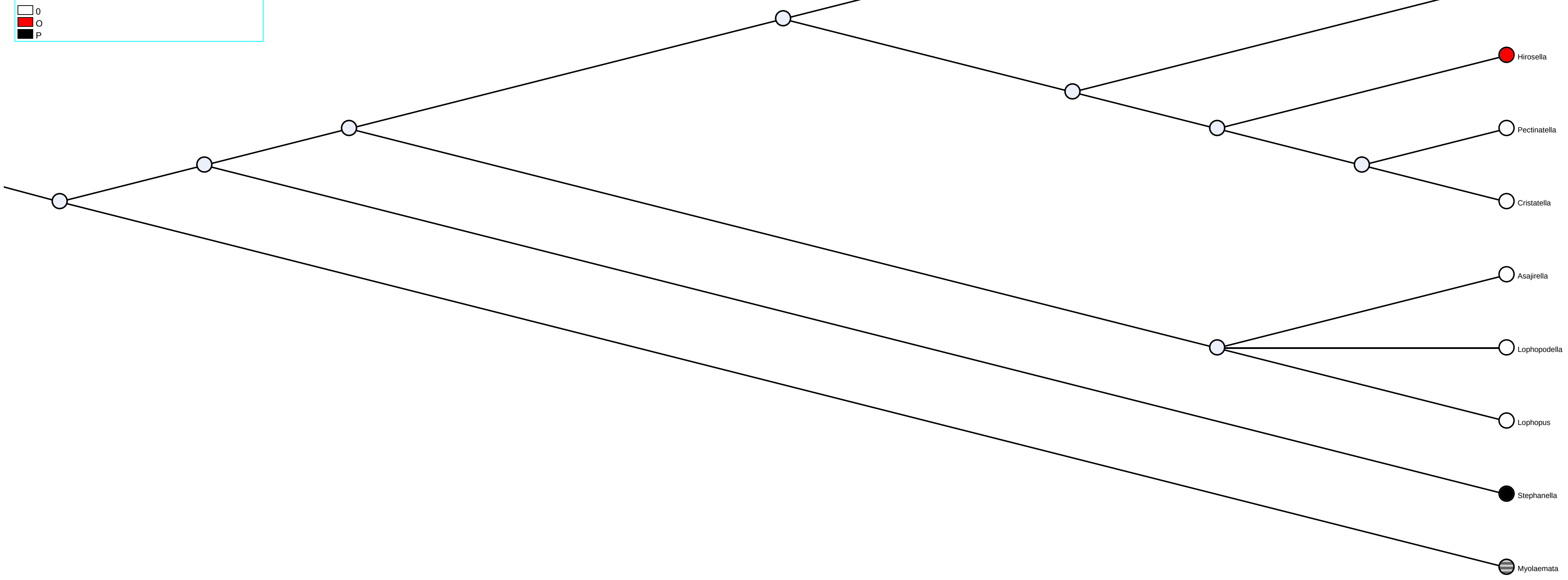

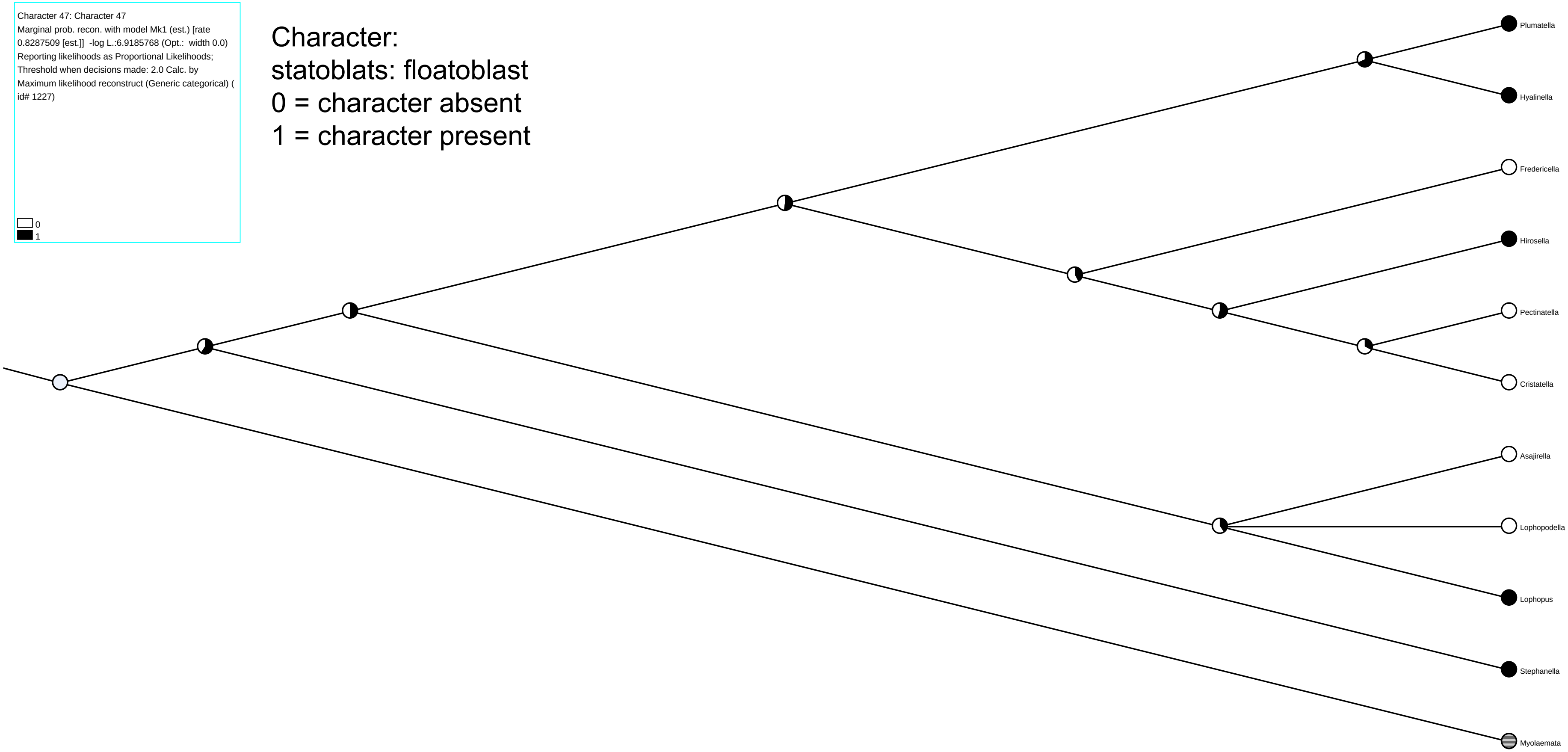
Character 47: Character 47
Marginal prob. recon. with model Mk1 (est.) [rate 0.8287509 [est.]] -log L.:6.9185768 (Opt.: width 0.0) Reporting likelihoods as Proportional Likelihoods; Threshold when decisions made: 2.0 Calc. by Maximum likelihood reconstruct (Generic categorical) (id# 1227)
0
1
Character:
statoblats: floatoblast
0 = character absent
1 = character present
Plumatella
Hyalinella
Fredericella
Hirosella
Pectinatella
Cristatella
Asajirella
Lophopodella
Lophopus
Stephanella
Myolaemata

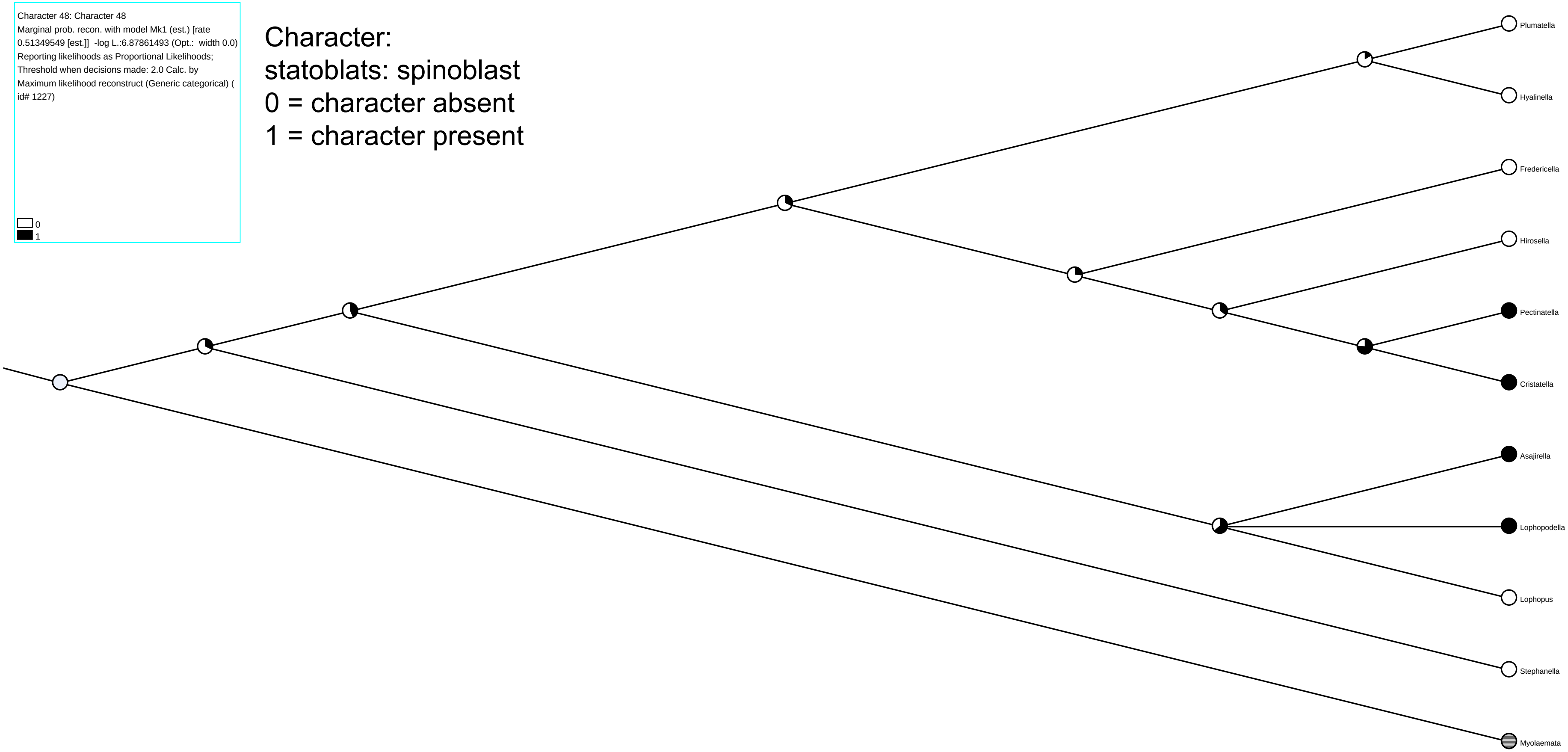

Character 48: Character 48
Marginal prob. recon. with model Mk1 (est.) [rate 0.51349549 [est.]] -log L.:6.87861493 (Opt.: width 0.0) Reporting likelihoods as Proportional Likelihoods; Threshold when decisions made: 2.0 Calc. by Maximum likelihood reconstruct (Generic categorical) (id# 1227)
0
1
Character:
statoblats: spinoblast
0 = character absent
1 = character present
Plumatella
Hyalinella
Fredericella
Hirosella
Pectinatella
Cristatella
Asajirella
Lophopodella
Lophopus
Stephanella
Myolaemata

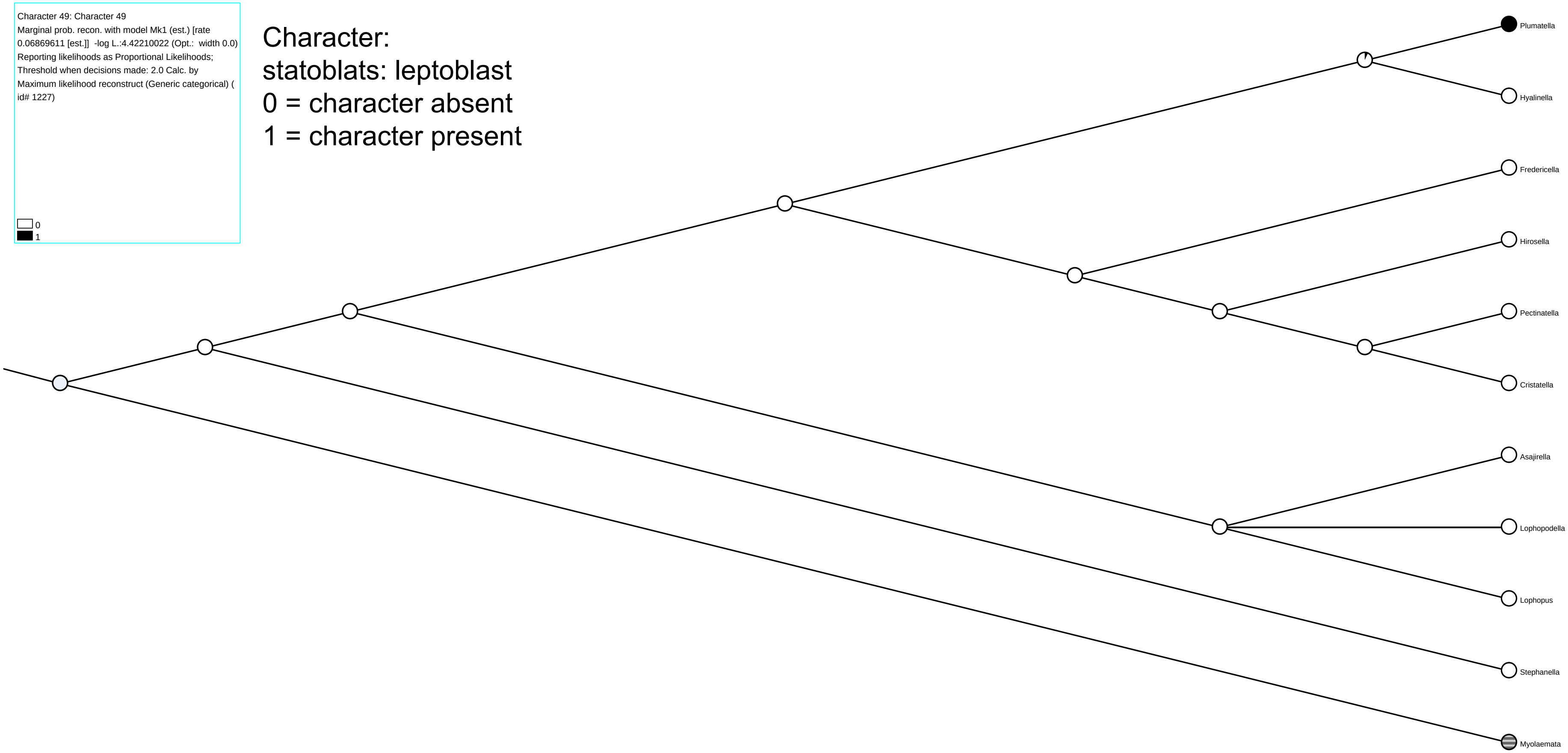
Character 49: Character 49
Marginal prob. recon. with model Mk1 (est.) [rate 0.06869611 [est.]] -log L.:4.42210022 (Opt.: width 0.0)
Reporting likelihoods as Proportional Likelihoods;
Threshold when decisions made: 2.0 Calc. by
Maximum likelihood reconstruct (Generic categorical) (id# 1227)
0
1
Character:
statoblats: leptoblast
0 = character absent
1 = character present
Plumatella
Hyalinella
Fredericella
Hirosella
Pectinatella
Cristatella
Asajirella
Lophopodella
Lophopus
Stephanella
Myolaemata

Character 50: Character 50
Marginal prob. recon. with model Mk1 (est.) [rate 0.06949501 [est.]] -log L.:4.41706428 (Opt.: width 0.0) Reporting likelihoods as Proportional Likelihoods; Threshold when decisions made: 2.0 Calc. by Maximum likelihood reconstruct (Generic categorical) (id# 1227)

0
1

Character:
statoblats: piptoblast
0 = character absent
1 = character present

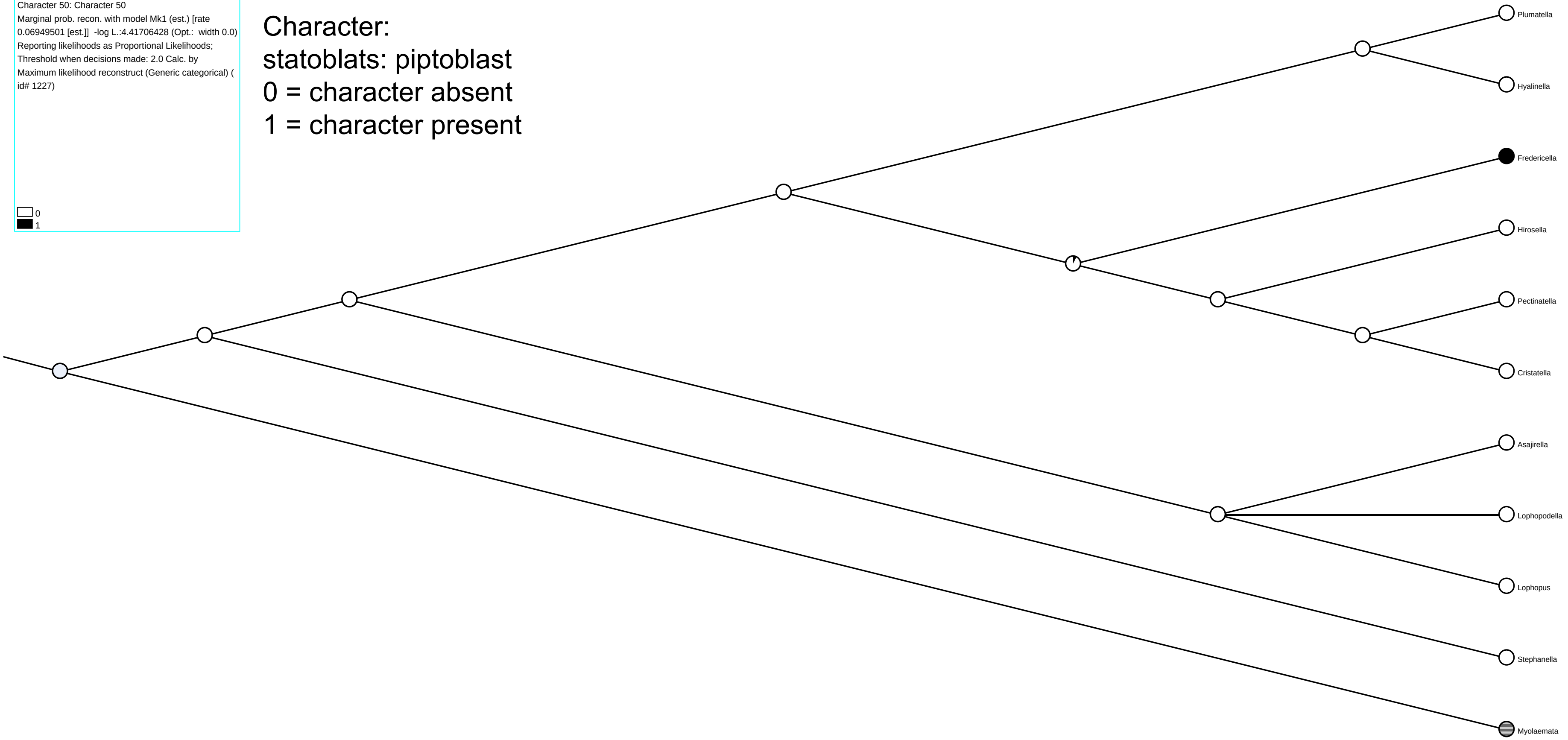

Character 51: Character 51
Categorical data likelihood calculations currently require state distribution to include only contiguous states starting at 0 (e.g., character with only states 2 and 3 not allowed, and should instead be recoded to states 0 and 1). Calculations for one or more characters were not completed. Calc. by Maximum likelihood reconstruct (Generic categorical) (id# 1227)

1

Character:
larvae: cilliated mantle larvae
0 = character absent
1 = character present

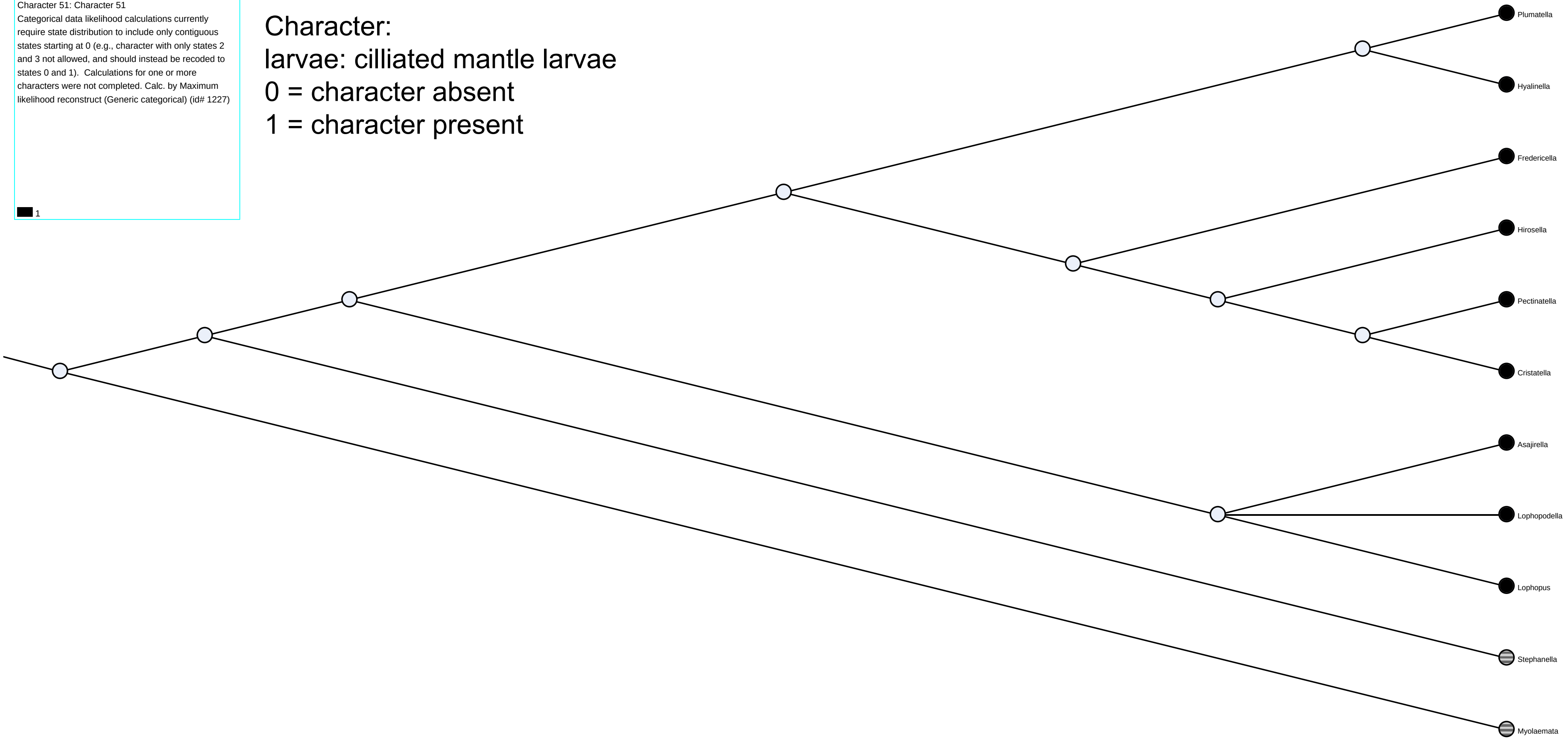

Character 52: Character 52
Categorical data likelihood calculations currently require state distribution to include only contiguous states starting at 0 (e.g., character with only states 2 and 3 not allowed, and should instead be recoded to states 0 and 1). Calculations for one or more characters were not completed. Calc. by Maximum likelihood reconstruct (Generic categorical) (id# 1227)

L
M
Q

Character:
larvae: number of larval polipides
L = 2
M = 4
Q = 1

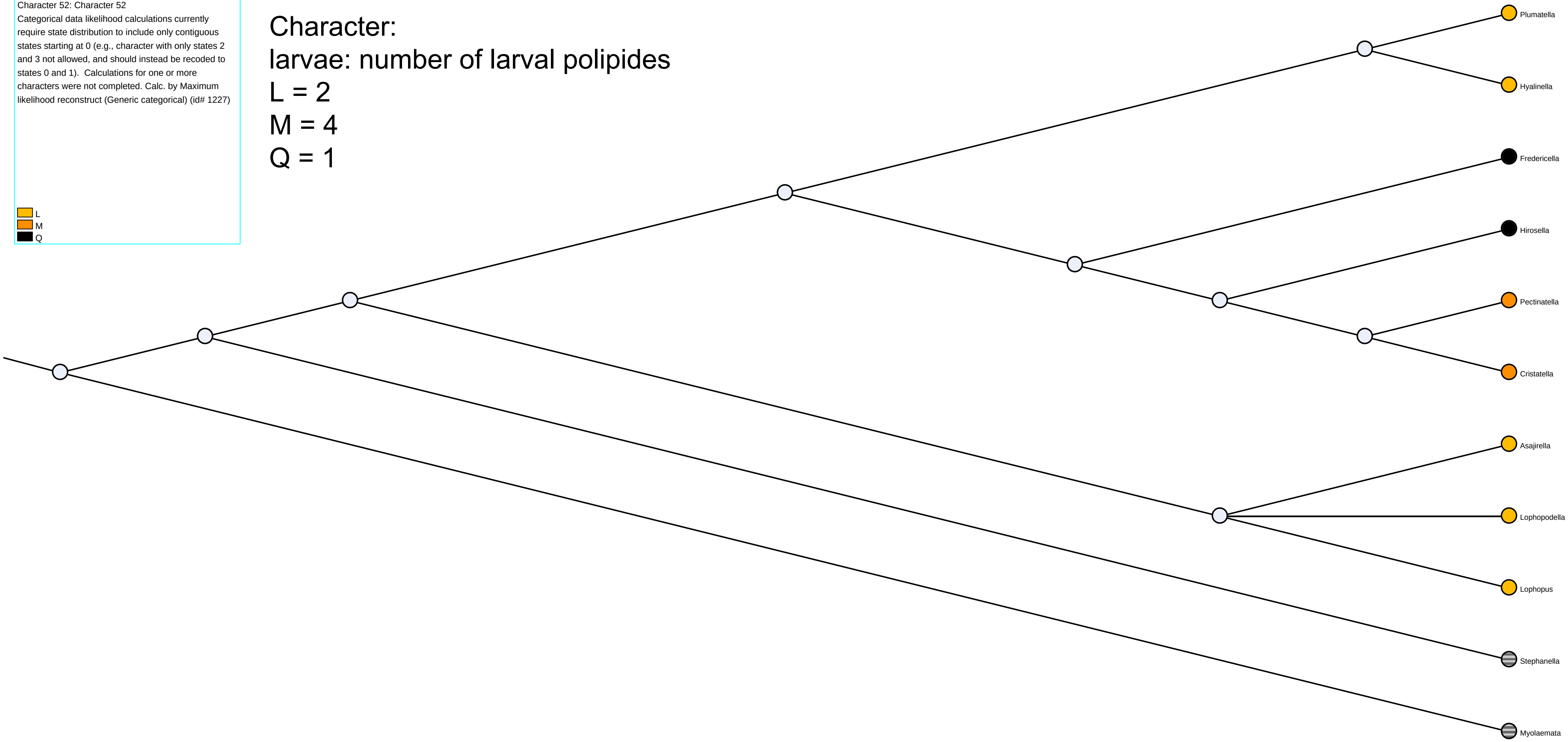

Supplement: Supplementary file 1 — Supplementary Material 1 [file 41598_2026_40223_MOESM1_ESM.pdf]
