# Supplementary material for: Morphological character evolution and ancestral state reconstruction in phylactolaemate bryozoans
Source: Sci Rep. 2026 Mar 23;16:15106. doi: 10.1038/s41598-026-40223-0 (PMC13171971; doi:10.1038/s41598-026-40223-0)
Supplement: Supplementary file 2 — Supplementary Material 2 [file 41598_2026_40223_MOESM2_ESM.pdf]

Supplemental Figure 1: Ancestral state reconstruction of all characters. Ancestral states were reconstructed independently under the MK1 model. For each tree, pie charts at the nodes indicate the relative likelihoods of alternative character states. Trees are shown in the same topology as in the main phylogeny (Fig. 1).
